# Crystal-to-crystal solar energy storage in a hydrogen bonded dianthracene lattice retrocyclized by heat or e-beam

**DOI:** 10.1039/d6sc05227d

**Published:** 2026-10-23

**Authors:** Eric Schreiber, Hovnan Simonyan, Madison E. Logelin, Aleksa K. Banki, Madeleine Y. Popofsky, Christopher Kim, Amymarie K. Bartholomew

**Affiliations:** a Department of Chemistry, Yale University New Haven Connecticut 06520 USA amymarie.bartholomew@yale.edu

## Abstract

Solid-state (SS) systems for molecular solar thermal (MOST) energy storage are desirable for their simplicity and higher energy storage density compared to solution-based MOST systems, but it is difficult to design systems that achieve both high conversion to the metastable photoproduct and undergo thermally-driven release of the stored heat without melting. Anthracene (An) photocycloaddition to dianthracene (diAn) can proceed to high conversion in the solid state, but few crystalline Ans are appropriately aligned for dimerization, and even fewer diAns thermally retrocyclize without melting. By combining 9-pyridyl anthracene and 9-anthracenecarboxylic acid, we form hydrogen-bonded (H-bonded) and π-stacked chains that undergo high-conversion single-crystal-to-single-crystal photocycloaddition under simulated sunlight. Disordered packing in the lattice produces a mixture of the pyridyl-carboxylate “heterodimer,” the pyridyl homodimer, and the carboxylic acid homodimer. Calorimetry reveals that the H-bonded diAn product has a large separation between its thermal retrocyclization and melting temperatures, allowing the material to be cycled in the solid state. The H-bonded diAn lattice is also shown to undergo crystal-to-crystal retrocyclization under e-beam exposure. This work presents an easily-assembled all-SS crystal-to-crystal cyclable MOST system and demonstrates that H-bonding can be leveraged to increase the gap between thermal retrocyclization and melting.

## Abstract 2

A 1D H-bonded lattice of 9-anthracenecarboxylic acid and 9-pyridyl anthracene undergoes [4 + 4]-photocycloaddition in the solid-state. Thermal and e-beam-induced retrocyclization proceeds in a crystal-to-crystal fashion.

## Graphical abstract

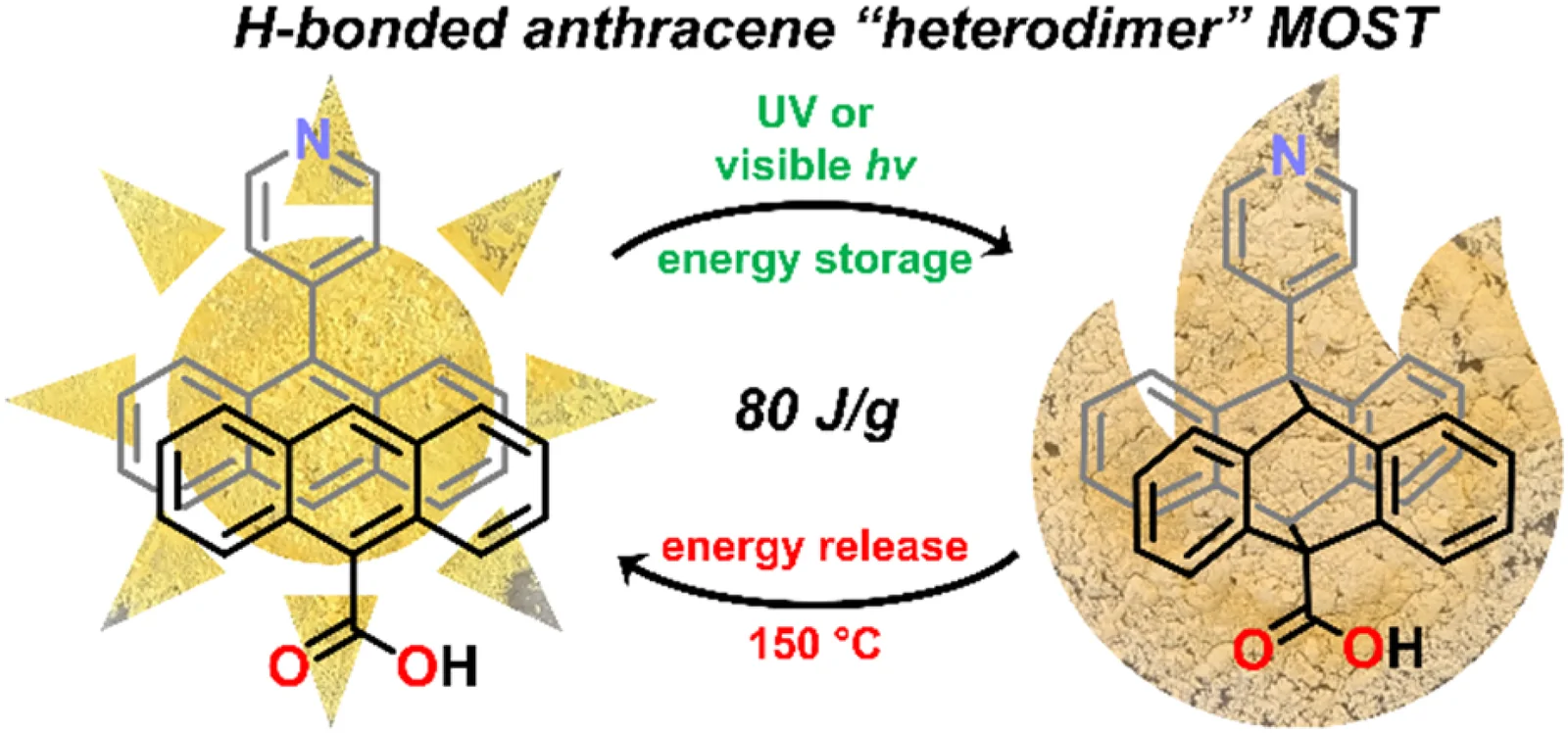

## Introduction

Molecular solar-thermal (MOST) energy storage is an emerging technology that directly converts photon energy into metastable chemical bonds for subsequent release as heat upon bond cleavage.^1–5^ All-solid-state (SS) cyclable MOST systems promise higher energy densities and simpler device architectures than solution-phase systems.^3^ However, it is difficult to design MOST platforms that are SS cyclable, as the majority of organic photoswitches cannot achieve high SS conversion owing to either large volume changes, unsuitable crystal packing, or melting before thermally activated heat-release (*e.g.* retrocyclization, back-isomerization) is complete. When the thermal activation temperature and melting point are not well-separated, the endothermic melting process consumes some or all of the stored energy, lowering or completely nullifying the energy released.^3^ Additionally, many SS MOST systems are only active under UV light irradiation, with few platforms demonstrating energy storage using visible light or direct/simulated sunlight.^6–10^

Organic photoswitches such as norbornadienes,^11–14^ dihydroazulenes,^15–17^ and azoarenes^7,18–21^ have been studied for solution-phase MOST applications, but rarely in the SS. Strategies for achieving neat MOST cycling with these platforms have focused on mitigating their low SS conversions. For example, azoarenes designed to undergo a solid–liquid phase transition upon photoisomerization achieve full conversion and extra energy release from solidification upon back-isomerization, but in turn require a sealed system to prevent loss of the liquid photoproduct.^18–20,22–28^ Another approach incorporates the MOST active species into crystalline frameworks,^29–32^ polymers,^33,34^ or tethered to substrates,^35^ achieving high conversion as a result of increased space for significant volume changes, but also decreasing energy density by effectively diluting the active species. Some elegant azobenzene^36^ and styrylpyrylium^6^ systems have been developed that can achieve both high conversion in the solid state and thermally activated energy release without melting, but broadly-applicable new strategies to access SS, visible light-responsive, energy dense MOST materials are still needed to progress toward practical deployment of photothermal energy storage devices.

Anthracene (An) is an underexplored platform for SS MOST material design. The earliest reports of An [4 + 4] photocycloaddition to form dianthracene (diAn) used sunlight, making this a suitable platform for direct solar energy capture.^37–39^ Additionally, many simple An derivatives absorb visible light and, when aligned correctly, can dimerize in the solid state owing to the small volume change of the transformation.^40–45^ DiAns subsequently retrocyclize exothermically upon heating or under short-wave UV light (<300 nm).^46–48^

Despite decades of study, only four types of diAn systems have been studied for MOST.^49–52^ The first such report, by Moth-Poulsen and coworkers, employs tethered 1,2-dianthrylbenzenes, achieving high solution-state cyclability by tethering the An units (Fig. 1).^49^ This group subsequently studied an acenaphthene-linked system that exhibits long storage lifetimes despite substantial strain in the photodimerized state. More recently, the Han group characterized a novel SS An/diAn MOST system using 9-cyanoanthracenes featuring electron donors at the 10-position with relatively low retrocyclization temperatures (*T*_retro_); however, high-conversion SS photocycloaddition required a bespoke ball-milling irradiation setup, preventing straightforward cycling (Fig. 1).^50^ The Han An/diAn system is notable for its auto-catalyzed thermally activated heat-release, which circumvents the challenge of needing impractically high sustained temperatures to release heat stored in an all-SS MOST system. Of particular relevance to this study, the Hutchins group reported one partial–conversion cycle of the 9-anthracenecarboxylic acid (An^COOH^) dimer, diAn^COOH2^, as part of a lattice in which An^COOH^ was aligned for dimerization by hydrogen bonding (H-bonding) to 4-stilbazole (Fig. 1).^9^

**Fig. 1:**
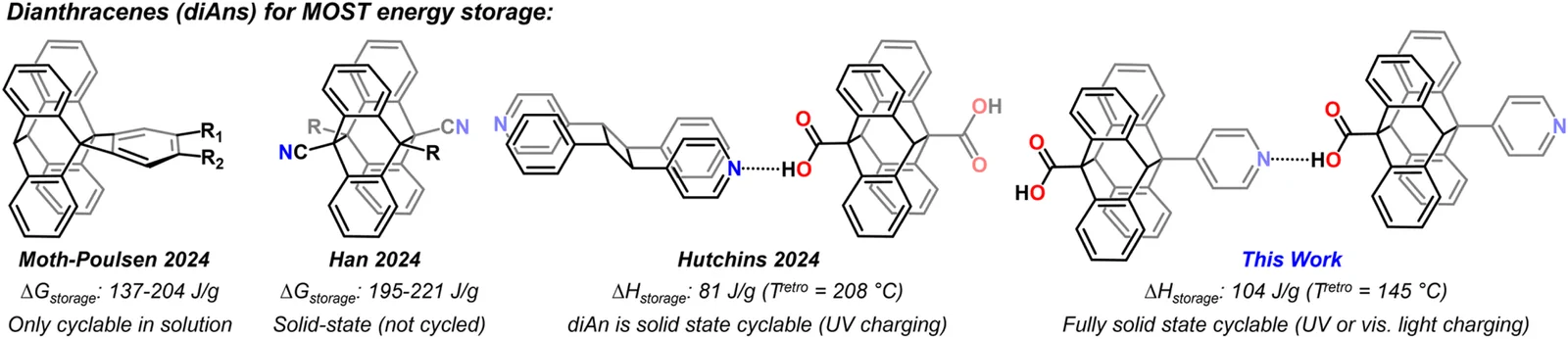
DiAn-based MOST energy storage materials.

Here, we present a new diAn material for a MOST active species based on 9-anthracenecarboxylic acid (An^COOH^) and 9-pyridylanthracene (An^Py^). An^COOH^ and An^Py^ cocrystallize in a 1D π-stacking/hydrogen-bonded (H-bonded) lattice (1), prearranging the Ans to readily dimerize in a single crystal-to-single crystal (SC–SC) transformation using UV light, visible light, or simulated sunlight. The photocyclized product, 1-Dimer, is comprised primarily of the heterodimer, diAn^Py/COOH^ (2), along with minor components of both homodimers, diAn^Py2^ (3) and diAn^COOH2^ (4), resulting from crystallographically modellable packing disorder in the monomer lattice 1. Pure recrystallized samples of 3 and 4 begin melting before thermal retrocyclization is complete and cannot be subsequently re-dimerized in the solid state. H-bonded lattice 1-Dimer requires a substantially lower activation temperature than any of its isolated components, likely due to strain induced by crystalline confinement, allowing for dimer cleavage well below the melting point of the H-bonded monomer lattice 1.

1-Dimer is converted back to crystalline 1 upon annealing, thus the 1/1-Dimer system is straightforwardly cyclable in the solid state and achieves 90% charging under one sun of simulated solar irradiation in nine hours. The energy storage density of 1-Dimer is 82–104 J g^−1^, depending on calorimetry scan rate. This density is comparable to that of the only previously reported SS cyclable diAn system (81 J g^−1^), with the added benefits of higher conversion, lower retrocyclization temperature, and visible light charging.^9^ The energy density of 1-Dimer is greater than that of the only other visible light SS cyclable system, a styrylpyrylium lattice from the Han group (6–51 J g^−1^).^6^ More broadly, this report represents the first example of an anthracene heterodimer MOST system and serves as an example of how intermolecular forces can facilitate cyclable SS photothermal energy storage.

Lastly, given the crystal-to-crystal thermal retrocyclization of 1-Dimer to 1 and recent reports of electron beam (e-beam) driven crystal-to-crystal retrocyclization of some cyclobutanes,^53–55^ we carried out microcrystal electron diffraction (MicroED) studies of 1-Dimer. These studies show that 1-Dimer undergoes an e-beam driven crystal-to-crystal retrocyclization back to 1.

## Results and discussion

### Synthesis and characterization of the anthracene heterodimer species, 1-Dimer

Formation of An crystals with appropriate packing to promote dimer formation in the solid state is a low-probability endeavour, relying on chance to generate materials where Ans π-stack face-to-face, with the 9,10-carbon atoms overlapping within 4.2 Å.^56^ One potential method to promote such ordering is to install functional groups that engage in additional intermolecular interactions.^57^ H-bonding is an attractive potential route to promote intermolecular ordering for this class of compounds, as multiple H-bond donor/acceptor-functionalized Ans are reported or commercially available.^9,58–61^ Indeed, several examples have demonstrated how H-bonding in solid state structures can facilitate π-stacking in An crystals, enabling photocycloaddition in the solid-state.^62–66^

To leverage H-bonding and promote long-range order in an An-based crystal, we paired 9-functionalized Ans with acidic and basic substituents. An^COOH^ is commercially available, and 9-pyridyl anthracene (An^Py^) was synthesized *via* Suzuki cross-coupling using 9-bromoanthracene *via* a reported procedure on multigram scales.^58^ Recrystallization of an equimolar mixture of An^Py^ and An^COOH^ from THF solution produces yellow block crystals suitable for single-crystal X-ray diffraction (SCXRD) with a ∼1 : 1 stoichiometry of An^Py^ and An^COOH^ as assessed by ^1^H NMR (Fig. S1 and S2).

Solution and refinement of the crystal structure of the An^Py^/An^COOH^ cocrystals (1) reveal that the molecules arrange in 1D H-bonded chains with head-to-tail π-stacking between An partners. Analysis of the structure solved in the *P*1̄ space group allows for spatial resolution of the Py and COOH moieties, with their occupancies supporting the ∼1 : 1 An^Py^ : An^COOH^ stoichiometry. H-bonding is observable between the Py and COOH moieties of neighbouring Ans, which feature a 2.593 Å N–O distance, consistent with the majority of aromatic carboxylic acid-pyridine H-bonds reported in the Cambridge Structural Database (Table 1, Fig. S29). This H-bonding interaction also site-differentiates the carboxylic acid O-atoms, allowing for clear resolution of the C–O bond lengths (1.298(8) Å, C–O(H); 1.186(8) Å, C=O) (Table 1, Fig. S29). The π-stacked Ans are separated by 3.727 Å, well within the 4.2 Å maximum distance for SS [4 + 4]-cycloaddition (Table 1).^56^

**Table 1:** Crystallographic parameters of 1 and 1-Dimer. See Table S2 for complete crystallographic data

	1	1-Dimer
CCDC accession code	2595328	2595327
Chemical formula	C_34_H_23_NO_2_	C_34_H_23_NO_2_
Space group	*P*1	*P*1
*a* (Å)	7.9351(3)	7.9110(3)
*b* (Å)	7.9780(3)	8.9083(4)
*c* (Å)	9.9791(2)	9.1741(3)
*α* (°)	81.079(2)	85.215(3)
*β* (°)	88.750(2)	77.334(3)
*γ* (°)	70.177(3)	66.310(4)
*V* (Å^3^)	586.79(4)	577.63(4)
C^9^–C^10’^ (Å)	3.707, 3.741	1.614(8), 1.632(7)
C=O (Å)^a^	1.186(8)	1.23(2)
C–OH (Å)^a^	1.298(8)	1.28(2)
OH⋯N (Å)^a^	2.593	2.594

a Bond metrics determined using solutions in the *P*1̄ space group. See Table S3 for crystallographic data pertaining to this structure solution.

Despite providing information on stoichiometry, bond metrics, and intermolecular interactions, the packing motif of this structure suggests possible functional group disorder that is unresolvable in this space group. The spacing between functional groups prohibits two Py moieties from facing one another, necessitating each Py to be paired with a COOH group (Fig. S29); however, the π-stacked An side of each An^Py^ or An^COOH^ molecule can pair with either the opposite An or another copy of the same An (Fig. 2b and c). This disorder in 1 can be crystallographically modeled by re-solving the structure in the *P*1 space group. We note that in this solution, the O-atoms of the carboxylic acid and 3,5 C-atoms of the pyridine moiety were constrained to occupy the same positions, distorting the bond metrics relevant to the pendant moieties. In the *P*1 solution, there are two distinct C–C contact distances between the 9 and 10-position C-atoms (3.707 and 3.741 Å, respectively, Table 1) which again indicate that this material should undergo photocycloaddition (*vide infra*). In this lower-symmetry model, we find mixed occupancy of the Py and COOH substituent on each side, resulting in a 54.8 : 22.6 : 22.6 ratio of the An^Py^/An^COOH^ π-stacked combination with respect to packing defects An^Py^/An^Py^ and An^COOH^/An^COOH^ in the particular crystal analysed (Fig. 2c, S24).

**Fig. 2:**
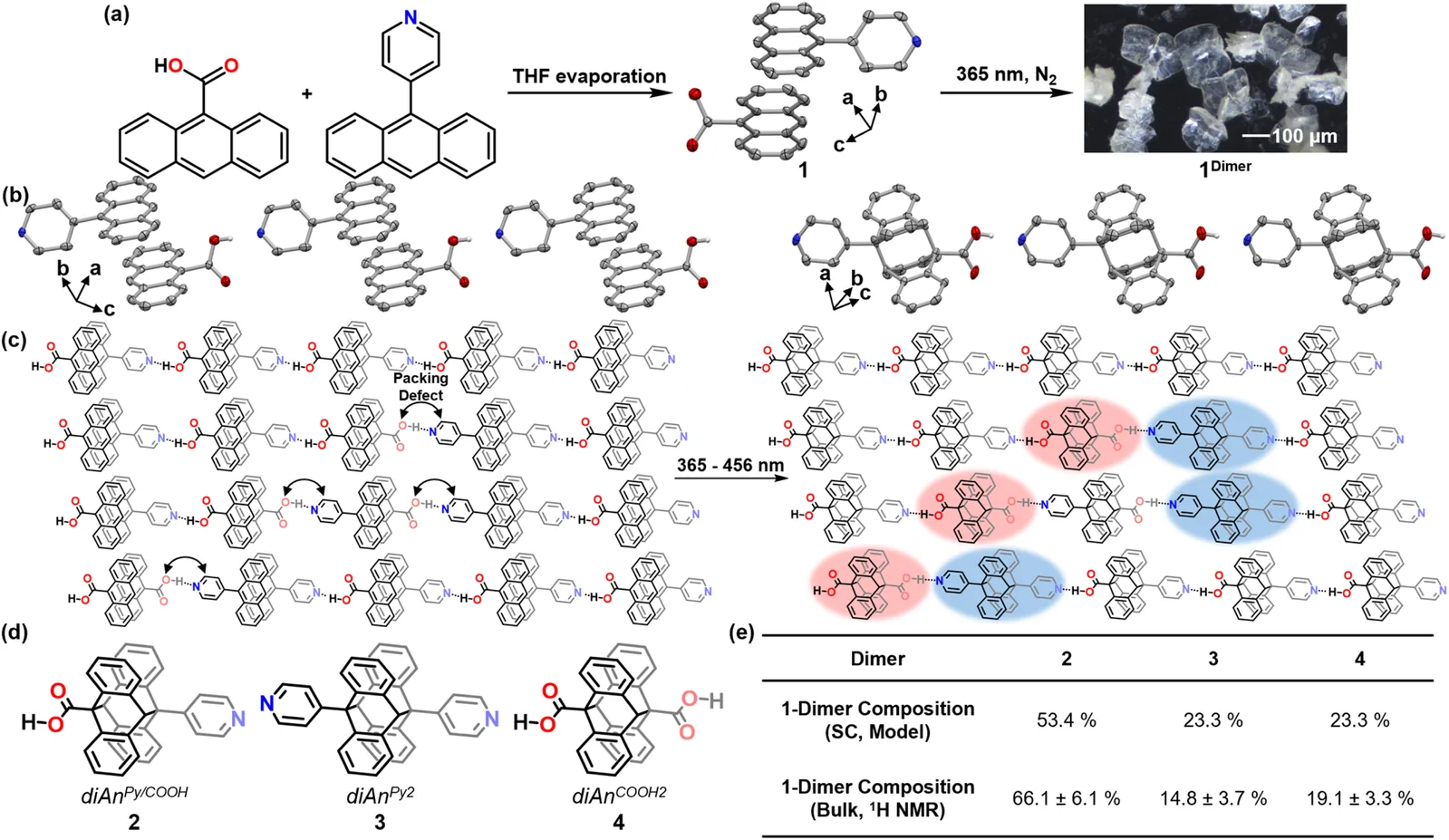
(a) Solid-state synthesis of 1-Dimer *via* the SC–SC transformation of the An^Py^/An^COOH^ cocrystal 1, represented as the major species in the disorder model. (b) 1D chains of 1 (left) and 1-Dimer solved in *P*1 (right) shown as the major species in the disorder model. See SI File and Fig. SX and SX for fully refined disorder models. (c) Crystal packing of 1, demonstrating possible packing defects, and of 1-Dimer, highlighting the possible orientations of 2, 3, and 4 in the structure (d) Molecular structures of 2, 3, and 4. (e) Relative compositions of 2, 3, and 4 present in single and polycrystalline samples of 1-Dimer.

Exposure of crystals of 1 to 365 nm UV light for 3 h under a N_2_ atmosphere results in a colour change from pale yellow to colourless/beige, indicative of the broken aromaticity expected upon formation of a diAn. SCXRD of an irradiated crystal reveals this to be a single-crystal to single-crystal (SC–SC) transformation, as the crystals remain of suitable quality for structural determination after conversion (Fig. 2a, S25, and S30, and Tables 1 and S2). Refinement of the diffraction data was again performed in both *P*1̄ and *P*1 space groups to determine bond metrics and functional group disorder parameters. Both solutions reveal the irradiated material to be the product of [4 + 4]-photocycloaddition, 1-Dimer (Fig. S25 and S30). Little change aside from 9–10′ and 9′–10 C–C bond formation and typical bent “butterfly” diAn shape is observed, with the structure comprising tightly-packed 1-D H-bonded chains (N–O distance = 2.954 Å, *P*1̄) in the same motif as the unlinked precursor (Table 1, Fig. 2b,c and S27). The 9,9′-substituents remain disordered, best modelled for the particular crystal run as a 53.4 : 23.3 : 23.3 distribution of the heterodimeric 9-pyridyl dianthracene-9′-carboxylic acid (diAn^Py/COOH^, 2) and the two homodimers, 9,9′-dipyridyl dianthracene (diAnPy, 3) and 9,9′-dianthracene dicarboxylic acid (diAn^COOH^, 4) (*P*1, Fig. 2d and e). We note that different individual crystals were used for SCXRD of 1 and 1-Dimer (Fig. S43 and S44), despite being derived from the same batch of crystals. The differences in disorder population modelled for 1 (54.8% π-stacked An^Py^/An^COOH^ pairs) and 1-Dimer (53.4% heterodimer 2) reflect slight variations in the number of packing defects between each crystal formed during the synthesis of 1, which are then preserved in 1-Dimer. ^1^H NMR spectra of bulk samples of 1-Dimer produced by irradiation of crystals of 1 confirm that the bulk material is comprised of all three dimers, 2–4 (Fig. S7), with heterodimer 2 as the major species in all cases.

The P1 SCXRD disorder models for both 1 and 1-Dimer indicate that there is one preferential packing direction for the An^Py^/An^COOH^ π-stack in 1 and thus for the heterodimer 2 in 1-Dimer, with the opposite orientation (*i.e.* “An^COOH^/An^Py^” and “diAn^COOH/Py^”) accounting for just 12.0% and 13.6% in the single crystals of 1 and 1-Dimer analysed. An isolated instance of an An^COOH^/An^COOH^ or An^Py^/An^Py^ packing defect in 1 would lead to many copies of the “flipped” orientation of 2 in 1-Dimer (Fig. 2b). However, the low proportion of this reversed orientation in the SCXRD of both 1 and 1-Dimer indicates that most packing defects in 1 occur as neighbouring An^COOH^/An^COOH^ and An^Py^/An^Py^ pairs (Fig. 2c).

Neither the *P*1 nor the *P*1̄ solution of 1-Dimer feature any disorder related to unreacted 1 (Fig. S27). However, irradiation of a bulk sample of SCXRD-quality crystals, which includes heterogenous crystals sizes and clumps of material, results in partial conversion from 1 to 1-Dimer by ^1^H NMR. Size-dependent SC–SC transformations have been previously reported, wherein large crystals of pristine material undergo deformation and/or fracture upon reaction, while smaller micro/nanocrystals retain crystallinity due to reduced internal strain.^67,68^ While this could be operative here, the crystal showing complete conversion to 1-Dimer by SCXRD was retrieved from the bulk partially-converted crop of 1/1-Dimer crystals, indicating that the process is also likely limited by light scattering in thick material samples (Fig. S3).

To achieve bulk conversion to 1-Dimer, an equimolar solution of An^Py^ and An^COOH^ was evaporated in a Pyrex dish at room temperature, swirling the mixture to form a surface coated with 1 with average microcrystal sizes of 6.8 × 10^4^ µm^3^ and coating thicknesses of 70–480 µm (Fig. S47). Powder X-ray diffraction (PXRD) of the microcrystals shows that this method forms 1 as a phase-pure product (Fig. S31). Irradiation of the solids under an N_2_ atmosphere results in a colour change from yellow to beige, and PXRD confirms the material to be converted to 1-Dimer (Fig. S31, S45, and S46). A N_2_ atmosphere is needed to prevent the formation of anthraquinone from An^COOH^ irradiated under air.^9,69^ The ^1^H NMR spectrum of the irradiated powder shows >90% conversion to 1-Dimer (Fig. S5). Across multiple syntheses, the average proportion of diAns in 1-Dimer is 66 ± 6% heterodimer 2, 15 ± 4% Py homodimer 3, and 19 ± 3% carboxylic acid homodimer 4 (Fig. 2e). We note that, due to the poor solubility of 2 and 3, the relative composition of 4 is likely slightly overestimated in all ^1^H NMR studies of 1-Dimer in this report. These populations are broadly consistent with the populations determined by disorder modelling of the SCXRD structures of 1 and 1-Dimer, suggesting a reproducible distribution of hetero- and homodimers driven by monomer packing in 1 (Fig. 2e).

### Energy storage in diAns

We assessed the energy storage capacity of 1-Dimer and isolated, recrystallized samples of its components 2–4 using differential scanning calorimetry (DSC). The DSC trace of 1-Dimer (collected at 5 °C min^−1^) is featureless until 145 °C, where the onset of a broad exothermic event with a Gaussian line shape is observed until a plateau at 180 °C (Fig. 3a). This is consistent with heat release upon retrocyclization of the 9,10’/10,9′ bonds of the component dimers (*T*^retro^). The melting point of the retrocyclized material (*T*^melt^) is 207 °C, as evidenced by the endothermic event in the DSC trace (Fig. 3a). We note that 1-Dimer displays one broad retrocyclization exotherm despite being formally a mixture of three species with distinct *T*^retro^, and that this event was observed to be scan rate dependent in both onset temperature and integration (*vide infra*, Fig. S39). The broad line shape could be a consequence of the disordered nature of 1-Dimer crystals as it is observed for both the retrocyclization and melting features. It is also possible that the event is broadened because samples of 1-Dimer consist of an ensemble of 1-Dimer microcrystals with subtly varied ratios of 2, 3, and 4. The large difference between *T*^retro^ and *T*^melt^ (Δ*T*^m–r^, 62 °C) indicates that the retrocyclized phase 1 is stable at high temperature and suggests that 1/1-Dimer is well-suited for SS cycling (Fig. 3a, *vide infra*).

**Fig. 3:**
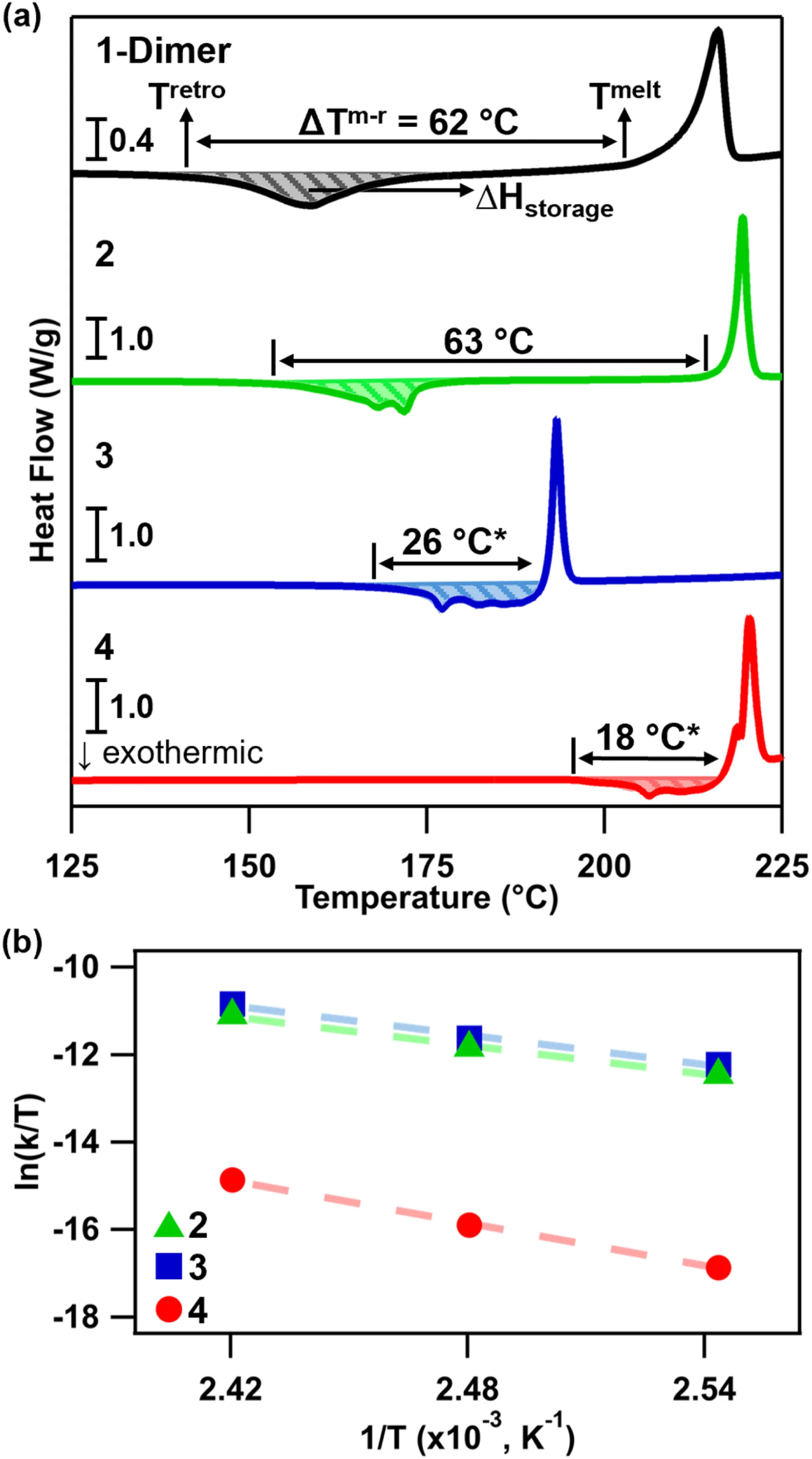
Energy storage in 1-Dimer, 2, 3, and 4. (a) DSC traces of each species collected at 5 °C min^−1^, highlighting *T*^retro^, *T*^melt^, *T*^m−r^, and Δ*H*_storage_. See Fig. S30 for DSC traces of 1-Dimer collected at 2, 3.5, and 5 °C min^−1^. *Determination of *T*^m−r^ and Δ*H*_storage_ for 3 and 4 are convoluted by overlap between the retrocyclization and melting events. (b) Eyring plots for retrocyclization of 2, 3, and 4 in DMSO-d_6_.

Separation of the constituent dimers from 1-Dimer is possible by column chromatography. Independent synthesis of each dimer is also possible by irradiation of the appropriate Ans in THF solution, with chromatography required for isolation of diAn^Py/COOH^ (2, see Experimental section for further details). The DSC traces (collected at 5 °C min^−1^) of the pure homodimers diAn^Py2^ (3) and diAn^COOH2^ (4) feature *T*^retro^ exotherms with onset temperatures at 165 °C and 196 °C, respectively (Fig. 3a). Notably, these events overlap with the melting points of their respective monomers. On the other hand, the DSC curve of the purified heterodimer 2 features a *T*^retro^ of 151 °C and Δ*T*^m–r^ of 63 °C, similar to that of 1-Dimer (Fig. 3a). All exotherms for the purified diAns feature asymmetric line shapes, indicating potential phase changes along the retrocyclization pathway (*vide infra*).^51,70^

Integration of the retrocyclization events allows us to calculate the energy storage capacity of each species, expressed as the retrocyclization enthalpy, Δ*H*_storage_. Because *T*^retro^ falls in close proximity to *T*^melt^ for 3 and 4 and their corresponding monomers, the measured energy storage capacity is lower than the true Δ*H*_storage_, though convention is to report this measured value as Δ*H*_storage_. The measured capacities of 3 and 4 are 55.0 J g^−1^ and 57.8 J g^−1^, respectively (Table 2). 2, on the other hand, has a capacity of 101.3 J g^−1^, as the high Δ*T*^m–r^ means that all of the energy released upon retrocyclization is accessible without losses due to melting (Fig. 3a, Table 2). 1-Dimer has an energy storage capacity of 81.6 J g^−1^, slightly lower than the purified material; however, this value is scan rate dependent, with slower scan rates (*i.e.* 3.5 and 2 °C min^−1^) finding higher Δ*H*_storage_ values for 1-Dimer (85.9 and 103.8 J g^−1^, respectively, Fig. 3a and S39, Table 2). This allows for improved resolution of the broad exotherm, which we posit is a consequence of this inherently disordered material. Though these energy capacities are lower than those reported for other diAns, the simple components and large Δ*T*^m–r^ of 1-Dimer make it an attractive active species for cycling.

**Table 2:** Energy storage parameters of dianthracenes

Compound	*T* ^retro^ (°C)	Δ*T*^m–r^ (°C)	Δ*H*_storage_ (J g^−1^)^a^	*t*_1/2_ in solution (years)^c^
1-Dimer	145	62	81.6	—
2	151	63	101.3	0.154
3	165	26^b^	55.0^b^	0.146
4	196	18^b^	57.8^b^	833

a Δ*H*_storage_ determined at 5 °C min^−1^. This was found to be scan rate-dependent in analyses of 1-Dimer. See Fig. S30 for all Δ*H*_storage_ values.

b Δ*T*^m–r^ and Δ*H*_storage_ for 3 and 4 are convoluted by coincident retrocyclization and melting processes.

c Calculated in DMSO solution at 25 °C.

Variable-temperature retrocyclization kinetics experiments performed in DMSO-*d*_6_ solution and monitored by ^1^H NMR allow us to calculate the solution retrocyclization activation barriers and storage half-lives of each isolated dimer. Eyring analysis of the data shows that 4 has the highest Gibbs free energy of activation at all temperatures (Δ*G*^‡ retro^) of the series (Table 2, Fig. S48–53). 2 and 3 feature nearly identical Δ*G*^‡retro^. The lower activation energy of the two pyridyl-containing species corresponds to a significant reduction in solution-state storage half-lives at 25 °C (approximately 0.15 years) *versus* the dicarboxylate species (833 years, Table 2).

To understand the structural basis for these solution-state observations, we turned to density functional theory (DFT) calculations. Optimized gas-phase molecular structures for each dimer species were calculated using the B3LYP functional and dev2-SVP basis set (see SI files for optimized structures). The expected mechanism of retrocyclization is cleavage of one C–C bond to form a diradical.^70,71^ The most stable of the three materials, 4, features the shortest computed central C–C bond distances (1.622 Å). On the other hand, 2 and 3 feature similar central C–C bond lengths (1.634/1.635 and 1.638 Å, respectively). These observations are in agreement with the kinetics data, with 4 requiring the greatest energy input for retrocyclization and both pyridyl-functionalized materials, 2 and 3 having similar bond lengths and activation parameters. We hypothesize that the size of the pyridyl moiety sterically strains the dimer core, weakening the key C–C bonds in 2 and 3 compared to in 4.

The trends in the experimental solution retrocyclization barriers and calculated gas-phase structures of 2–4 are self-consistent but do not translate well to the SS retrocyclization parameters. Given the relevance of the central C–C bond lengths and angles and the volume change required for retrocyclization, crystal packing is well known to affect the retrocyclization behaviour of diAns. *T*^retro^, derived from DSC, should be correlated with the SS retrocyclization activation energy, such that the relative ordering of *T*^retro^ (4 > 3 > 2 > 1-Dimer) is also the relative ordering of the SS Δ*G*^‡retro^.^70^ Comparing this parameter across 2, 3, and 4, we again find that the dicarboxylic acid species has the highest energetic requirement (*T*^retro^ = 196 °C, Table 2). However, 3 features a significantly higher *T*^retro^ than the isolated heterodimer 2 (165 *vs.* 151 °C, respectively), indicating that the dipyridyl compound is significantly more stable in the solid state, while the activation energy in solution was nearly identical for both compounds. The SS synthesized 1-Dimer, despite being formally a mixture of 2–4, has the lowest apparent activation barrier (*T*^retro^ = 145 °C).

These inconsistencies suggest that influences beyond functional group sterics and electronics play a substantial role in the stability of diAns in the solid state. For instance, changes in crystal packing between the dimer and corresponding monomers may impact the retrocyclization barrier.^70^ In the case of 1-Dimer, the crystal lattice/packing is dictated by the initial structure of 1. This structure is a favourable solid-state packing arrangement for the planar monomers, as demonstrated by the fact that samples of pure heterodimer 2 packed in a different crystalline lattice reorganize to crystalline 1 upon annealing (*vide infra*). However, upon cycloaddition, the metastable dimers take on a “butterfly” shape. Despite the retention of crystallinity, this may generate internal strain due to crystalline confinement, causing the SC–SC–synthesized 1-Dimer to have a lower *T*^retro^.^70^ On the other hand, the DSC samples of 2–4 were isolated and precipitated from solution as dimers, likely producing a more energetically favourable SS packing for these compounds as individual species than for 1-Dimer. To rephrase more simply, our interpretation is that 1-Dimer represents a metastable SS form of the component dimers, 2–4, which are therefore more strained in 1-Dimer than in the DSC samples of 2–4 that are independently precipitated from solution. We propose that this leads to the lower observed *T*^retro^ for 1-Dimer compared to isolated 2.

To investigate the photocycloaddition (*i.e.* “charging”) process under application-relevant conditions, we studied the formation of 1-Dimer using simulated sunlight and visible light-emitting LEDs. Of the three preceding examples of diAn-mediated MOST energy storage, one demonstrates efficient conversion of the An active species in dilute solution using unfiltered sunlight, with the two SS examples opting for irradiation using UV (370 nm) or violet (405 nm) light. Irradiation of a surface-cast sample of 1 using a solar simulator produces 88% conversion to 1-Dimer in 9 h, and ∼90% conversion over 12 h with 1 sun intensity (Fig. S8). The diffuse reflectance spectrum (DRS) of 1 suggests that visible light exposure, not only the UV component of sunlight, could charge the material to 1-Dimer (Fig. S40), as 1-Dimer shows significant absorbance between 300 and 440 nm. To assess this, violet (427 nm) and blue (456 nm) LED lamps were shone on a solid sample of 1. Within 6 h, exposure to either lamp resulted in near quantitative conversion (>95%) to 1-Dimer (Fig. S9 and S10). We note the intensity of these lamps is greater at their wavelengths than that emitted by the solar simulator, decreasing the charging time (Table S1). This is a rare demonstration of diAn formation using visible light, which typically requires highly specialized Ans with complex functionalities and extended π-systems to bathochromically shift the relevant absorption bands.^72–74^ Such functionalization schemes would be unfavourable for MOST platforms, as the added complexity would increase cost and higher mass lowers the energy storage density. In this case, the absorbance profile of 1 is not substantially shifted to higher wavelengths, particularly compared to An^COOH^ (Fig. S40) and previous π-stacked anthracene MOST systems from the Han and Hutchins groups.^9,50^ Instead, we simply find that 1 shows a remarkable ability to convert to 1-Dimer even under irradiation at the tail of its absorbance profile.

### Cycling performance

A critical performance metric of any energy storage platform, including MOST systems, is the ability to stably undergo charge–discharge (C–D) cycling. Only a few MOST systems such as the styrylpyrylium and azobenzene-decorated adamantane platforms by the Han group have been shown to be fully cyclable in the solid state for more than 2 cycles.^6,75^ The crystal-to-crystal nature, high conversion and DSC of the 1/1-Dimer system all indicate that solid state cycling should be possible. C–D analyses over 12 cycles were performed using *ex situ*^1^H NMR spectroscopy on aliquots of the solid removed after each charging (365 nm irradiation) and discharging (thermal annealing) step. The state of charge (SOC) of the sample was determined using the relative population of 2, 3, and 4 (the chemical components of 1-Dimer) compared to 1 (An^Py^ and An^COOH^) in the spectrum recorded after each step (Fig. 4b, S15, and S16).

**Fig. 4:**
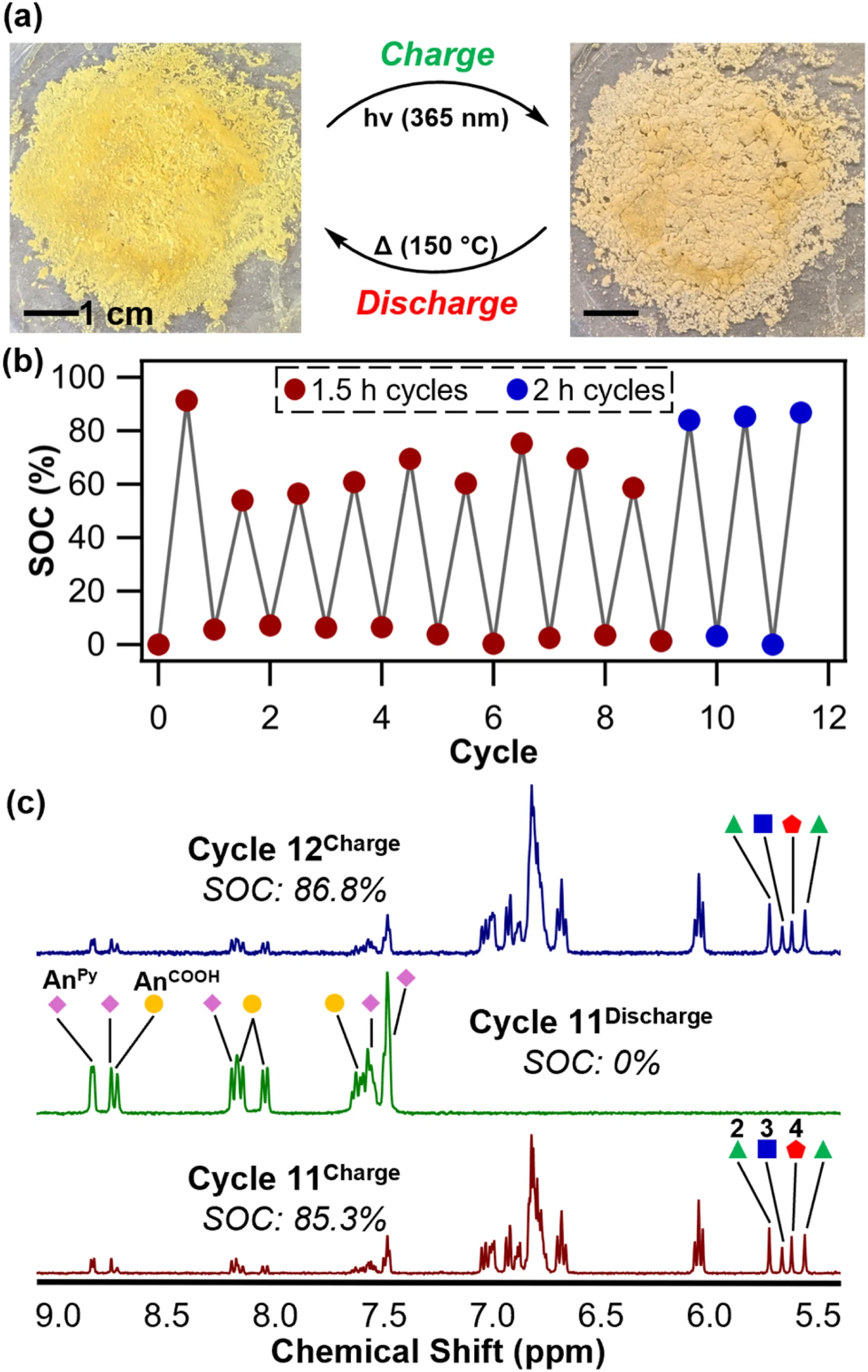
(a) C–D cycling of 1. (b) C–D profile over 12 cycles. (c) ^1^H NMR data recorded during the 11th and 12th C–D cycles.

The monomer mixture was cast onto a Pyrex dish to form microcrystals of 1, and a ^1^H NMR spectrum was recorded prior to the first charging cycle. Initial charging of the material for 90 min achieved 91.4% SOC (Fig. 4b and S15). All charging cycles were performed under a N_2_ atmosphere to prohibit conversion to anthraquinone.^9,69^ The sample was then discharged at 150 °C in an oven in air for 90 min, resulting in cycloreversion to 5.7% SOC (see SI file for details). The material was cycled for 90 min C–D intervals for 9 cycles, achieving an average charging SOC of 66 ± 11% and discharging to 4.5 ± 2.2% SOC (Fig. 4b and S15). The final 3 cycles were conducted with 120 min C-D intervals, achieving 85.4 ± 1.1% and 1.5 ± 1.3% SOC, respectively (Fig. 4b, c, and S15). Visible light C-D cycling was additionally performed with an identical setup using 456 nm irradiation, which revealed similar stability and faster charging rates due to the higher irradiance afforded by the LED light source (Fig. S17 and S18, Table S1). We note that no decomposition or anthraquinone formation was observed in either C–D experiment, highlighting the stability of this material under cycling conditions. After the 12th cycle, insufficient solids remained in the sample to conduct further analyses; however, the lack of byproducts and high SOC observed in the final 3 charging intervals suggests that this system could undergo further C–D cycling.

We hypothesize that the stable cycling performance of 1/1-Dimer is due to the relatively small changes in the crystal structure between the monomer and dimer forms, as well as the thermodynamic stability of the monomer structure (*vide supra*). In fact, the unit cell volumes between these two isomers are nearly identical, differing by only 1.6% (Table 1). To assess the impact of the H-bonded chain structure of 1 on cycling performance, we conducted analogous C–D experiments with the isolated dimers 2, 3, and 4. In these cases, the same SS setup and cycling conditions were employed. Initial discharging of all 3 dimer species produce the corresponding monomers; however, the subsequent charging step yields vastly different results (Fig. S19–S21). Attempted recharging of 3 yields no dimer after 180 min of irradiation. 4 does reform upon irradiation in the solid state; however, only 10.3% SOC is reached in 90 min, and the material is only charged to 31.1% after 24 h. After retrocyclization, 2, which is initially the pure heterodimer, is shown to recharge to 53.6% under LWUV after 90 min. However, the resultant ^1^H NMR spectrum reveals that the diAn products of the second charge cycle are indeed a mixture of all three dimers present in the mixed dimer species, 1-Dimer (Fig. S21). The relative dimer populations in this second charging step mirror those found in the SC–SC synthesis and C–D cycling of 1 (68% 2, 14% 3, 18% 4), further indicating a thermodynamic preference for the monomer lattice with a consistent ∼30–40% disorder (*vide supra*). As expected from this observation, PXRD of annealed samples of 2 are consistent with the formation of 1 upon retrocyclization at high temperatures (Fig. S35). Thus, despite the higher energy storage capacity of 2, its thermal cycloreversion to 1 prohibits the purified heterodimer from cycling. The rearrangement of 2 to disordered 1 upon retrocyclization is consistent with the DSC line shape of the retrocyclization event for 2, which features several peaks rather than the continuous, Gaussian shape expected if retrocyclization occurs without additional lattice processes. Such asymmetric DSC features suggest overlapping exothermic and endothermic responses which have been attributed to reaction intermediates/structural rearrangements along the diAn retrocyclization process.^51,70,76^

### Electron beam retrocyclization of 1-Dimer

The crystal-to-crystal nature of 1/1-Dimer cycling prompted us to investigate whether 1-Dimer retrocyclization could be achieved and tracked by e-beam irradiation *via* MicroED. Jones reported parent diAn retrocyclization under an e-beam in 1976; the diffraction pattern of unfunctionalized, parent diAn changed to that of parent An in a transmission electron microscope (TEM), indicating this to be a crystal-to-crystal e-beam driven process.^53^ Despite this intriguing result, no other e-beam driven diAn retrocyclizations have been demonstrated to date. The increasing availability of MicroED has brought increased awareness, attention, and structural resolution to crystal-to-crystal processes triggered by e-beam exposure. García-Garibay and coworkers have recently demonstrated crystal-to-crystal e-beam driven retrocyclization of cyclobutanes, including tetrakis(4-pyridyl) cyclobutane and the thymine dimer, using MicroED.^54,55^ In both cases, diffraction patterns of the metastable [2 + 2] photoaddition products were observed to evolve with e-beam dose, eventually producing their rectrocyclized alkene forms. These reports, coupled with our observations on the 1/1-Dimer system, suggested that MicroED may provide the opportunity to observe and track a new e-beam driven diAn retrocyclization.

A single data collection on each of three crystals of 1-Dimer, sweeping between −50–50° tilt angles with a total e-beam dose of 0.662 e^−^ Å^−2^ at room temperature reflects unit cell parameters consistent with the 1D, H-bonded dimer lattice of 1-Dimer (Fig. 5, Tables S4–S6). However, subsequent data collections on the same crystals reveal gradual shifts in the cell dimensions, indicative of retrocyclization. After 14–20 runs (9.268–13.240 e^−^ Å^−2^ dose), the unit cells are all consistent with that of retrocyclized 1 (Fig. 5, Tables S4–S6). Refinement of the merged data enables us to resolve the structure of the product of e-beam irradiation. We note that, as is typical, the MicroED structure lacks the resolution typically achieved by XRD;^77^ however, the structure does reveal cleavage of the 9–10′ C–C bonds, planarity of the An rings, and overall consistent packing with the SCXRD structure of 1 (Fig. S37 and S38). The unit cell progression and resolved MicroED structure indicate that e-beam exposure directly converts 1-Dimer back to the π-stacked, H-bonded lattice of 1 in a crystal-to-crystal fashion, adding a second example of e-beam driven diAn retrocyclization to the literature and pointing to a possible generality of e-beam driven retrocyclizations of metastable photocycloaddition products.

**Fig. 5:**
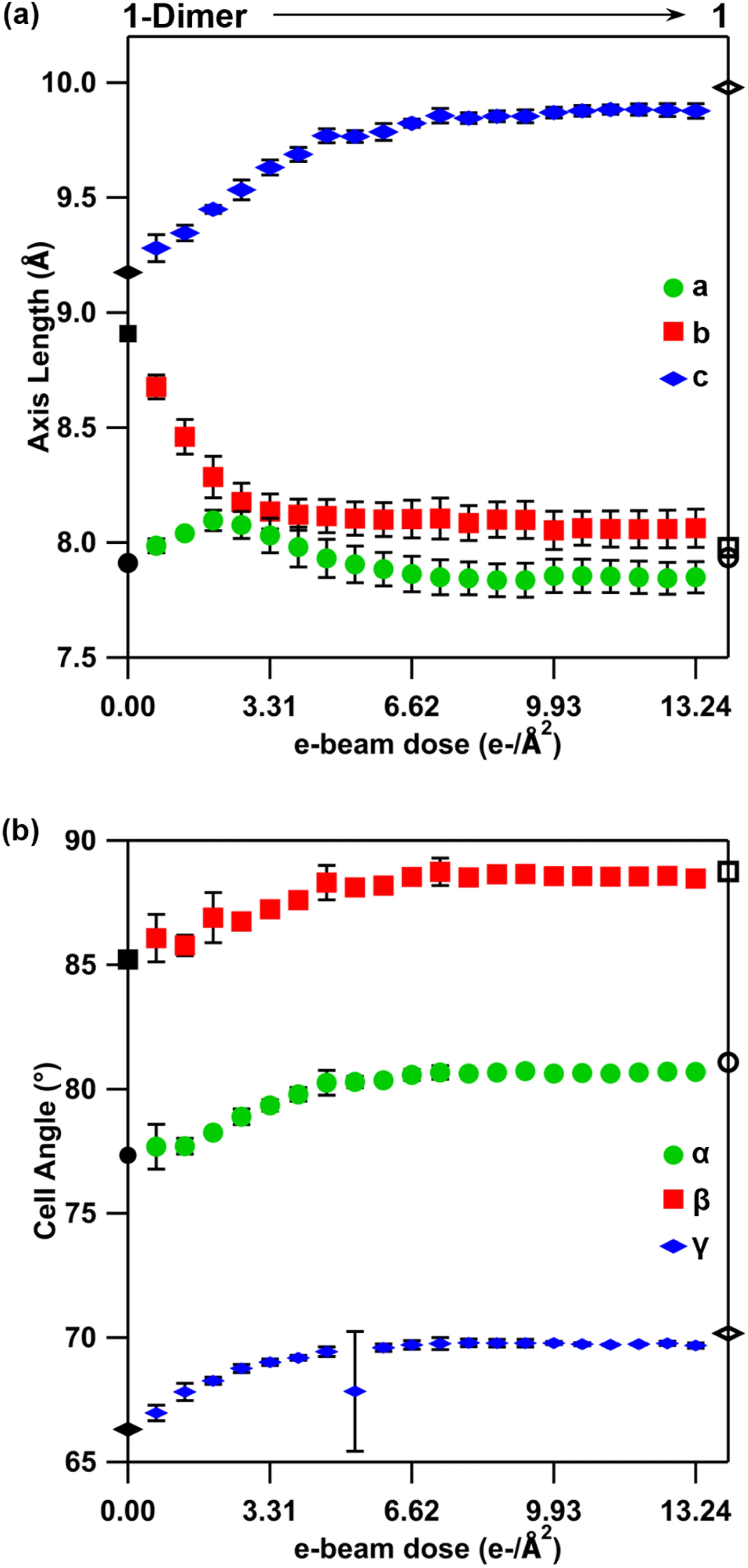
Evolution of unit cell (a) axes lengths and (b) angles of crystals of 1-Dimer determined using MicroED as a function of e-beam dose at 102 K. MicroED-derived parameters are an average of 3 crystals from 0.662–9.268 e^−^ Å^−2^, then an average of 2 crystals from 9.930–13.240 e^−^ Å^−2^. Solid black and hollow black markers denote the parameters for 1-Dimer and 1 determined by SCXRD collected at 100 K.

## Conclusions

The development of design principles for sunlight-chargeable, SS MOST battery materials is crucial to reaching practical implementation of this technology. Anthracenes are attractive candidates owing to their well-defined, reversible [4 + 4] photocycloaddition reactivity and readily accessible functionalization chemistry. Despite this, few reports of MOST systems based on An/diAn have been published to date, with none employing heterodimeric species. By combining An^Py^ and An^COOH^, we invoke H-bonding as a simple, direct means of prearranging An monomers to improve SS photocyclization efficiency.^9^ Upon LWUV, simulated sunlight, or blue light exposure the resultant H-bonded, π-stacked structure (1) forms 1-Dimer, a 1D H-bonded lattice comprising the heterodimer 2 and two homodimers, 3 and 4. This mixture features comparable energy storage capacity (103.8 J g^−1^) to the isolated heterodimer (101.3 J g^−1^) and a reduced SS activation temperature *versus* each of its component parts. The monomer prearrangement additionally enables this material to stably cycle between its charged (1-Dimer) and discharged (1) states without manipulation between cycles. This study presents further evidence that An/diAn materials are a privileged class for all SS cyclable MOST owing to the small volume change of their photocycloaddition/retrocyclization and demonstrates how intermolecular forces can be leveraged to improve SS photon energy storage performance. MicroED experiments revealed that the crystal-to-crystal retrocyclization of 1-Dimer to 1 can also be driven by e-beam exposure, marking just the second example of e-beam driven [4 + 4] retrocyclization.

## Author contributions

E. S. and A. K. B. conceived and planned the experiments. E. S. synthesized all compounds and collected all data. H. S. assisted with cycling experiments. M. E. L. performed the MicroED studies. M. Y. P., M. E. L., and C. K. assisted in the isolation of dimer species. A. K. Banki performed DFT calculations. E. S. wrote the draft of the manuscript. E. S. and A. K. B. edited and revised the manuscript.

## Conflicts of interest

There are no conflicts to declare.

## Supplementary Material

SC-OLF-D6SC05227D-s001

SC-OLF-D6SC05227D-s002

SC-OLF-D6SC05227D-s003

SC-OLF-D6SC05227D-s004

## Data Availability

CCDC 2563067–2563070, 2594106, 2595327 and 2595328 contain the supplementary crystallographic data for this paper.^78*a–g*^ Supplementary information (SI): detailed experimental and synthetic methods and characterization, including NMR, DSC, optical images, XRD, MicroED, crystallographic tables, ESI-MS, and DRS. See DOI: https://doi.org/10.1039/d6sc05227d.
