# Supplementary material for: Crystal-to-crystal solar energy storage in a hydrogen bonded dianthracene lattice retrocyclized by heat or e-beam
Source: Chem Sci. 2026 Oct 23. Online ahead of print. doi: 10.1039/d6sc05227d (PMC13718465; doi:10.1039/d6sc05227d)
Supplement: Supplementary file 1 [file SC-OLF-D6SC05227D-s004.pdf]

Supplementary Information (SI) for Chemical Science.
This journal is © The Royal Society of Chemistry

Supplementary Materials for

Crystal-to-crystal solar energy storage in a hydrogen bonded dianthracene lattice retrocyclized by heat or e-beam

*Eric Schreiber; Hovnan Simonyan; Madison E. Logelin; Aleksa Banki; Madeleine Y. Popofsky; Christopher Kim; Amymarie K. Bartholomew**

Department of Chemistry, Yale University, New Haven, Connecticut 06520, United States

E-mail: amymarie.bartholomew@yale.edu

Table of Contents:

A. Experimental Section....S2
B. NMR Spectroscopy....S7
C. Electrospray Ionization Mass Spectrometry....S22
D. X-Ray Diffraction and Refinements....S23
E. Microcrystal Electron Diffraction....S35
F. Differential Scanning Calorimetry....S38
G. Diffuse Reflectance Spectroscopy....S39
H. Microscopy....S40
I. Retrocyclization Kinetics....S44
J. References....S50

A. Experimental Section

General Considerations: All reactions and manipulations were performed under ambient conditions in air unless otherwise noted. All commercially available materials were used as received (9-anthracenecarboxylic acid (**An**^{COOH}): Alfa Aesar; 4-pyridinylboronic acid, cesium carbonate: AmBeed 9-bromoanthracene, tetrakis(triphenyl phosphino) palladium: TCI; . Tetrahydrofuran (THF) was dried and deoxygenated with Ar on a Pure Process Systems solvent purification system.

Light Sources: Long-wave UV light irradiation (LWUV, 365 nm) was performed using an Analytik Jena UVP Blak-Ray 365 nm lamp with the Visible and IR filter removed. 427 nm and 456 nm irradiation was performed using Kessil PR160L Gen 2 LED lamps set to maximum power. Simulated sunlight irradiation was performed using a VeraSol Solar Simulator set to 1 sun intensity. Irradiance for all light sources was measured using a Coherent FieldMate Laser Power Meter with a Coherent PowerMax PM10V1 sensor with a detector area of 2.84 cm^2. Power was measured at the distance the sample was irradiated at during irradiation experiments and converted to irradiance by converting to Watts and dividing by the detector area in m^2. Values are listed in Table S1.

Table S1. Irradiance values for the light sources used in irradiation reactions.

Source Wavelength	365 nm	427 nm	456 nm	Solar simulator
Irradiance (W/m^2)	314,000	110,000	126,000	25,900

Nuclear Magnetic Resonance (NMR) Spectroscopy: ^{1}H NMR data were recorded on an Agilent DD2 400 MHz spectrometer. ^{13}C{^{1}H} NMR data were recorded on an Agilent DD2 600 MHz spectrometer. All resonances in the ^{1}H and ^{13}C NMR spectra are referenced to residual proteo dimethyl sulfoxide (DMSO, δ 2.50 ppm) or dichloromethane (DCM, δ 5.32 ppm).

Electrospray Ionization Mass Spectrometry (ESI-MS): ESI-MS was recorded on a Shimadzu Scientific Instruments QToF 9030 LC-MS system, equipped with a Nexera LC-40D xs UHPLC, consisting of a CBM-40 Lite system controller, a DGU-405 Degasser Unit, two LC-40D XS UHPLC pumps, a SIL-40C XS autosampler and a Column Oven CTO-40S in the negative mode.

Microscopy: Images of crystals of **1** and **1-Dimer** were collected using a Nikon ECLIPSE LV150N microscope fitted with a LV-UEPISCOPIC-N-CH universal episcopic illuminator and DS-100 digital sight microscope

camera and processed in the Nikon NIS-Elements software suite. Images of powder samples were taken using a Google Pixel 9 smartphone and calibrated using a cm ruler.

Powder X-Ray Diffraction (PXRD): Powder diffraction patterns were collected on a Rigaku MiniFlex600 Powder X-ray Diffractometer with Cu Kα (λ = 1.5418 Å) radiation at room temperature.

Single Crystal X-Ray Diffraction (SCXRD): Single crystal X-ray structures of **An$^{Py/COOH}$**, **1**, and **3** were collected on a Rigaku XtaLAB Synergy, Dualflex diffractometer coupled to a HyPix-Arc 100 detector with Cu Kα (λ = 1.54184 Å) at 100.01(11) K, 293(2), and 293(2) K, respectively. The structure of **2** was collected on a Rigaku 007 HF+ diffractometer coupled to a Pilatus3R 200KA detector with Mo Kα (λ = 0.7107 Å). The diffraction images were processed and scaled using Rigaku Oxford Diffraction software (CrysAlisPro; Rigaku OD: The Woodlands, TX, 2015). Using Olex2.1,[1] the structure was solved with the SHELXT[2] structure solution programing using Intrinsic Phasing and refined against F2 on all data by full-matrix least squares with SHELXL.[3] All non-hydrogen atoms were refined anisotropically. Hydrogen atoms were included in the model at geometrically calculated positions and refined using a riding model. The isotropic displacement parameters of all hydrogen atoms were fixed to 1.2 times the U value of the atoms to which they are linked. The structure of **1**, **1-Dimer**, **2**, and **3** have been deposited with the CCDC, with deposition numbers 2563067 (**1**), 2563068 (**1-Dimer**), 2563069 (**2**), and 2563070 (**3**). Data can be obtained free of charge from The Cambridge Crystallographic Data Center via http://www.ccdc.cam.ac.uk/conts/retrieving.html

Microcrystal Electron Diffraction (MicroED): Samples were prepared by crushing crystals of **1-Dimer** between two glass slides and depositing the microcrystalline powder onto a 3 mm 200 mesh copper grid with lacey carbon support film (TedPella, Inc.). Data were collected at 102 K on an XtaLAB Synergy-ED electron diffractometer coupled to a HyPix-ED detector with a 200 keV (λ = 0.0251 Å) electron beam. A single tilt series from -50 to 50 degrees were collected for three unique crystals of **1-Dimer**. The data for each crystal were collected as a series of consecutive runs using the same parameters for each data collection: dose rate = 3.31×10^{-3} e/Å^{2}s, exposure time = 2 s/frame, scan width = 0.25°/frame, total dose per run = 6.62×10^{-1} e/Å^2. For each crystal, the unit cells tracked over consecutive data collection show a transformation from **1-Dimer** to **1**. The bulk of the transformation occurs in the first 10 runs after which the unit cells remain consistent with little change in diffraction. The structure of **1** was determined by integrating and merging the datasets of three crystals, one exposed to a total dose of 9.268 e/Å^2 and two exposed to a total dose of 13.24 e/Å^2. Using Olex2,[1] the structure was solved with the SHELXT[2] structure solution program using Intrinsic Phasing and refined with the SHELXL[3] refinement package using Least Squares minimization. To achieve a stable refinement, all thermal parameters were restrained to be similar to one another with a standard uncertainty of 0.002. Hydrogen atoms were included in the model at geometrically calculated positions and refined using a riding model. The isotropic displacement parameters of all hydrogen atoms were fixed to 1.2 times the U value of the atoms to which they are linked. The low data to parameter ration and weak diffraction contributed to the non-ideal least squares refinement. Though the atoms cannot be anisotropically refined, many figures of merit (completion, resolution, goodness-of-fit, R_1) fall within standards recommended by Gonen et al.[4] Additionally, the unit cell parameters and atom connectivity agree with those of the X-ray structures of **1**. The full numbering scheme of the compound **1** can be found in the full details of the structure determination (CIF), which is included as Supporting Information. The

structure of **1** has been deposited with the CCDC, with deposition number 2594106. These data can be obtained free of charge from The Cambridge Crystallographic Data Center via http://www.ccdc.cam.ac.uk/conts/retrieving.html

Differential Scanning Calorimetry (DSC): DSC measurements were performed on a TA Instruments DSC250 instrument. 2-5 mg of sample was loaded in a TA Instruments Tzero Pan and sealed with a Tzero Lid using a press. Temperature was initially cooled to and held at –10 °C for 5 minutes, then ramped from –10 °C to 300 °C at a rate of 5 °C/min.

Computational Methods:

Geometry optimizations were done for **2**, **3**, and **4** using density functional theory (DFT) calculations at the B3LYP/dev2-SVP level of theory with Orca 6.0.0.[5-8] These optimizations were done on modified single-crystal XRD structures in which atoms were correctly assigned and disorder was removed.

Synthetic Methods:

9-pyridylanthracene (**An**^{Py})[9] and 9,9'-dianthracenedicarboxylic acid (diAn^{COOH_2}, **4**)[10] were synthesized using published procedures.

*Synthesis of X-ray quality cocrystals of 9-pyridylanthracene and 9-anthracenecarboxylic acid (**1**).*

An equimolar mixture of **An**^{Py} and **An**^{COOH} (134 mM) in 1.5 mL THF in a 1 dram vial was placed into a 20 mL scintillation vial with 6 mL hexanes. The scintillation vial was sealed and the vapor diffusion proceeded for 4 days, at which point yellow crystals suitable for single crystal X-ray diffraction had formed. ^{1}H NMR confirms a 1:1 concentration of each monomer. Refinement of the crystal structure data confirmed cocrystallization of the two monomers, **1**.

^{1}H NMR: (400 MHz, 25 °C, ppm, DMSO-d6) δ 8.94 (d, 4H), 8.75 (s, 1H), 8.73 (s, 1fH), 8.18 (d, 2H), 8.16 (d, 2H), 8.06 (d, 2H), 7.59 (m, 6H), 7.48 (m, 6H)

*Synthesis of bulk **1**.*

An equimolar solution of **An**^{Py} and **An**^{COOH} (25 mM) in 20 mL THF was cast into a Pyrex petri dish and allowed to evaporate on a hot plate set to 30 °C, swirling to ensure as even of a coating as possible. Evaporation produced a bright yellow crystalline powder identified as **1** by ^{1}H NMR spectroscopy and PXRD.

*Synthesis of X-ray quality crystals of 9-pyridyldianthracene-9'-carboxylic acid (**1-Dimer**).*

Single crystals of **1** were loaded into a quartz tube and the headspace was deoxygenated with N_2. The tube was quickly sealed with a screw top cap and electrical tape. The crystals were irradiated with 365 nm light for 8 h, gently shaking the tube every hour to ensure efficient irradiation. SCXRD and ^{1}H NMR spectroscopy confirmed the product to be **1-Dimer**.

*Bulk solid-state synthesis of **1-Dimer**.*

20 mL of a 25 mM solution of equimolar **An**^{Py} and **An**^{COOH} in THF was cast into a Pyrex dish and manually swirled until the solvent evaporated to dryness and a yellow crystalline powder remained. The dish was sealed under a quartz plate with silicon grease and the headspace was flushed with nitrogen gas (N_2) through a hypodermic needle for 15 minutes, removing the needle under active nitrogen flow and pressing the plate to seal.

The dish was irradiated under a 365 nm lamp for 5 hours, after which point a color change from yellow to beige was observed. Photocycloaddition was also accomplished using a 427 nm, 456 nm, and solar simulator (1 sun intensity) for 2 h, 2 h, and 12 h, respectively. ^{1}H NMR of the resultant powder was revealed the product to be 94.3% **1-Dimer**, which is a mixture comprised of **diAn$^{Py/COOH}$** (**2**, 60.7%), **diAn^{Py2}** (**3**, 18.7%), and **diAn^{COOH2}** (**4**, 20.7%) with 5.7% residual monomer (Figure SX).

*Isolation of **2** and **3** from solid-state-synthesized **1-Dimer**.*

A sample of **1** was washed with MeOH until the filtrate was colorless and free of monomers. The remaining solids were dissolved in dichloromethane (DCM) by dropwise addition of minimal triethylamine (NEt_3). Silica gel was added to the solution and the solvent was removed under reduced pressure until the silica gel became a free-flowing powder. The loaded silica gel was loaded atop a silica gel column and the column was run using a 19:1:1.5 DCM:MeOH:NEt_3 eluent. The first fraction (Rf: 0.75) was evaporated to dryness and washed once with MeOH (10 mL) and twice with THF (10 mL). The remaining solids were air dried and identified as 9,9'-dipyridyldianthracene (**3**). To collect ESI-MS data, a sample of **3** was suspended in MeOH and 10 μL of 12 M HCl was added. Most of the colorless powder dissolved, and the solution was filtered and diluted for analysis.

The second fraction (Rf: 0.58) was evaporated to dryness and redissolved in MeOH. Hydrochloric acid (HCl, 12 M) was added dropwise until neutral, at which point a colorless powder precipitated from solution. The solution was filtered and the solids were washed with MeOH (3x10 mL). Pyridine was added dropwise to the filtrate until additional colorless powder precipitated. The suspension was filtered and the solids were washed thrice with MeOH (10 mL). Both samples of solids were identified as **diAn$^{Py/COOH}$** (**2**).

3:

^{1}H NMR (400 MHz, 25 °C, ppm, CD_2Cl_2) δ 5.49 (s, 1H); 6.20 (d, 2H); 6.67 (t, 2H); 6.79 (t, 2H); 6.96 (d, 2H)

$^{13}C\{^{1}H\}$ NMR (151 MHz, 25°C, ppm, CD_2Cl_2) δ 55.20, 63.58, 125.91, 126.37, 128.30, 128.95, 143.38, 147.48, 153.19

ESI-MS(+ve) m/z: Calculated: 511.2174, 512.2208, 513.2241, 514.2275 (+H^+) Found: 511.2147, 512.2208

Crystals suitable for analysis by SCXRD were grown by heating a sample of **3** in 2-methyl tetrahydrofuran to 65 °C for 1 h, filtering the solution, and allowing it to slowly evaporate at room temperature with toluene as the antisolvent. The resultant colorless plate crystals were used in X-ray analysis.

2:

^{1}H NMR (400 MHz, 25 °C, ppm, dmso-d6) δ 5.56 (s, 1H), 5.72 (s, 1H), 6.04 (d, 2H), 6.68 (t, 2H), 6.82 (m, 8H), 6.93 (d, 2H), 7.00 (m, 2H), 13.59 (s, 1H)

$^{13}C\{^{1}H\}$ NMR (151 MHz, 25°C, ppm, dmso-d6) δ 53.30, 54.30, 62.29, 65.70, 125.27, 125.32, 125.66, 125.73, 126.55, 127.46, 127.59, 127.85, 142.24, 142.29, 142.66, 146.54, 152.20, 174.61

ESI-MS(+ve) m/z: Calculated: 478.1807, 479.1841, 480.1874, 481.1908 (+H^+) Found: 478.1781, 479.1809

Crystals suitable for analysis by SCXRD were grown by heating a sample of **2** in THF to 65 °C for 1 h, filtering the solution, and allowing it to slowly evaporate at room temperature with toluene as an antisolvent. The resultant colorless block crystals were used in X-ray analysis.

*Solution-state synthesis of **2**.*

A 15 mL solution of **An^{Py}** (170 mg, 0.666 mmol) and **An^{COOH}** (148 mg, 0.666 mmol) in THF was sparged with N_2 was sealed and sparged with N_2 before irradiating overnight with a 365 nm lamp for 48 h. The solution was cooled to room temperature, and the resultant colorless powder was filtered and washed with methanol (3x 10 mL). The solid product was purified by column chromatography using a 19:1:1.5 DCM:MeOH:NEt_3 eluent, collecting the second fraction. The product was redissolved in MeOH, and hydrochloric acid (HCl) was added dropwise until neutral, at which point a colorless powder precipitated from solution. The solution was filtered and the solids were washed with MeOH (3x10 mL). Pyridine was added dropwise to the filtrate until additional colorless powder precipitated. The suspension was filtered and the solids were washed thrice with MeOH (10 mL). Both samples of solids were identified as **2**.

*Independent synthesis of **3**.*

A 15 mL solution of **An^{Py}** (296 mg, 1.16 mmol) in THF was sealed and sparged with N_2 before irradiating overnight with a 365 nm lamp for 48 h. The solution was cooled to room temperature, and the resultant colorless powder was filtered and washed with methanol (3x 10 mL) to produce **3**, which was confirmed by ^{1}H NMR spectroscopy.

Solution-state retrocyclization kinetics, activation parameters, and half-lives.

Samples of the isolated diAn species (**2**, **3**, or **4**) were dissolved in dmso-d_6 at room temperature. After filtering off any undissolved solids, a ^{1}H NMR spectrum was recorded. The NMR tubes containing each dimer were exposed to high temperature (120, 130, and 140 °C) in a well-stirred oil bath to ensure consistent heating. The tubes were removed at designated timepoints, recording ^{1}H NMR spectra for each timepoint before resubmerging in the oil bath. This was repeated until at least 40% conversion to the retrocyclized product. Relative quantities of dimer and monomer were calculated using the integrals of their respective ^{1}H NMR signals. No other decomposition products were observed. The integral data was converted to the term α, which represents the relative composition of dimer in the solution, by the following equation:

$$\square = \frac{[\text{dimer}]}{[\text{dimer}] + [\text{monomer}]}$$

where [dimer] and [monomer] are the sum of integrals from the ^{1}H NMR signals. The plot of α vs time (s) was fit to a first order decay via least squares fitting. The resultant rate constants at different temperatures were used to construct an Eyring plot (i.e. ln(k/T) vs. T), from which the activation parameters ∆$H^‡$, ∆$S^‡$, ∆$G^{‡,\ 298.15K}$ were derived using the linear fit of the plot and following equations:

$$\square H^{\ddagger} = m * R$$

$$\square S^{\ddagger} = R * (b - \ln\left(\frac{k_B}{h}\right))$$

$$\Delta G^{\ddagger,\,298.15\ K} = \Delta H^{\ddagger} - T^{*}\Delta S^{\ddagger} = \Delta H^{\ddagger} - 298.15 * \Delta S^{\ddagger}$$

where m is the slope of the linear fit of the Eyring plot, b is the y-intercept of the linear fit of the Eyring plot, R is the ideal gas constant in J/(mol*K), k_B is the Boltzmann constant in J/K, h is Planck's constant in J*s. Using this information, the rate constant and half-life of each compound in solution was found at room temperature (298.15 K) using the following equations:

$$\ln\left(\frac{k^{298.15\ K}}{298.15}\right) = m * \frac{1}{298.15} + b$$

$$k^{298.15\ K} = e^{\frac{m}{298.15}+b} * 298.15$$

$$t_{\frac{1}{2}}^{298.15\ K} = \frac{\ln(0.5)}{-k^{298.15\ K}}$$

Kinetics data, traces, fitting, and Eyring plots are found in Figures SX-SX. Activation parameters and half-lives are summarized in Table 1.

*Charge-discharge cycling of **1/1-Dimer**.*

Bulk microcrystalline **1** in a Pyrex dish was sealed under a quartz plate with silicone grease and the headspace was flushed with N_2 through a hypodermic needle for 15 minutes, removing the needle under active N_2 flow and pressing the plate to seal. The dish was irradiated under a 365 nm or 456 nm lamp for 1.5-2 h. The quartz plate was removed and the grease was cleaned off. An aliquot of the powder was removed for analysis by ^{1}H NMR, and the Pyrex dish was covered with aluminum foil and placed in a Quincy Lab model 10GC oven set to 150 °C for 1.5 h. Annealing was performed in air. An aliquot of the powder was removed for analysis by ^{1}H NMR, and the Pyrex dish was resealed under a quartz plate and the irradiation/annealing process was repeated until insufficient solids remained in the dish for analysis. Relative dimer concentration was calculated by finding the percent composition of the integrals of ^{1}H NMR signals corresponding to **1** versus the sum of integrals for all An-based signals. A sample calculation is shown in Figure SX.

*Cycling of **2**, **3**, and **4**.*

Samples of purified dimers were analyzed by ^{1}H NMR. A solid sample of dimer was placed in a Pyrex dish, covered with aluminum foil, and placed in a 150 °C oven for 1.5 h. An aliquot of was analyzed by ^{1}H NMR. The Pyrex dish was sealed under a quartz plate with silicone grease and the headspace was flushed with N_2 through a hypodermic needle for 15 minutes, removing the needle under active nitrogen flow and pressing the plate to seal. The dish was irradiated under a 365 nm lamp for 1.5-3 h. The quartz plate was removed and the grease was cleaned off. An aliquot of the powder was removed for analysis by ^{1}H NMR. For **3** and **4**, little or no conversion was detected after initial irradiation, so the dish was resealed under a quartz plate with a N_2 atmosphere and allowed to irradiate for an additional 24 h, analyzing the resultant solids by ^{1}H NMR.

B. NMR Spectroscopy

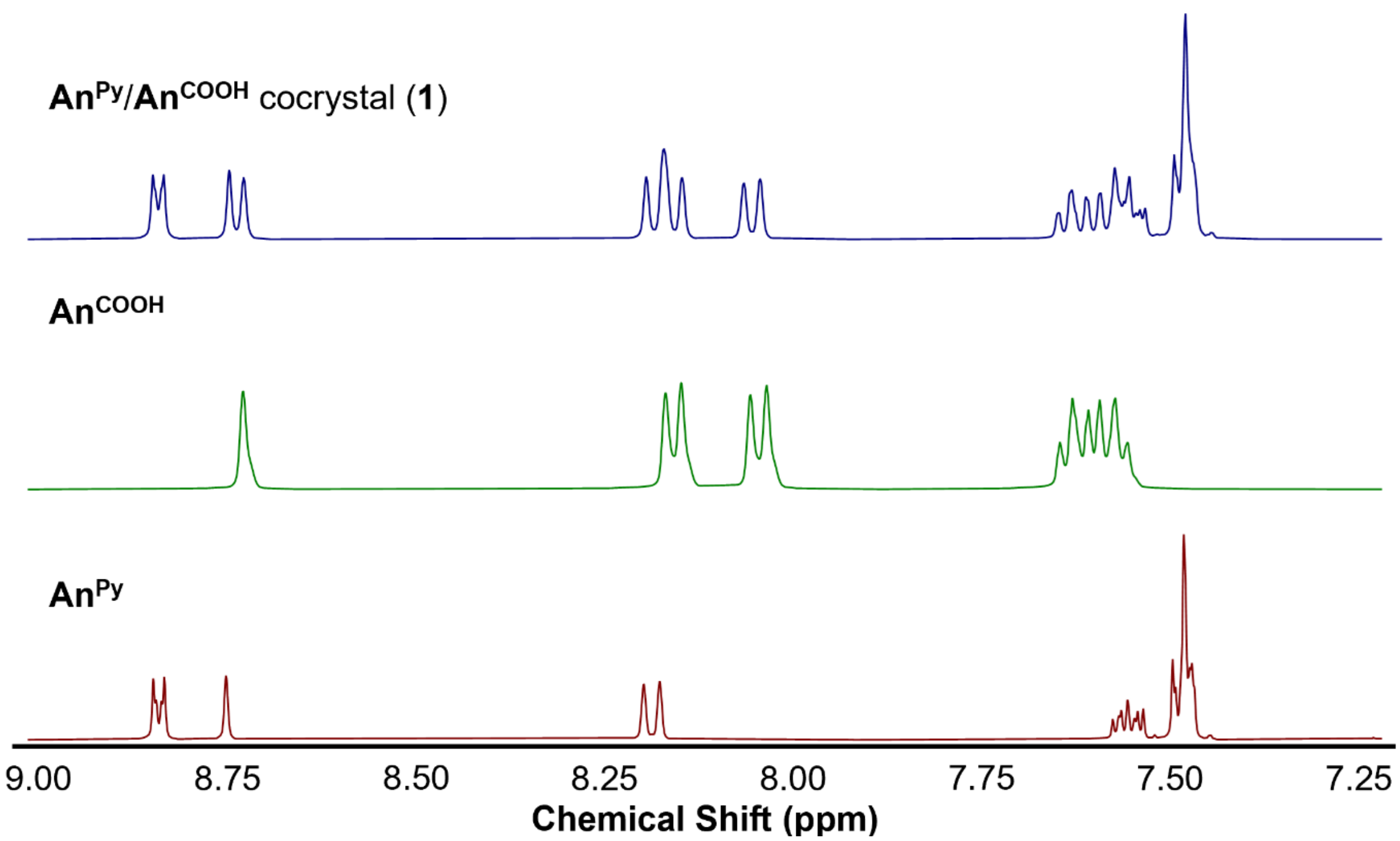

Figure S1. ^{1}H NMR spectra of **1** (top) and its two component monomers $\mathbf{An^{COOH}}$ (middle) and $\mathbf{An^{Py}}$ (bottom) in DMSO-d6 at 25 °C.

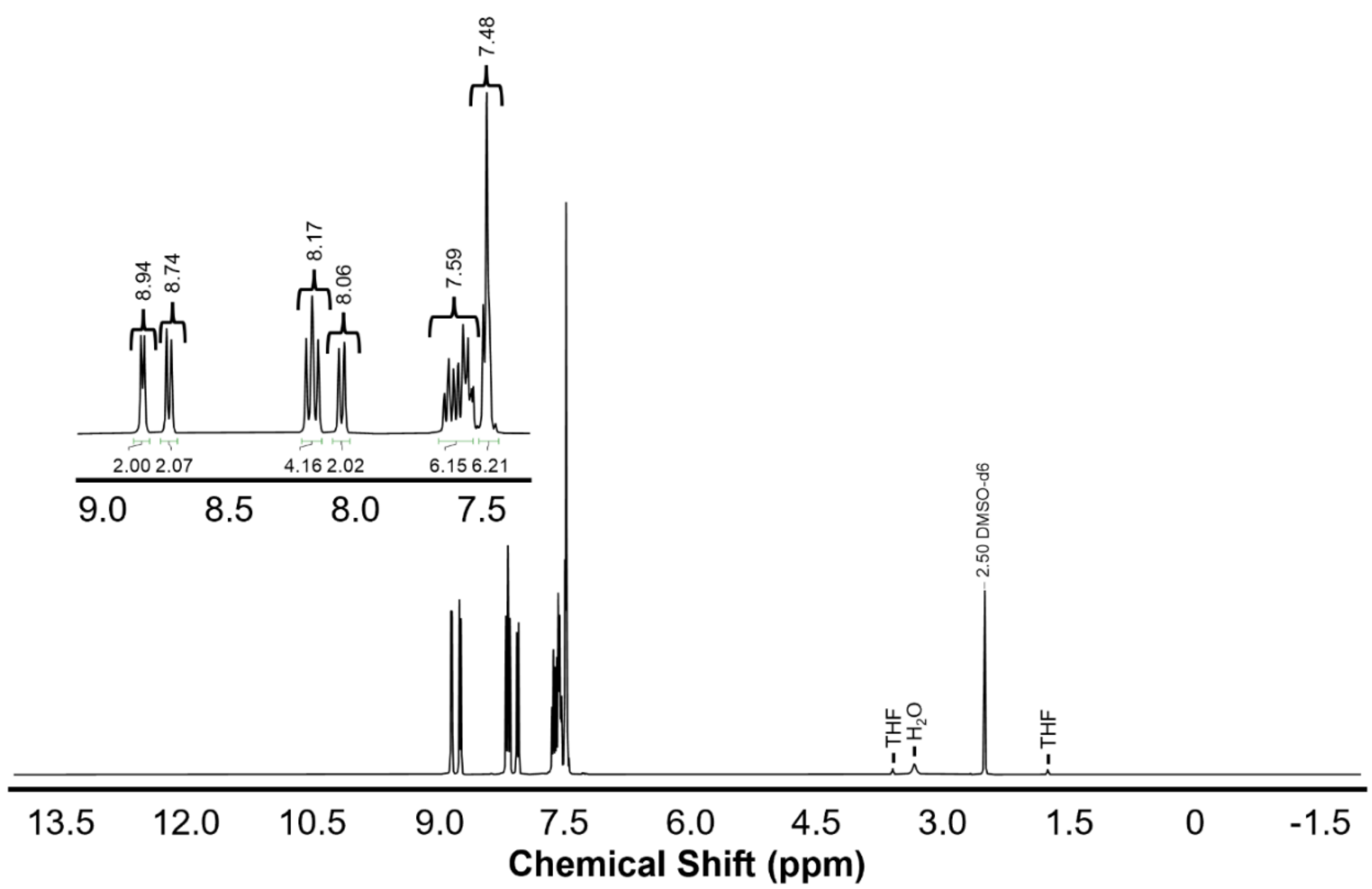

Figure S2. ^{1}H NMR spectra of **1** in DMSO-d6 at 25 °C.

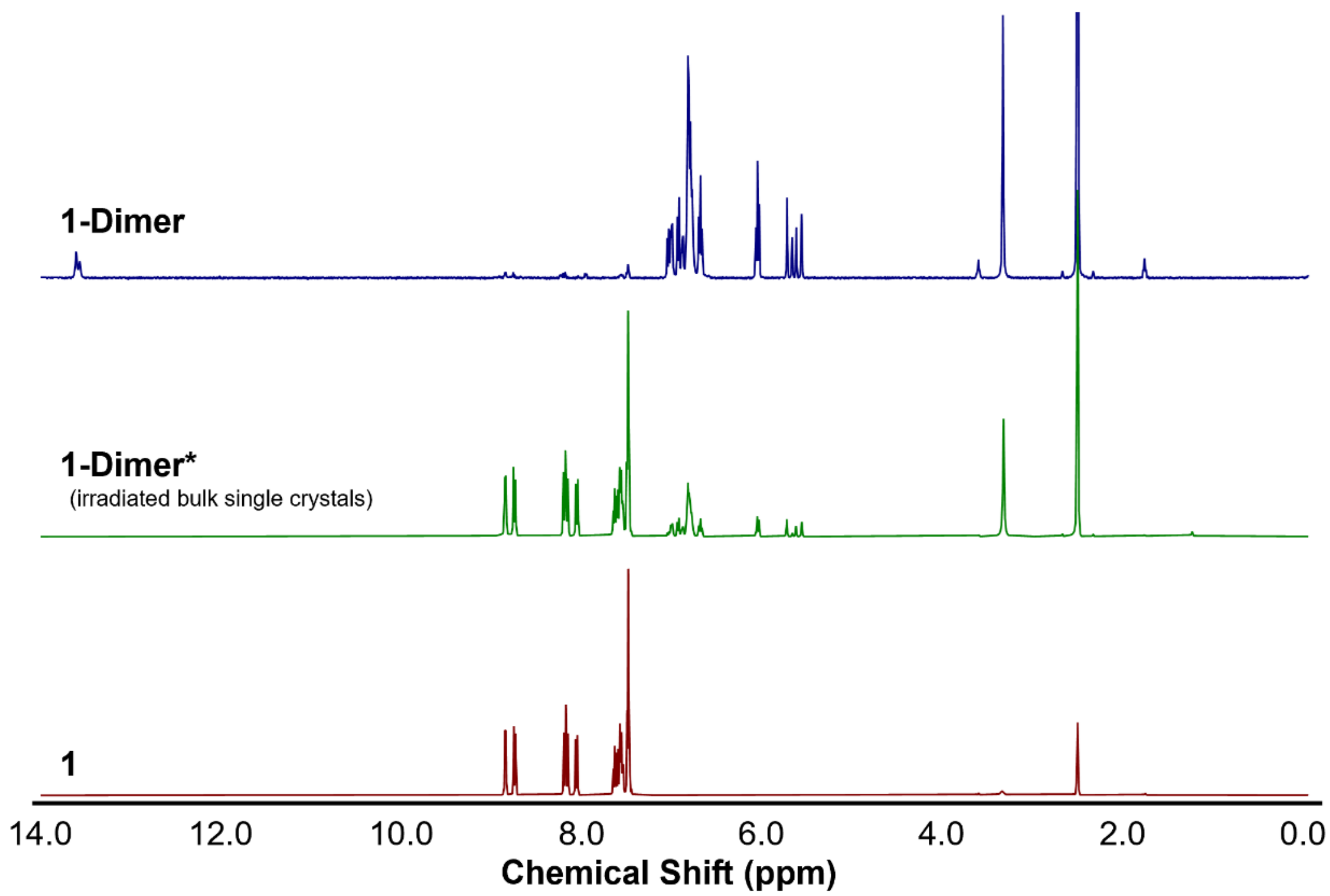

Figure S3. ^{1}H NMR spectrum of a bulk sample of SCXRD-quality crystals of **1** after irradiation with 365 nm light for 3 h in DMSO-d6 at 25 °C, indicating partial conversion to **1-Dimer**. Signals downfield of 7.5 ppm correspond with the monomer compound **1**, with signals between 5.5 and 7.5 corresponding to **1-Dimer**.

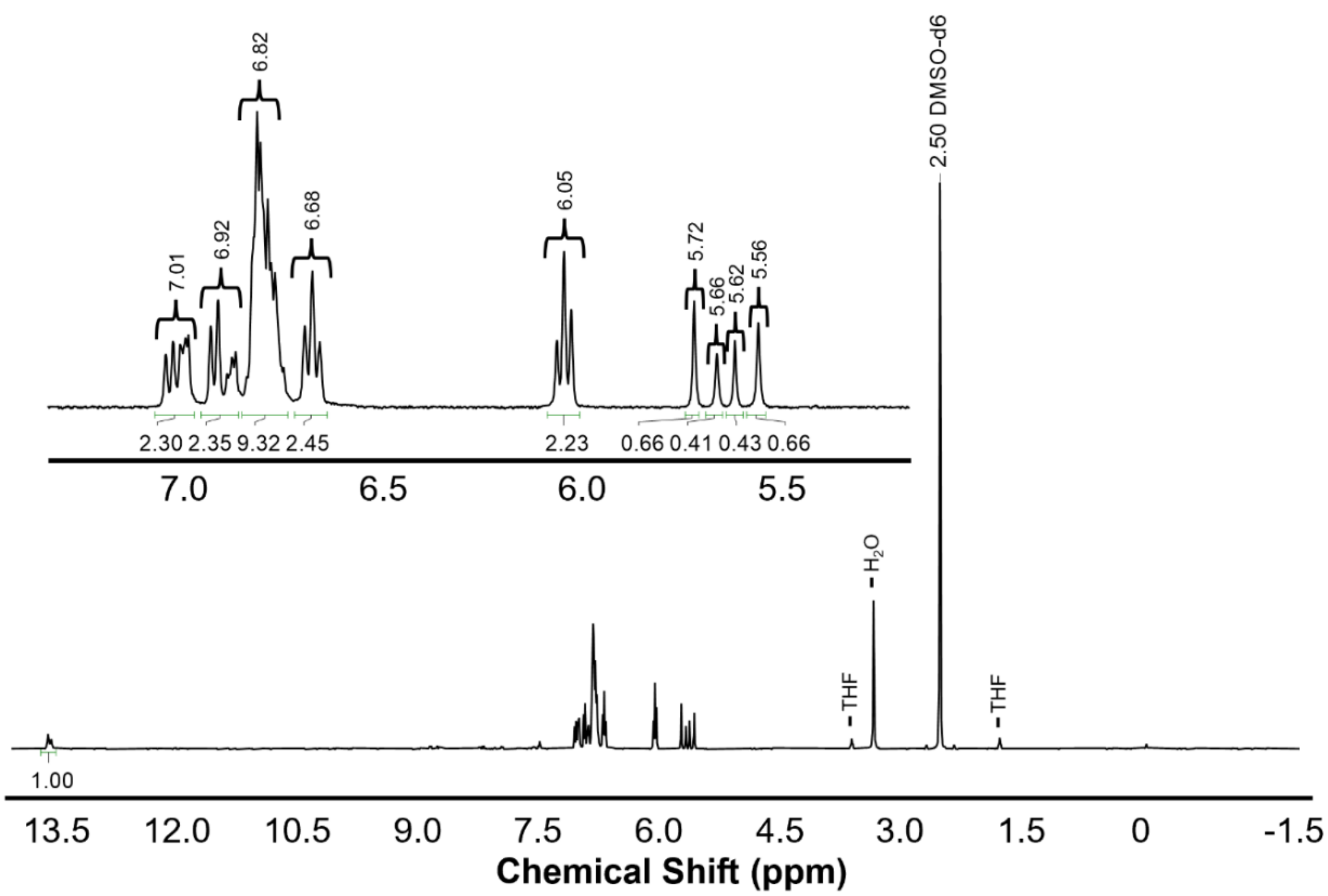

Figure S4. ^{1}H NMR spectrum **1-Dimer** in DMSO-d6 at 25 °C. Peaks between 7.5-9 ppm correspond to unreacted monomers.

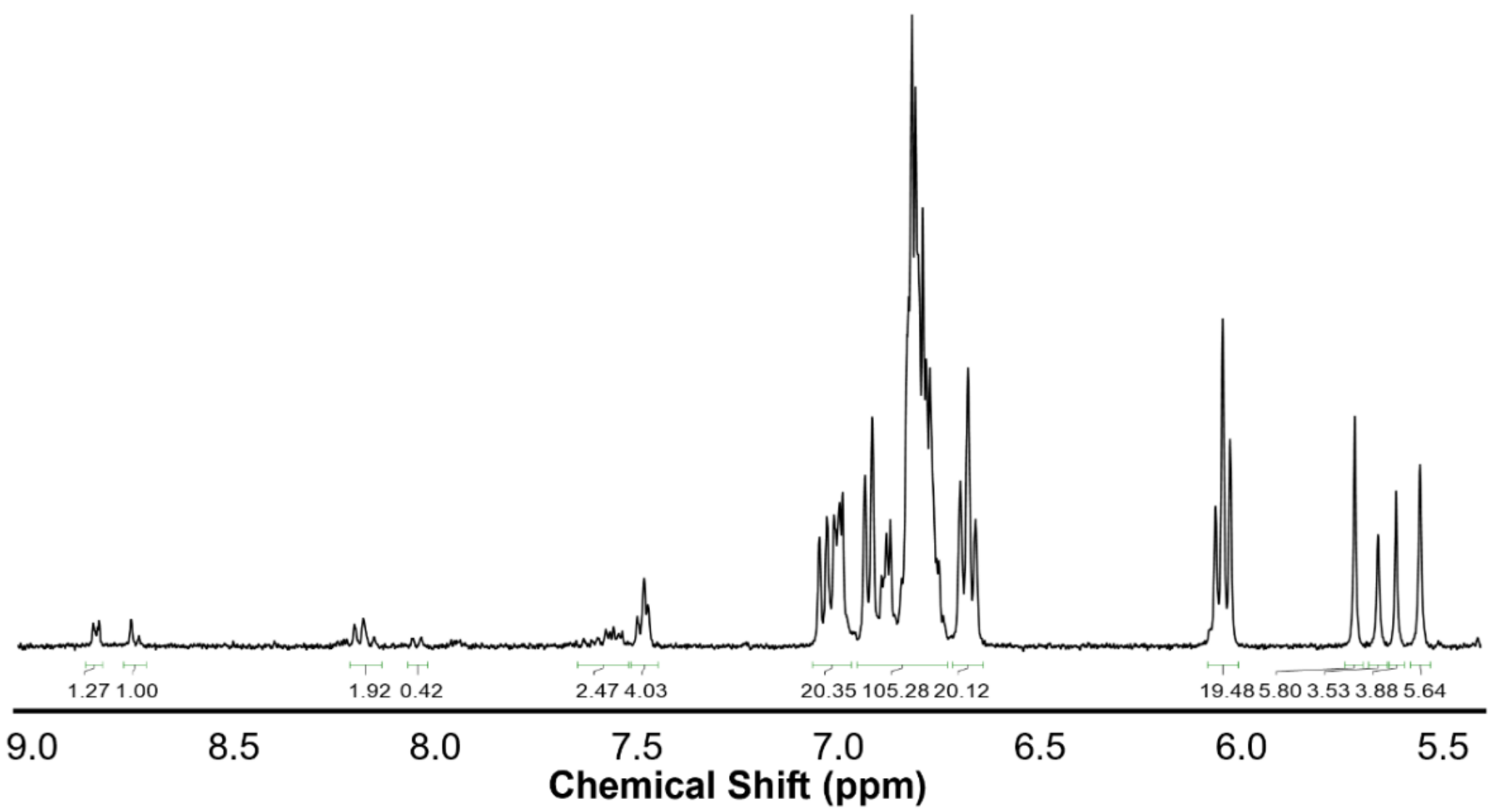

$$100\% * \frac{\int Dimer}{\int Monomer + \int Dimer} = 100\% * \frac{184.08}{11.11 + 184.08} = 94.3\%$$

Figure S5. ^{1}H NMR spectrum of a crude sample of **1-Dimer** synthesized by irradiation of a polycrystalline sample of **1** with 365 nm light for 2 h in DMSO-d6 at 25 °C with conversion calculations.

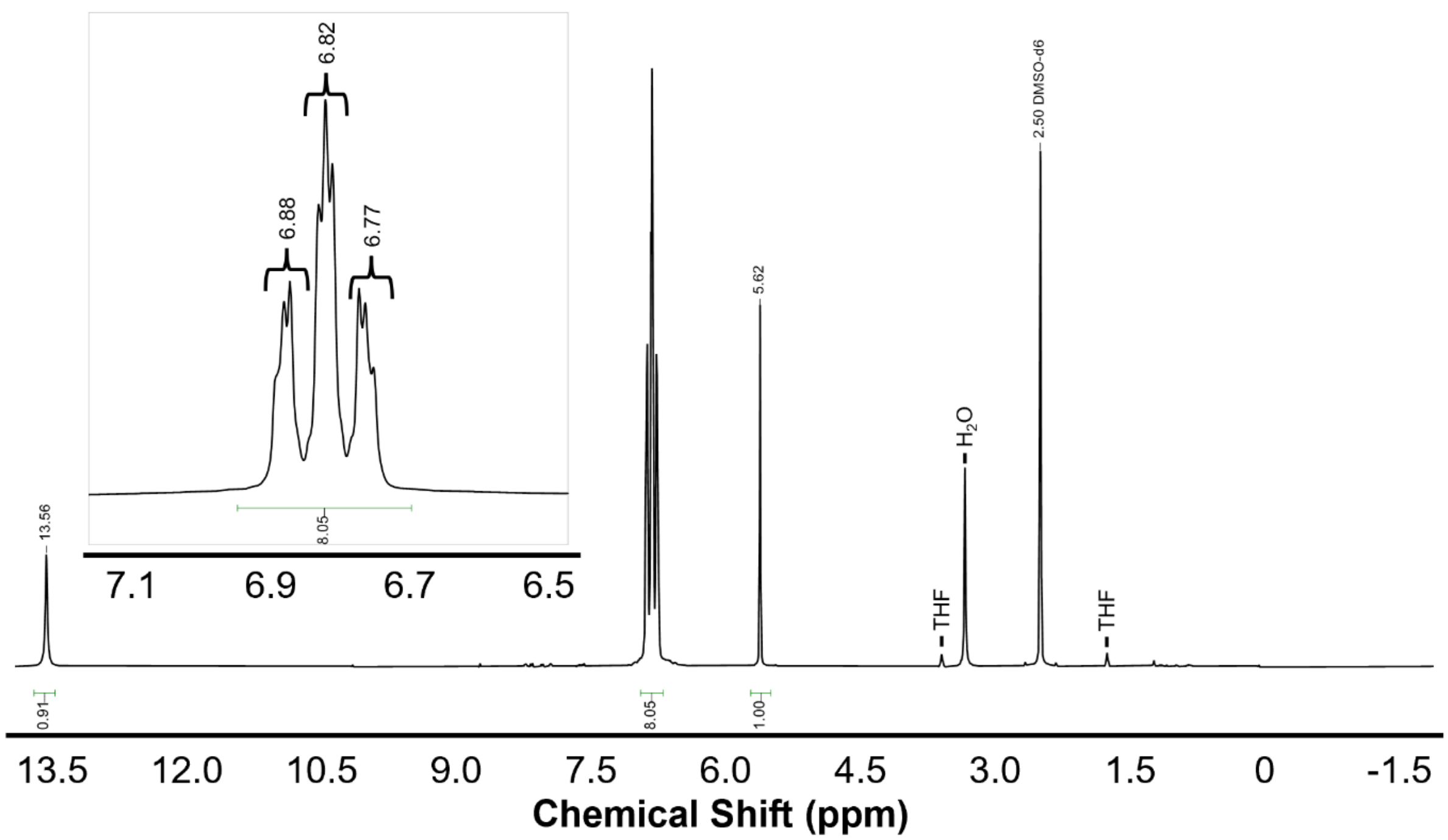

Figure S6. ^{1}H NMR spectrum **4** in DMSO-d6 at 25 °C.

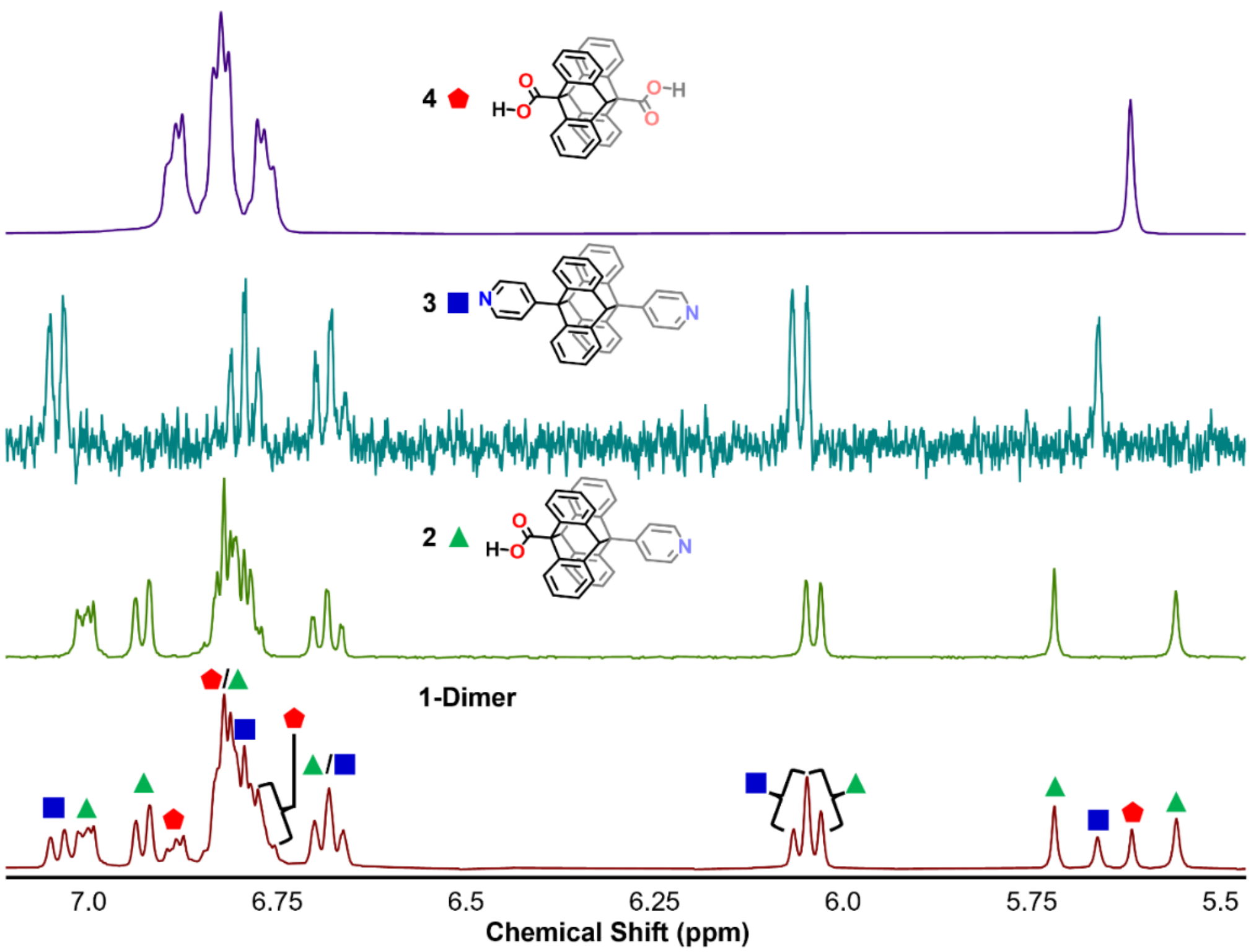

Figure S7. ¹H NMR spectra of **1-Dimer**, **2**, **3**, and **4** in DMSO-d6 at 25 °C. Assignment of the spectrum of **1-Dimer** is done using green triangles for **2**, blue squares for **3**, and red pentagons for **4**.

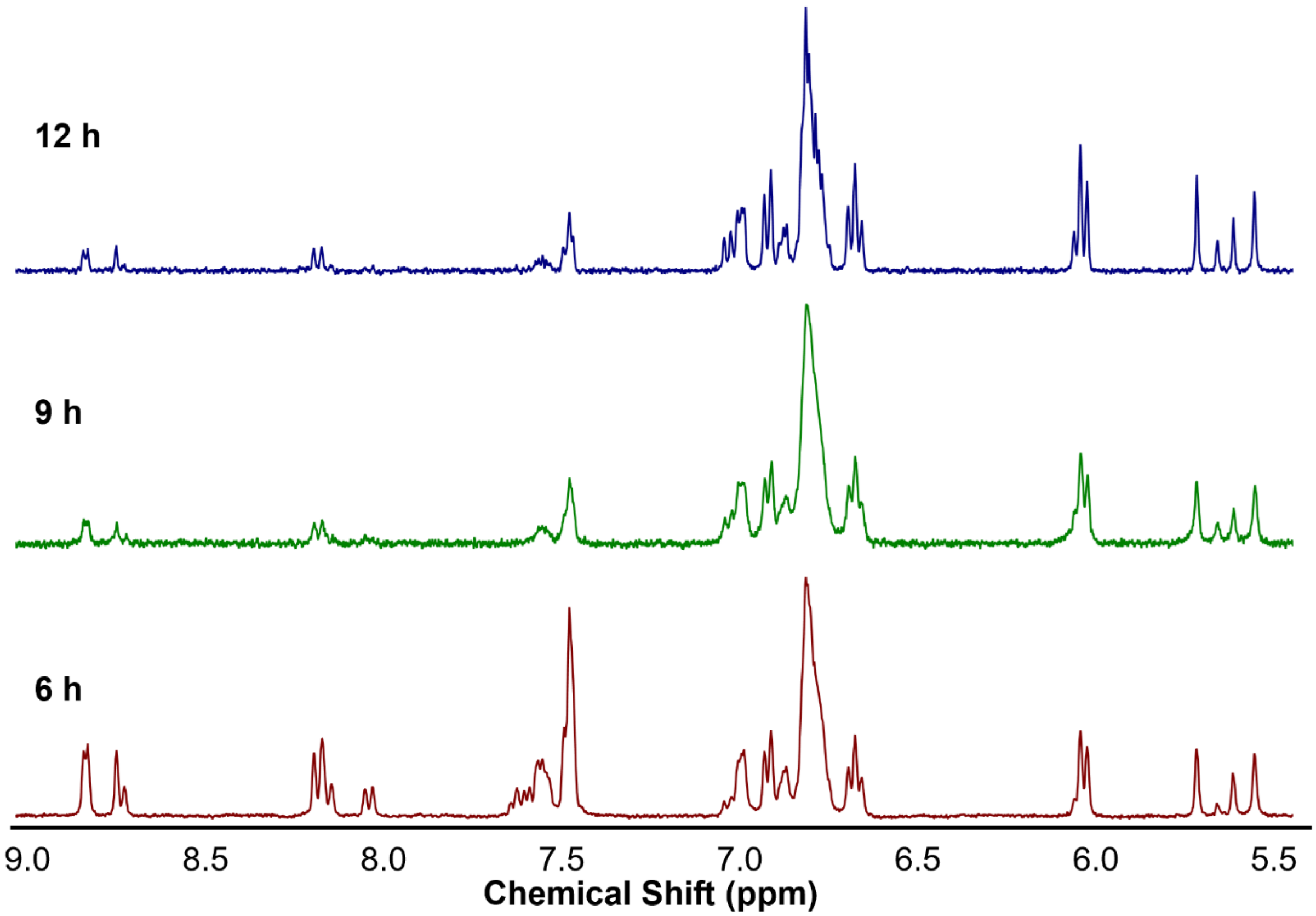

$$100\% * \frac{\int Dimer}{\int Monomer + \int Dimer} = 100\% * \frac{107.05}{11.85 + 107.05} = 90.0\% \ (@\ 12\ h)$$

Figure S8. ^{1}H NMR spectrum of a crude sample of **1-Dimer** synthesized by irradiation of a polycrystalline sample of **1** with simulated sunlight for 12 h in DMSO-d6 at 25 °C with conversion calculations.

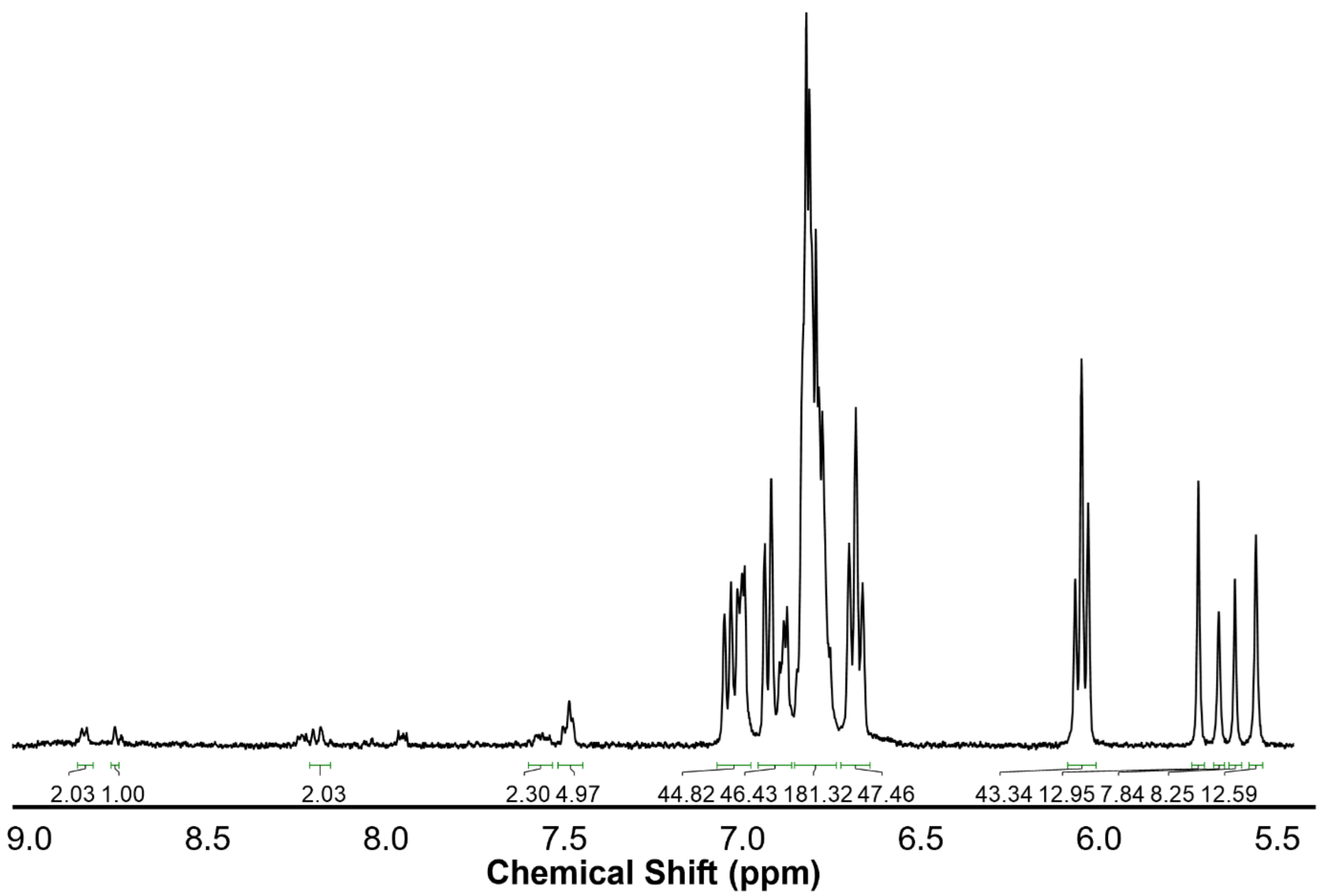

$$100\% * \frac{\int Dimer}{\int Monomer + \int Dimer} = 100\% * \frac{405.00}{12.33 + 405.00} = 97.0\%$$

Figure S9. ^{1}H NMR spectrum of a crude sample of **1-Dimer** synthesized by irradiation of a polycrystalline sample of **1** with 427 nm light for 6 h in DMSO-d6 at 25 °C with conversion calculations.

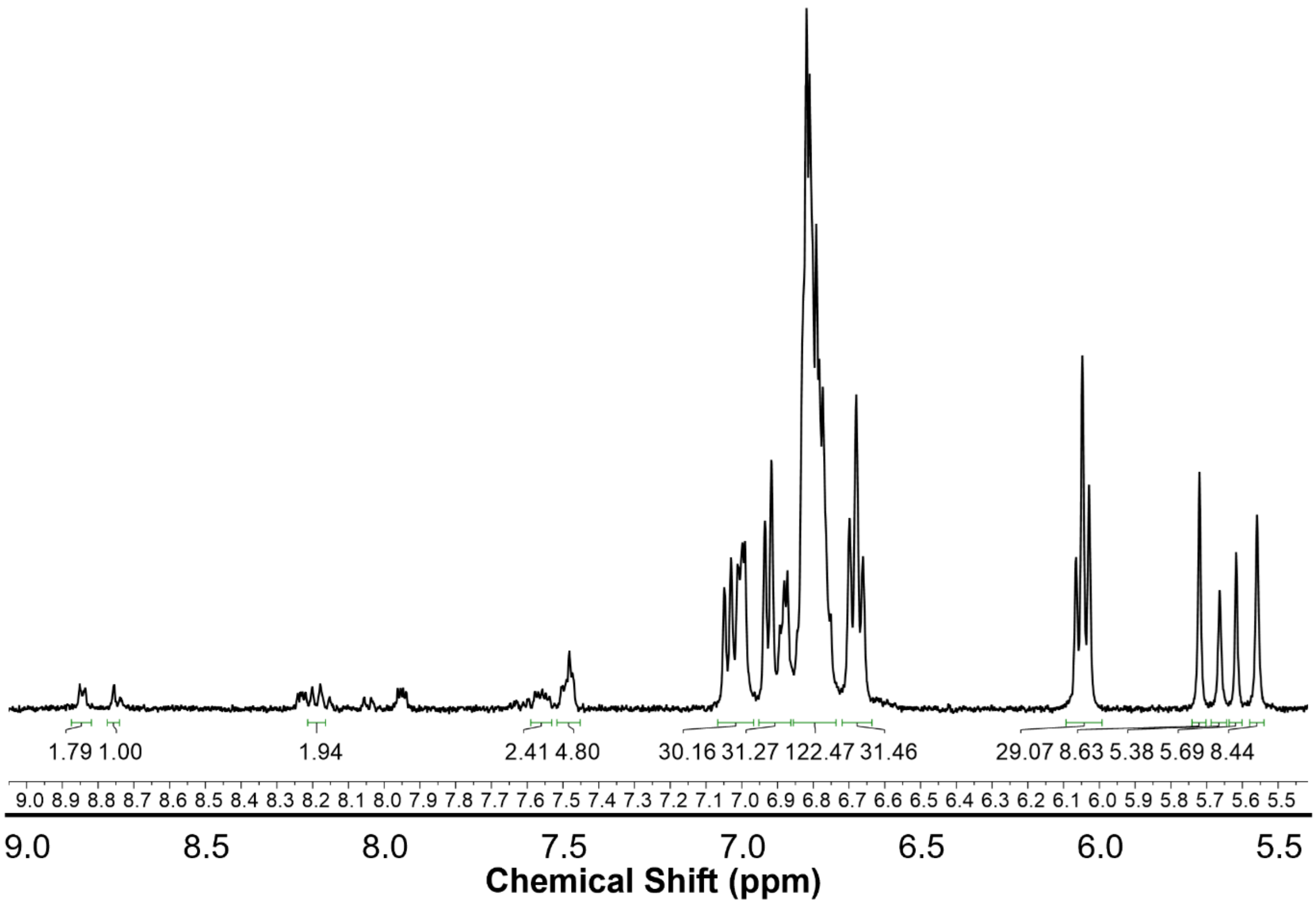

$$100\% * \frac{\int Dimer}{\int Monomer + \int Dimer} = 100\% * \frac{272.57}{11.94 + 272.57} = 95.7\%$$

Figure S10. ^{1}H NMR spectrum of a crude sample of **1-Dimer** synthesized by irradiation of a polycrystalline sample of **1** with 456 nm light for 6 h in DMSO-d6 at 25 °C with conversion calculations.

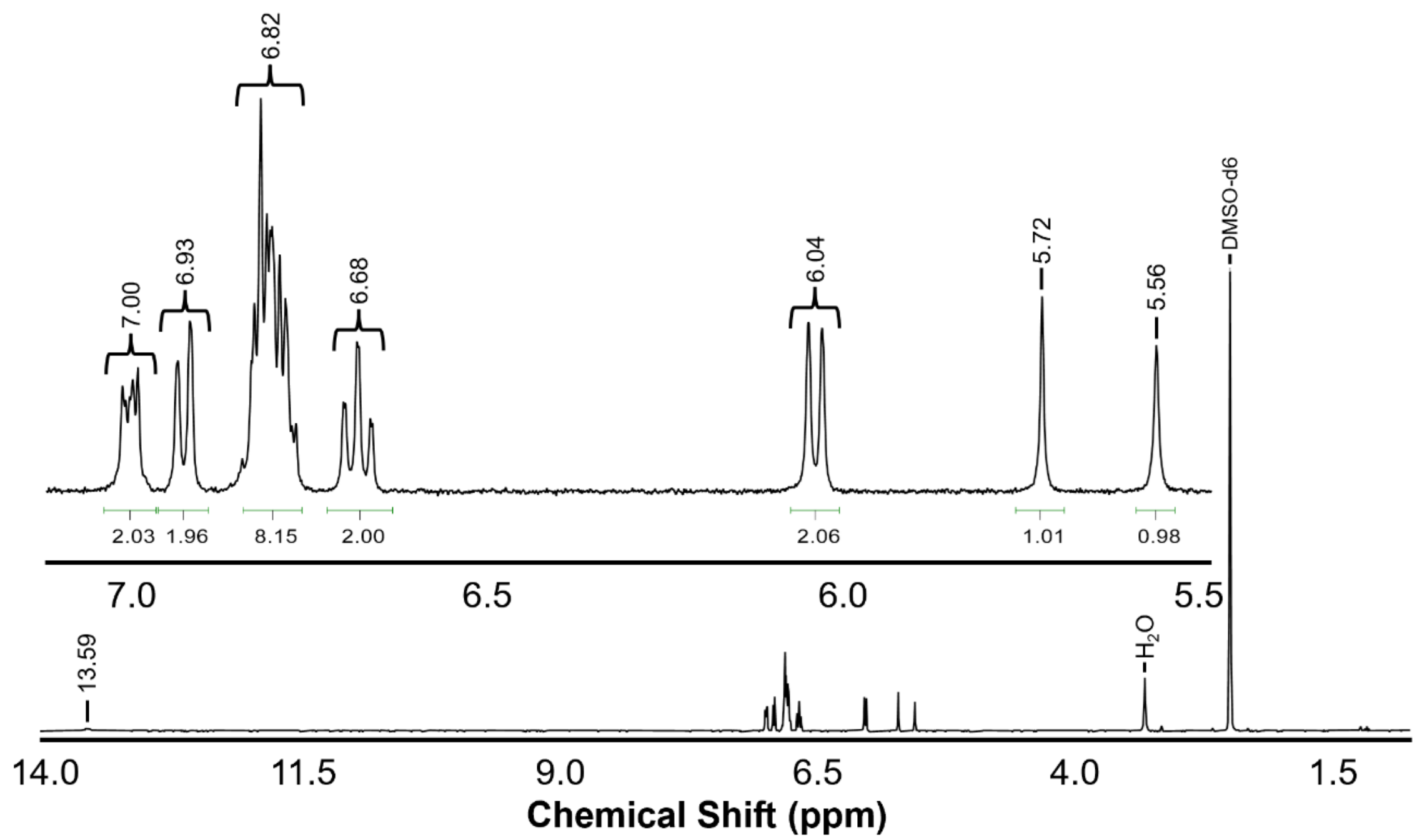

Figure S11. ^{1}H NMR spectrum of **2** in DMSO-d6 at 25 °C.

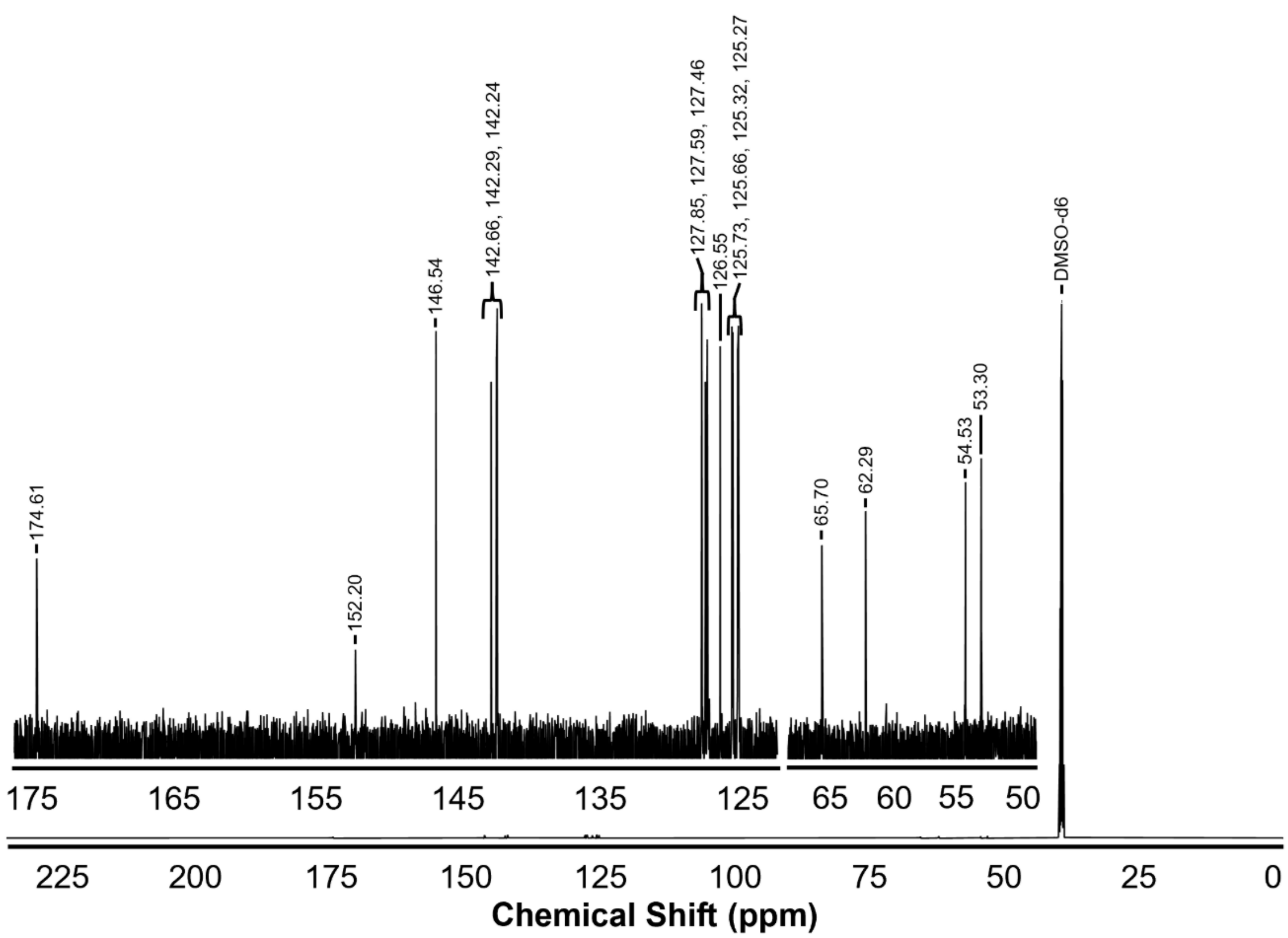

Figure S12. ^{13}C NMR spectrum of **2** in DMSO-d6 at 25 °C.

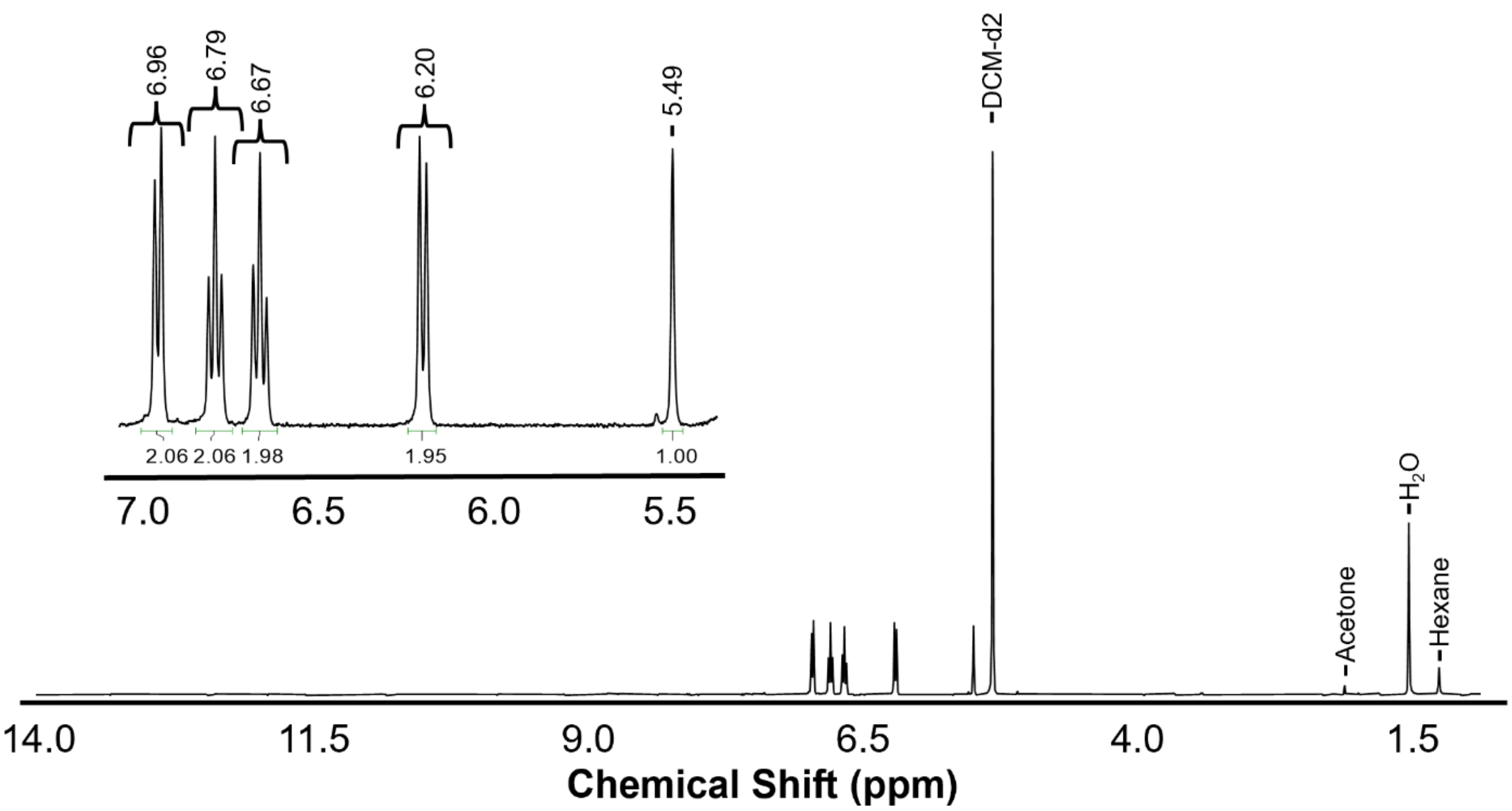

Figure S13. ^{1}H NMR spectrum of **3** in CD_2Cl_2 at 25 °C.

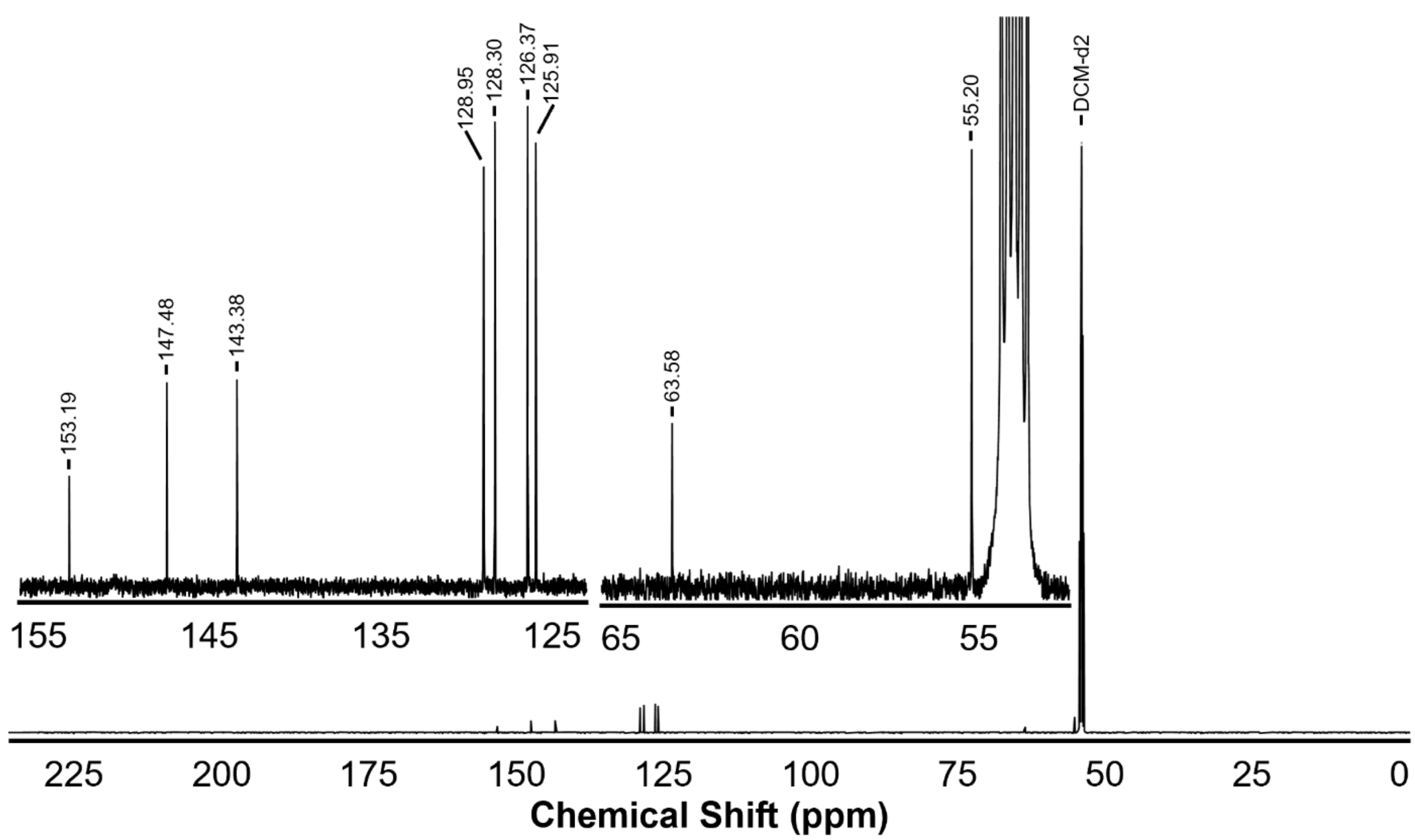

Figure S14. ^{13}C NMR spectrum of **3** in CD_2Cl_2 at 25 °C.

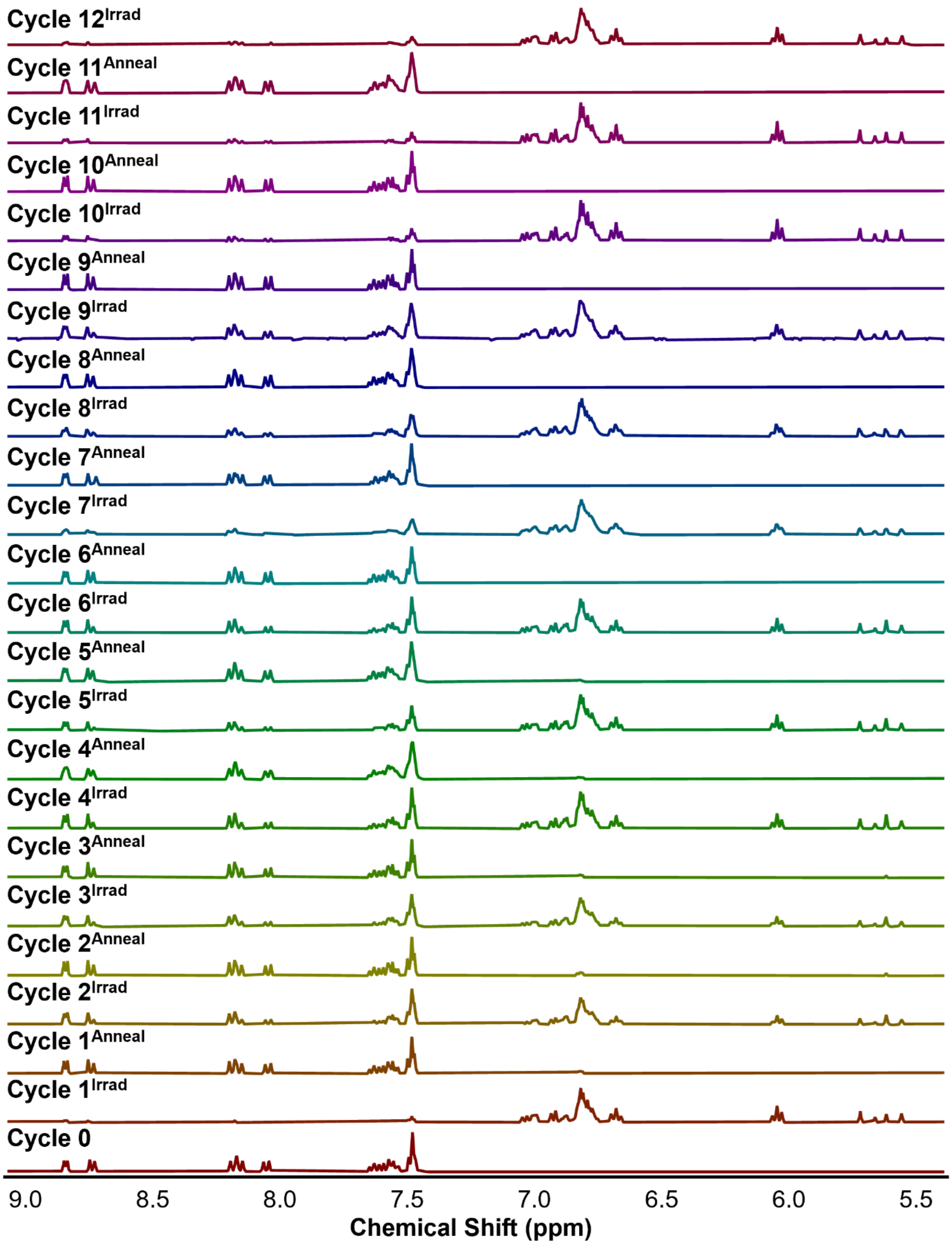

Figure S15. ^{1}H NMR spectra of aliquots from C-D cycling of **1/1-Dimer**, charging with 365 nm light. Spectra collected in DMSO-d6 at 25 °C.

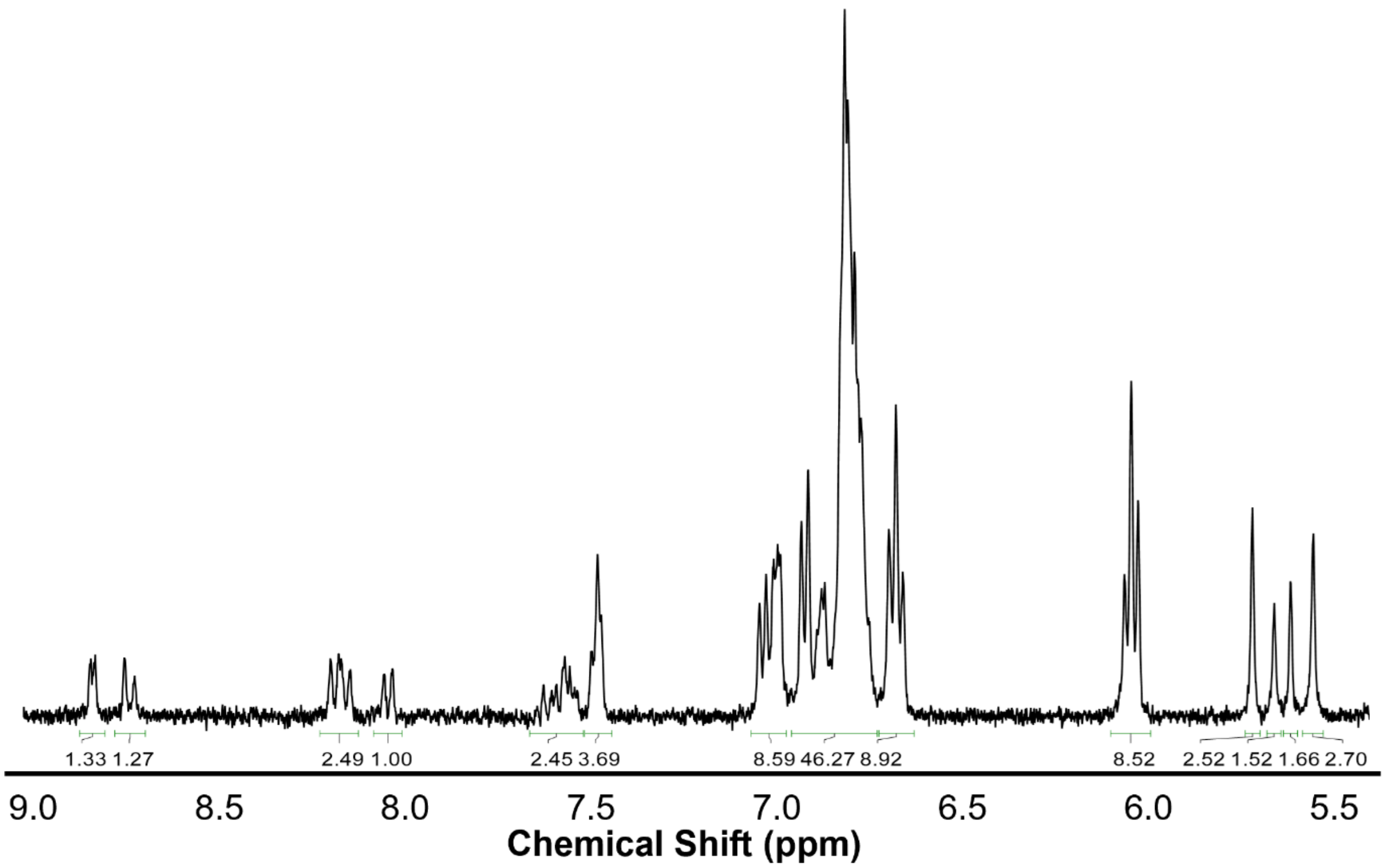

$$100\% * \frac{\int Dimer}{\int Monomer + \int Dimer} = 100\% * \frac{80.70}{12.23 + 80.70} = 86.8\%$$

Figure S16. Sample C-D SOC calculation on the 12th cycle of **1/1-Dimer**.

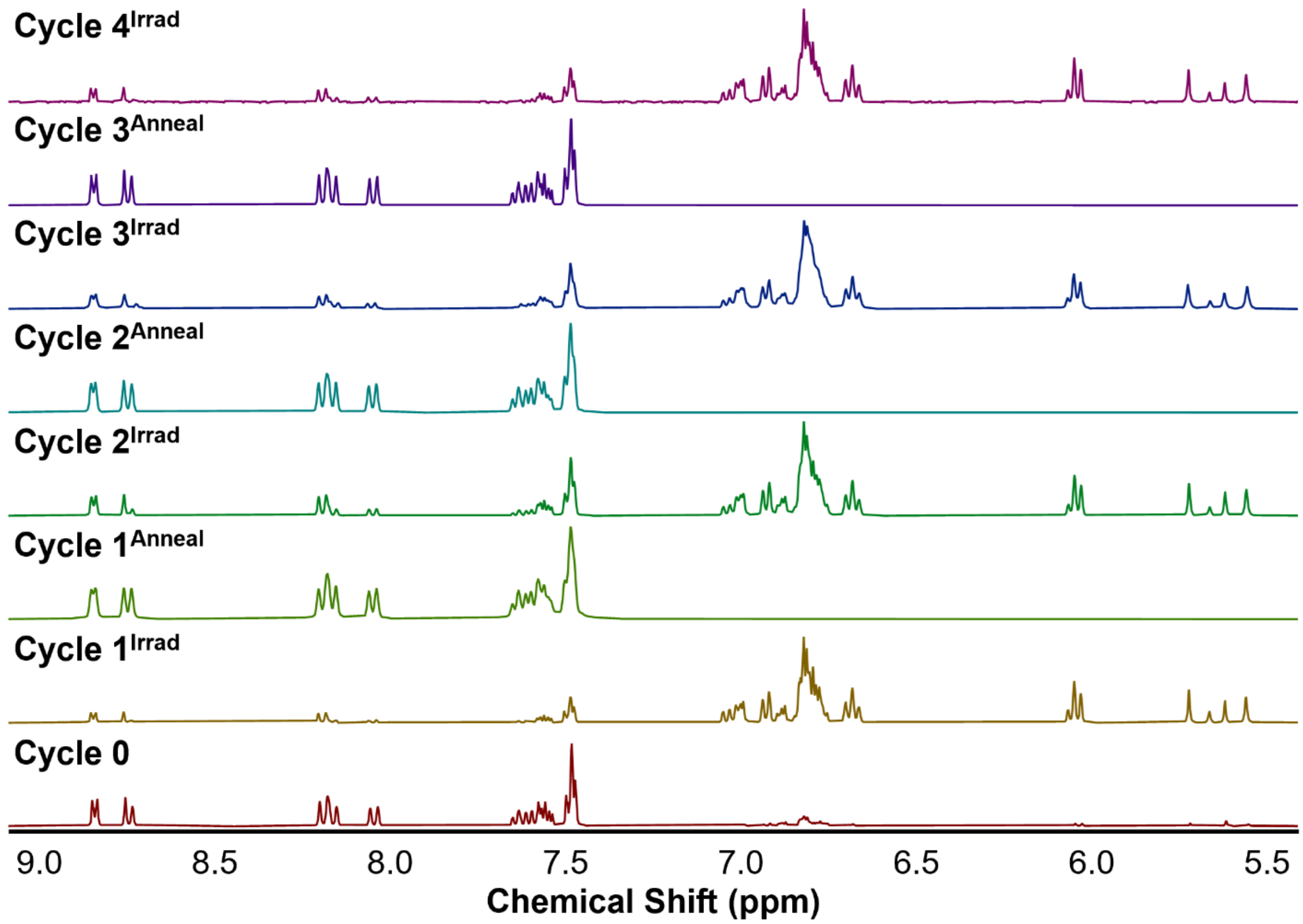

Figure S17. ^{1}H NMR spectra of aliquots from C-D cycling of **1/1-Dimer** under 456 nm irradiation. Spectra collected in DMSO-d6 at 25 °C.

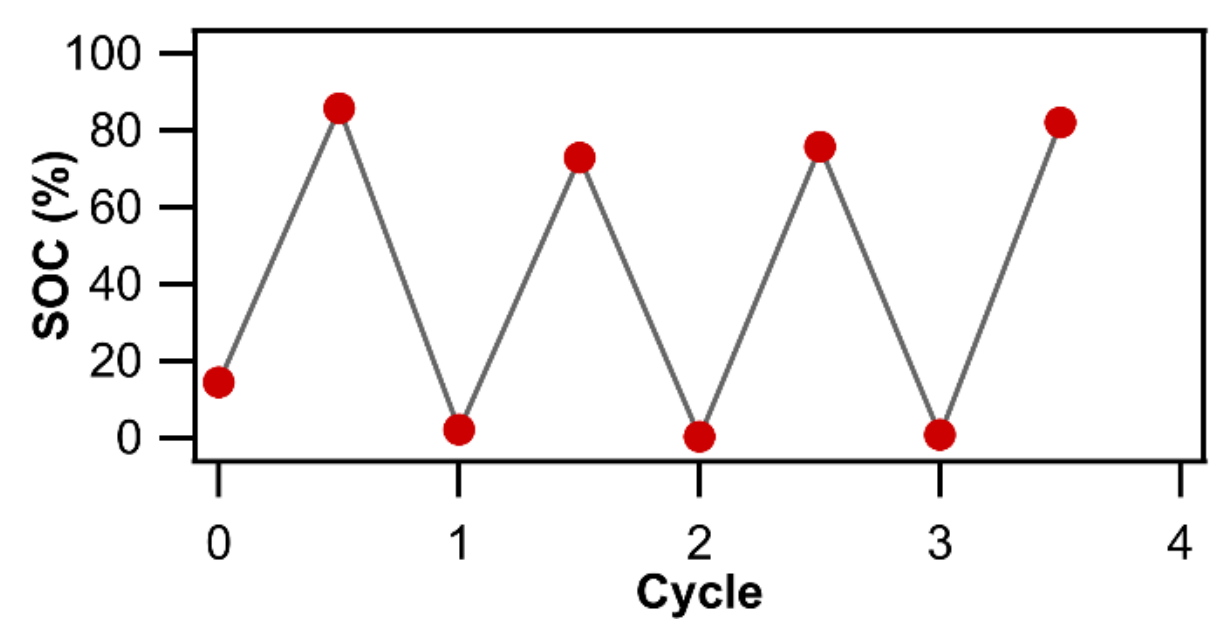

Figure S18. C-D profile of **1/1-Dimer** using 456 nm irradiation.

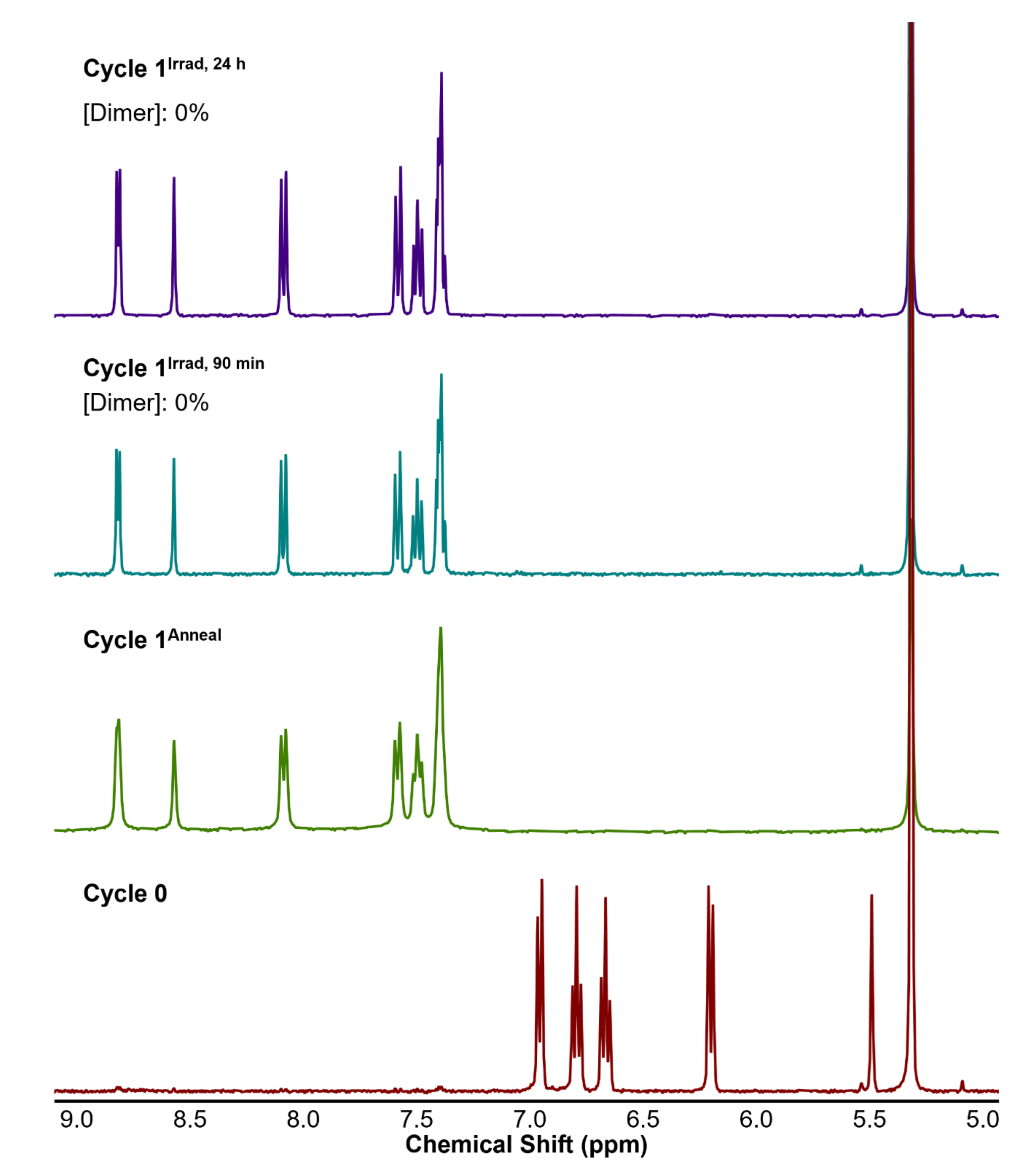

Figure S19. ^{1}H NMR spectra of aliquots from C-D cycling of **3** under 365 nm light. Spectra taken in DMSO-d6 at 25 °C.

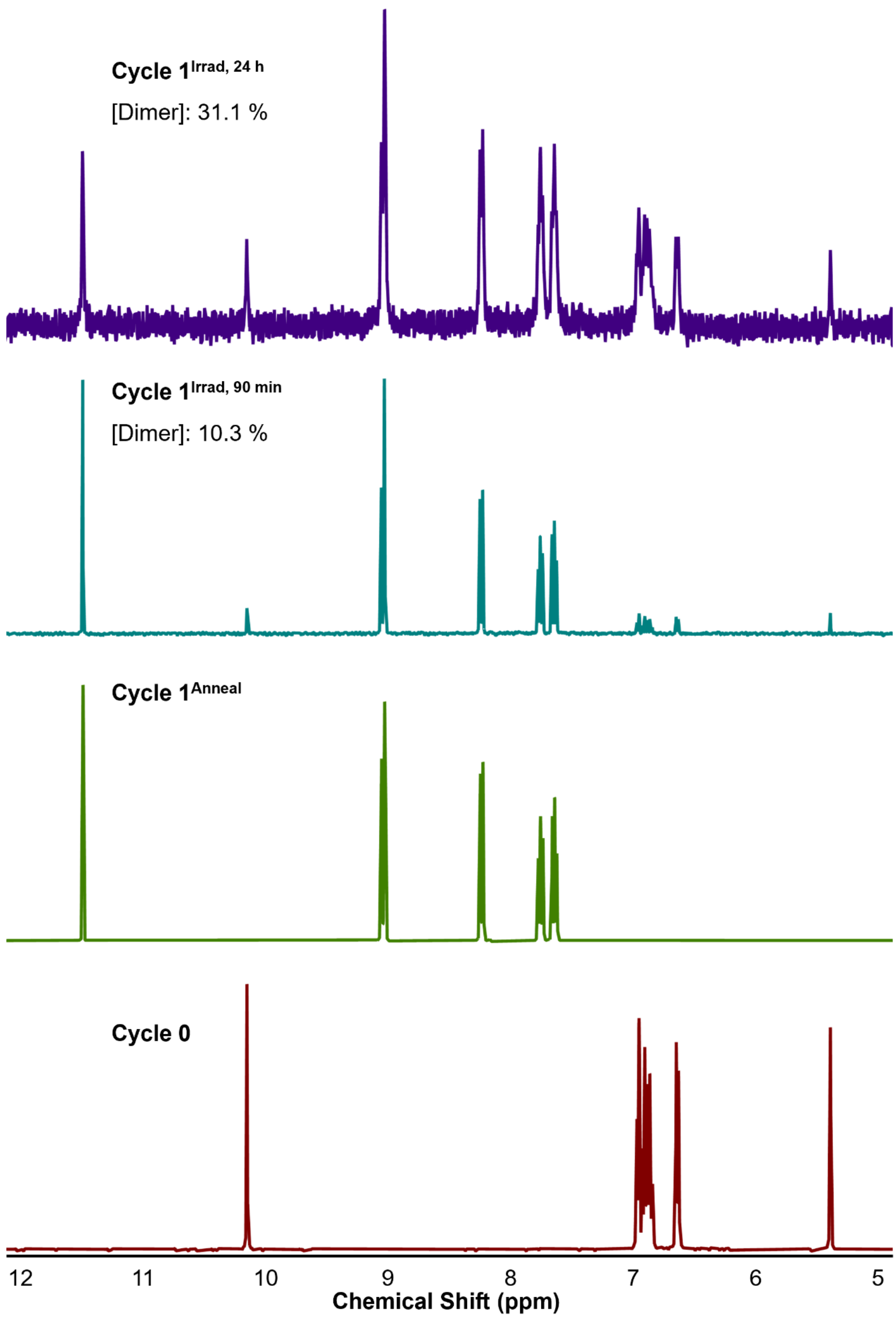

Figure S20. ^{1}H NMR spectra of aliquots from C-D cycling of **4** under 365 nm light. Spectra taken in DMSO-d6 at 25 °C.

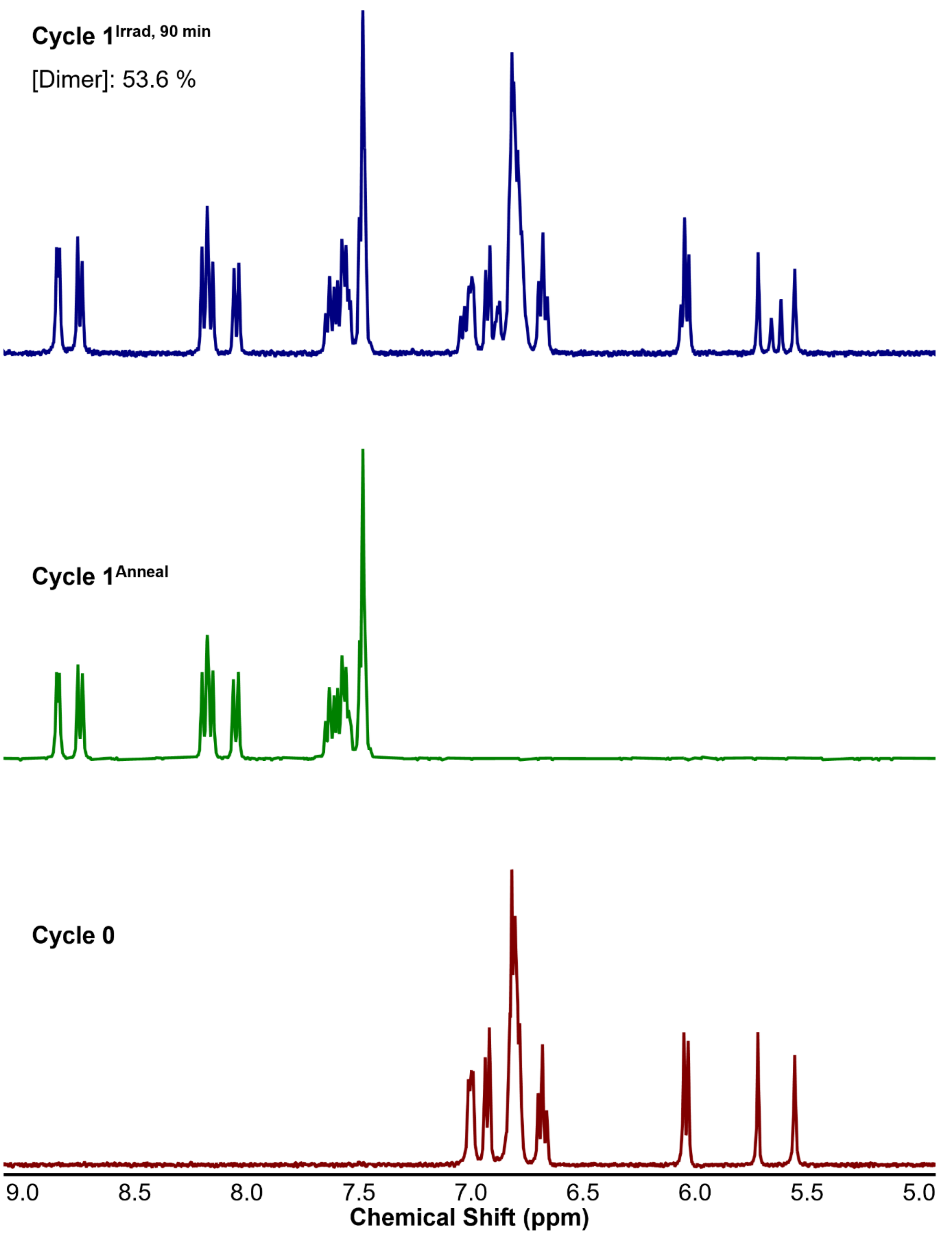

Figure S21. ^{1}H NMR spectra of aliquots from C-D cycling of **2** under 365 nm light. Spectra taken in DMSO-d6 at 25 °C.

C. Electrospray Ionization Mass Spectrometry

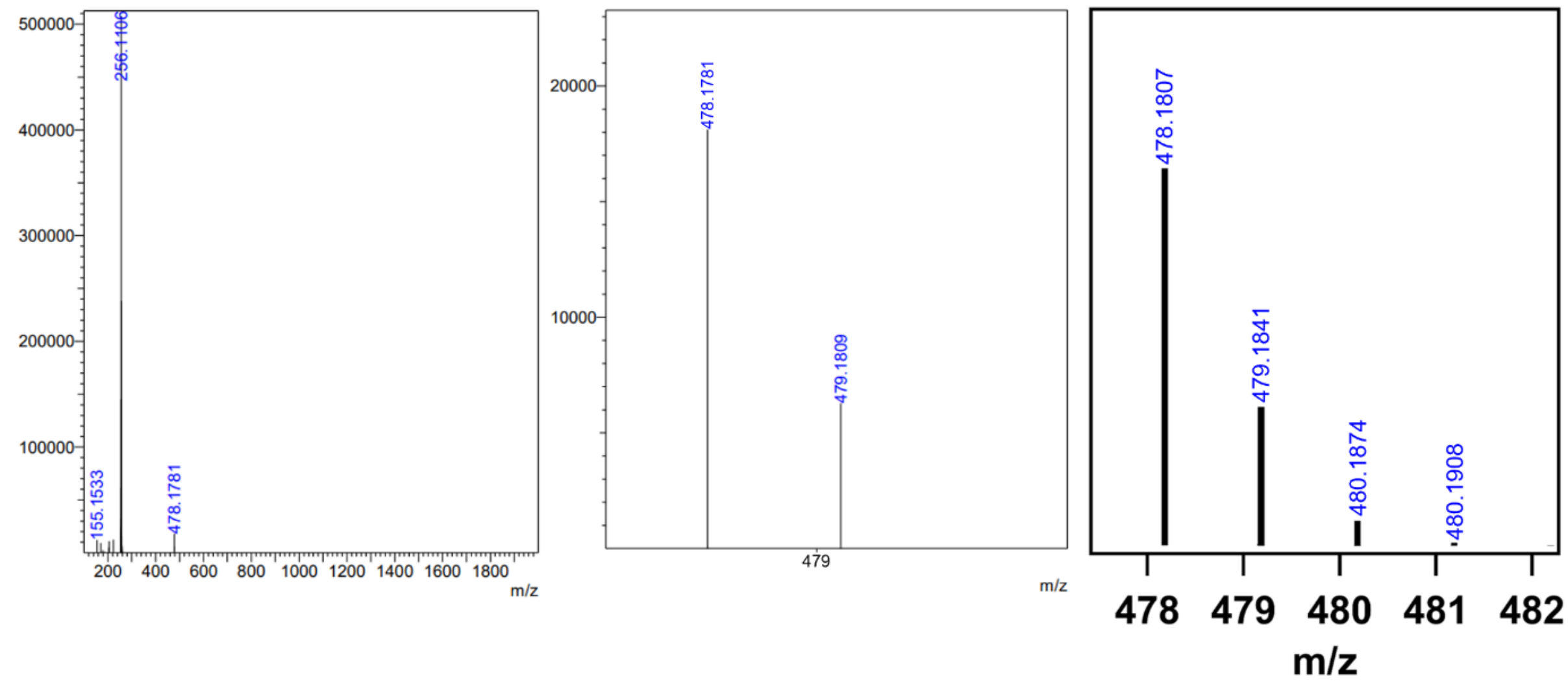

Figure S22. ESI-MS(+ve) spectrum of **2** (+H+) (found: left, middle; calculated: right).

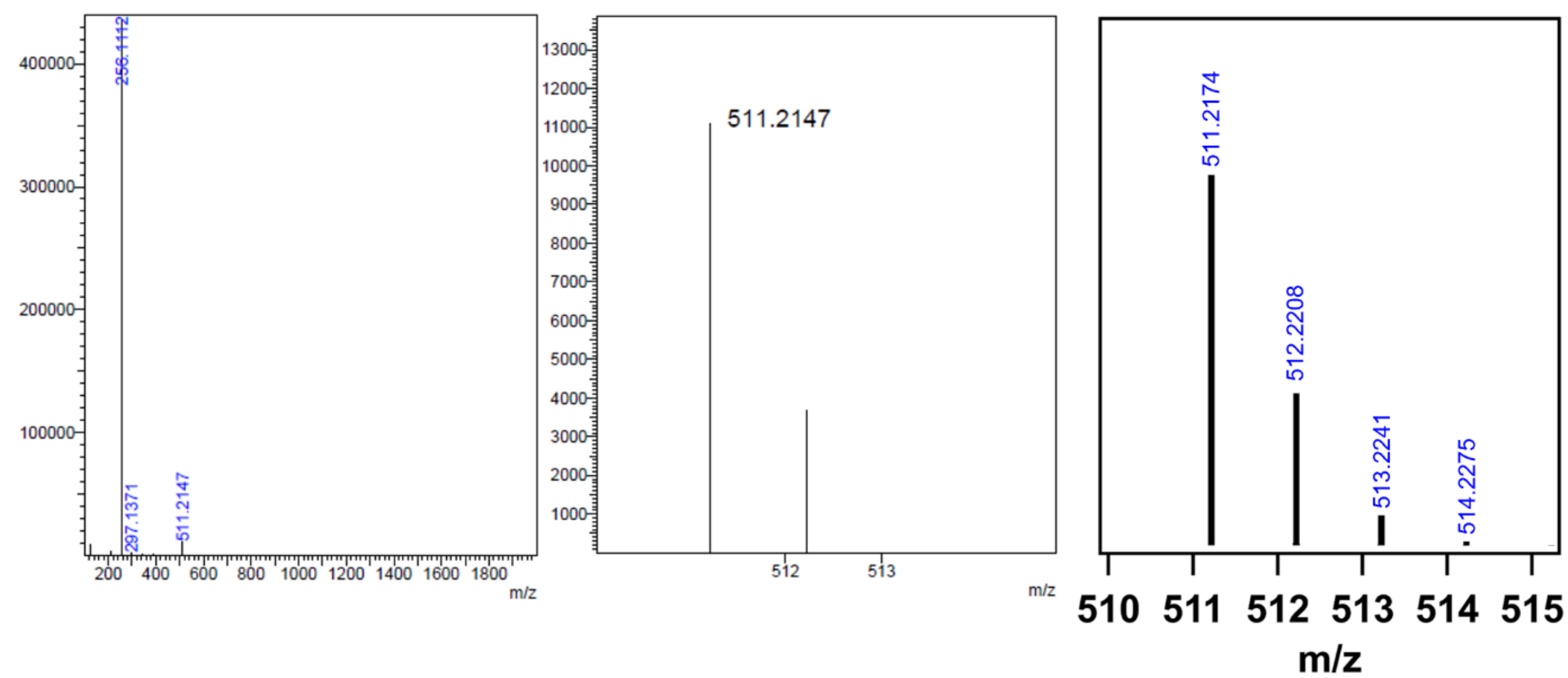

Figure S23. ESI-MS spectrum of 3 (+H+) (found: left, middle; calculated: right).

D. X-Ray Diffraction and Refinements

Table S2. Crystallographic data for **1**, **1-Dimer**, **2**, and **3**.

	1	1-Dimer	2	3
CCDC Accession Code	2563067	2563068	2563069	2563070
Chemical formula	$C_{34}H_{23}NO_2$	$C_{34}H_{23}NO_2$	$C_{45}H_{39}NO_3$	$C_{38}H_{26}N_2$
Formula weight	477.53	477.53	641.77	510.61
Space group	P1	P1	P1	C2/c
a (Å)	7.9351(3)	7.9110(3)	9.2095(10)	15.8072(5)
b (Å)	7.9780(3)	8.9083(4)	9.4874(7)	8.0588(2)
c (Å)	9.9791(2)	9.1741(3)	9.5396(6)	19.5291(5)
α (°)	81.079(2)	85.215(3)	90.681(5)	90
β (°)	88.750(2)	77.334(3)	91.131(7)	94.186
γ (°)	70.177(3)	66.310(4)	96.339(8)	90
V (Å3)	586.79(4)	577.63(4)	828.18(12)	2481.11(12)
Z	1	1	1	4
μ (mm^{-1})	0.083	0.085	0.080	0.609
T (K)	100.00(10)	100.00(10)	100.00(10)	293(2)
GOF (S) [all data]	1.095	1.056	1.555	1.055
R1[a] (wR2)[b] [I>2σ(I)]	0.0422 (0.1228)	0.0492 (0.1292)	0.1094 (0.3222)	0.0468 (0.1095)
Reflections	18706	17329	11341	18800
Radiation type (λ)	Mo kα (0.71073 Å)	Mo kα (0.71073 Å)	Mo kα (0.71073 Å)	Cu kα (1.54184 Å)

Table S3. Crystallographic data for solutions of **1** and **1-Dimer** in P-1 space group.

	1	1-Dimer
CCDC Accession Code		
Chemical formula	$C_{34.09}H_{23.07}N_{1.02}O_{1.95}$	$C_{34.21}H_{23.16}N_{1.051}O_{1.898}$
Formula weight	478.16	479.19
Space group	P-1	P-1
a (Å)	7.9351(3)	7.9110(3)
b (Å)	7.9780(3)	8.9083(4)
c (Å)	9.9791(2)	9.1741(3)
α (°)	81.079(2)	85.215(3)
β (°)	88.750(2)	77.334(3)
γ (°)	70.177(3)	66.310(4)
V (Å3)	586.79(4)	577.63(4)
Z	1	1
μ (mm^{-1})	0.083	0.085
T (K)	100.00(10)	100.00(10)
GOF (S) [all data]	1.092	1.082
R1[a] (wR2)[b] [I>2σ(I)]	0.0551 (0.1631)	0.0519 (0.1336)
Reflections	22345	17329

**Radiation type (λ)**	Mo kα (0.71073 Å)	Mo kα (0.71073 Å)

*Structural refinement of **1** in P1.*

Solution of the structure of **1** was initially prompted by the CrysAlisPro and Olex2 programs to be performed in the P-1 space group. While this does allow for accurate structural determination and differentiation of the COOH and Py pendant groups' bond parameters and overall 1:1 stoichiometry (Figures SX and SX), it fails to account for the positional disorder inherent to the 1D chain secondary structure, which can tolerate four orientations of the two groups in both mixed functionality pairs (i.e. Py-COOH and COOH-Py), as well as homoleptic pairs (i.e. Py-Py, COOH-COOH). Thus, to account for all possible combinations in the structure, we solved the structure in P1, which found two An molecules in the asymmetric unit: one $\mathbf{An}^{\mathbf{COOH}}$ and one $\mathbf{An}^{\mathbf{Py}}$. However, the $\mathbf{An}^{\mathbf{COOH}}$ molecule featured residual electron density corresponding to the N, and 2,6-C's of a Py moiety. The 3,5 C's were not resolvable in a different position from the apparently dominant COOH functionality in this position, leading us to refine partial occupancy of these C-atoms at the same position as the O-atoms. The COOH of this molecule was assigned to Part 1 and the minor-occupancy Py moiety was assigned to Part 2. Similarly, the 3,5-C's of the Py-dominated side were refined with partial occupancy as a COOH-moiety in Part 2 of the disorder model, with the original Py species in Part 1. The 4-C of the Py and C-atom of the carboxylate were refined with 100% occupancy in Part 0, as this C is present in both functionalized Ans. The Part 1 and 2 occupancies were refined with free variables to add to a total occupancy of 1, resulting in a 0.654:0.346 ratio between Part 1 and 2, respectively. These occupancies allow us to determine the relative percentages of packing defects which produce heterodimer **2** and homodimers **3** and **4** in the SCSC transformation. See Figures SX and 2c in the manuscript for a pictorial description of the disorder model.

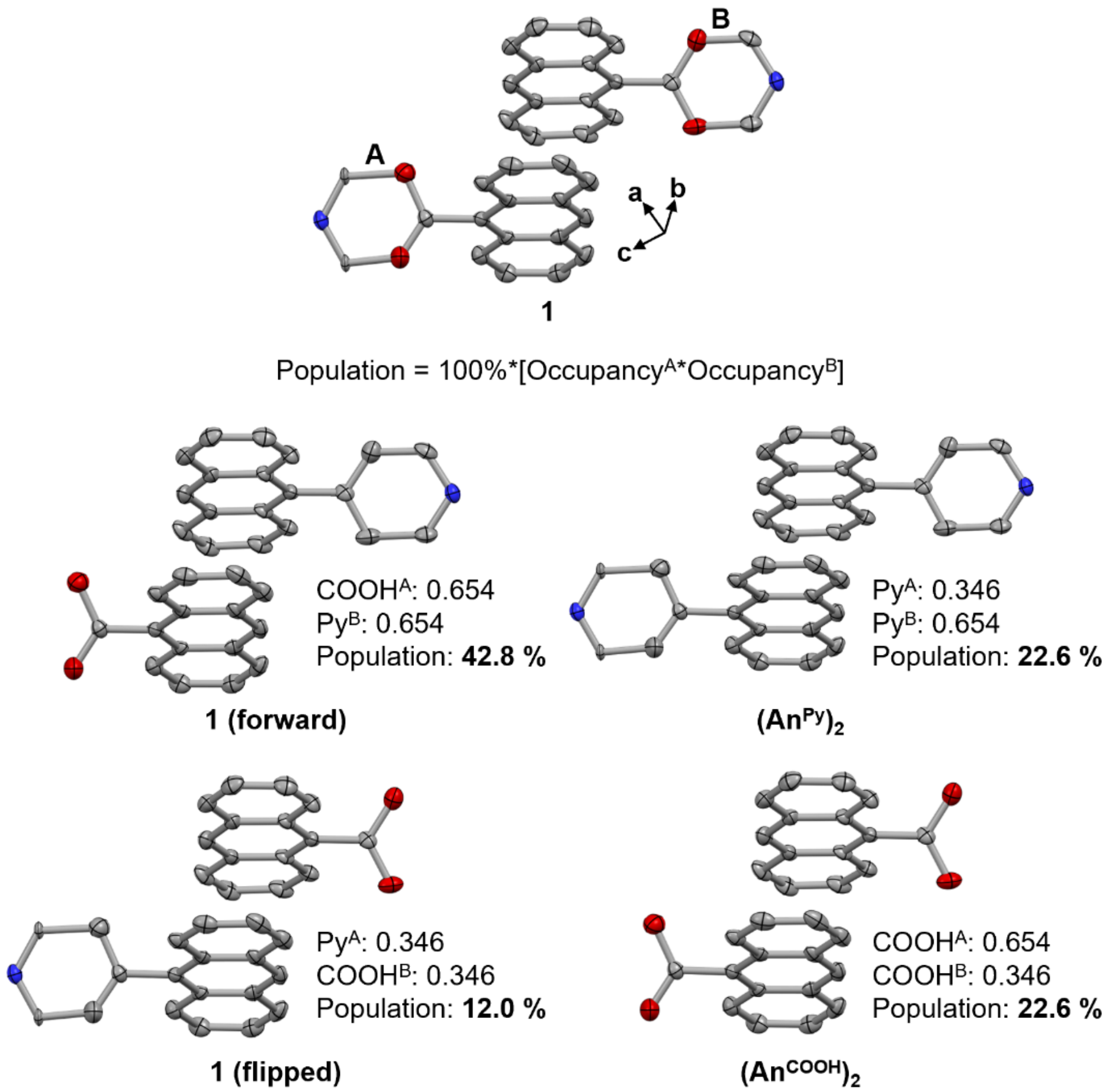

Component	Population
**An^{COOH}/An^{Py} (forward + flipped)**	42.8 + 12.0 = 54.8 %
**$(An^{Py})_2$**	22.6 %
**$(An^{COOH})_2$**	22.6 %

Figure S24. Molecular structure of 1 shown with 50% probability ellipsoids and relative populations of molecular packing directions and pairings calculated from the optimized disorder model in the P1 space group. H-atoms removed for clarity. Color code C: grey; O: red; N: blue.

*Structural refinement of **1-Dimer** in P1.*

An identical disorder model process as **1** was employed in **1-Dimer**, producing a 0.631:0.369 ratio of functional group occupancies. The products of these ratios allows for determination of hetero- and homodimers in the crystal (Figures S25, 2c, e).

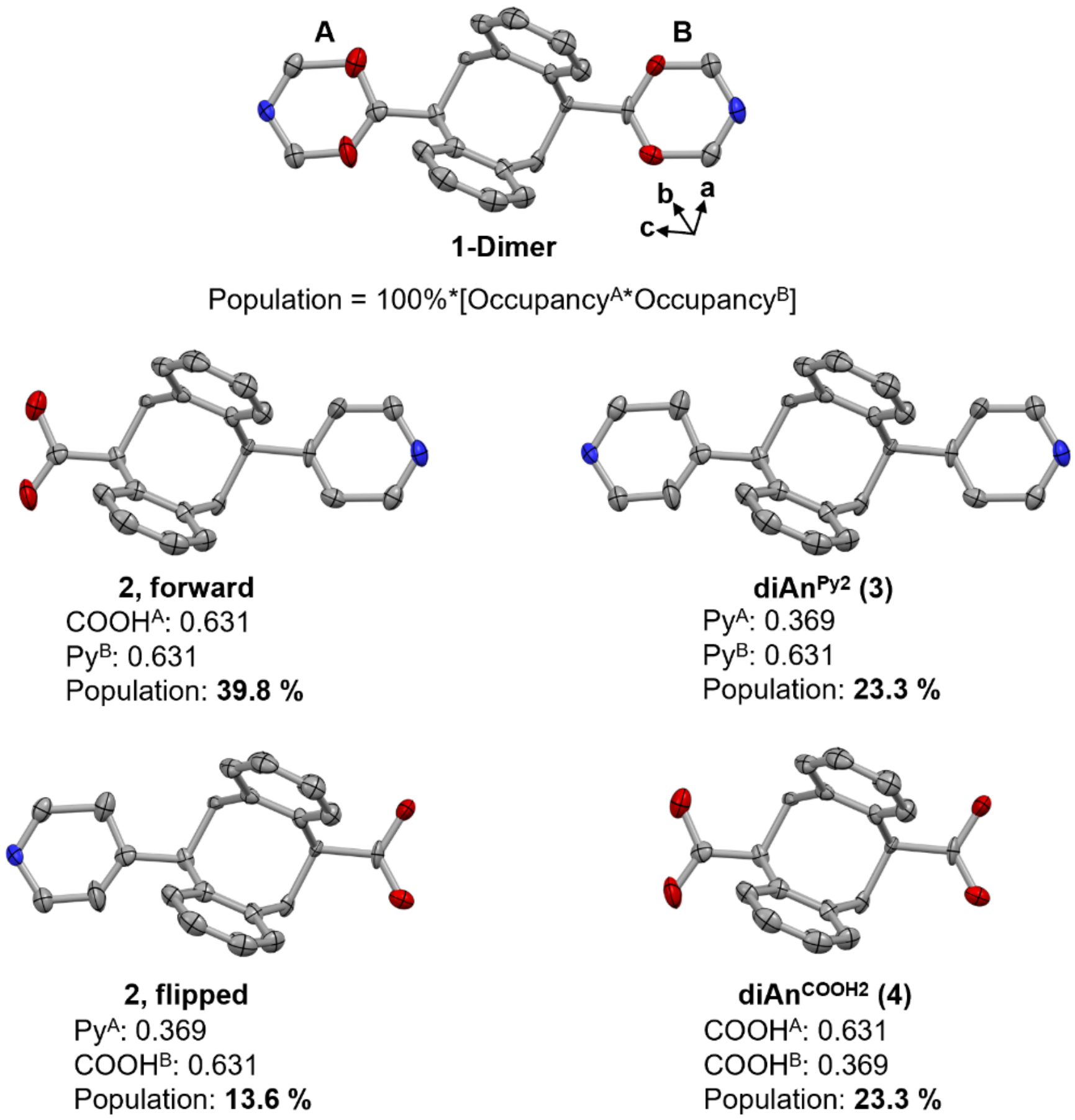

Component	Population
**2 (forward + flipped)**	39.8 + 13.6 = 53.4 %
**3**	23.3 %
**4**	23.3 %

Figure S25. Molecular structure of 1 shown with 50% probability ellipsoids and relative populations of component dimers **2**, **3**, and **4** and molecular orientations calculated from the optimized disorder model in the P1 space group. H-atoms removed for clarity. Color code C: grey; O: red; N: blue.

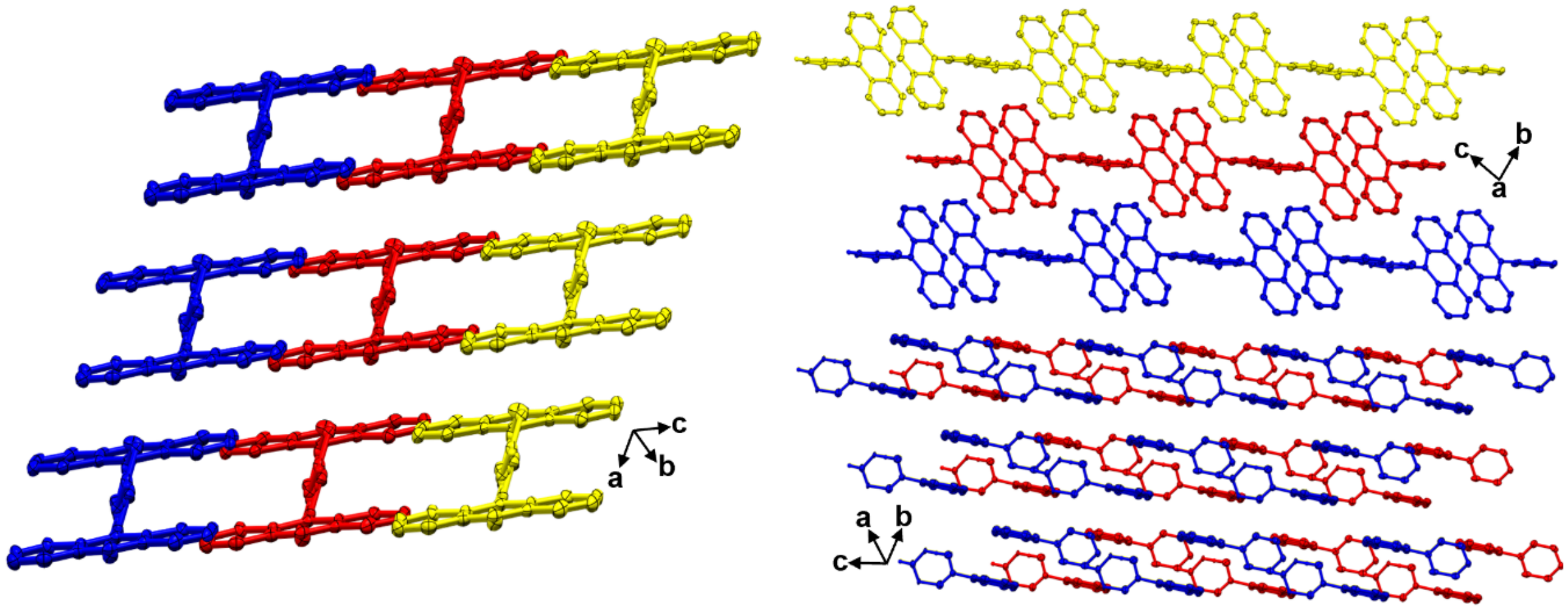

Figure S26. Crystal packing of **1** from different points of view.

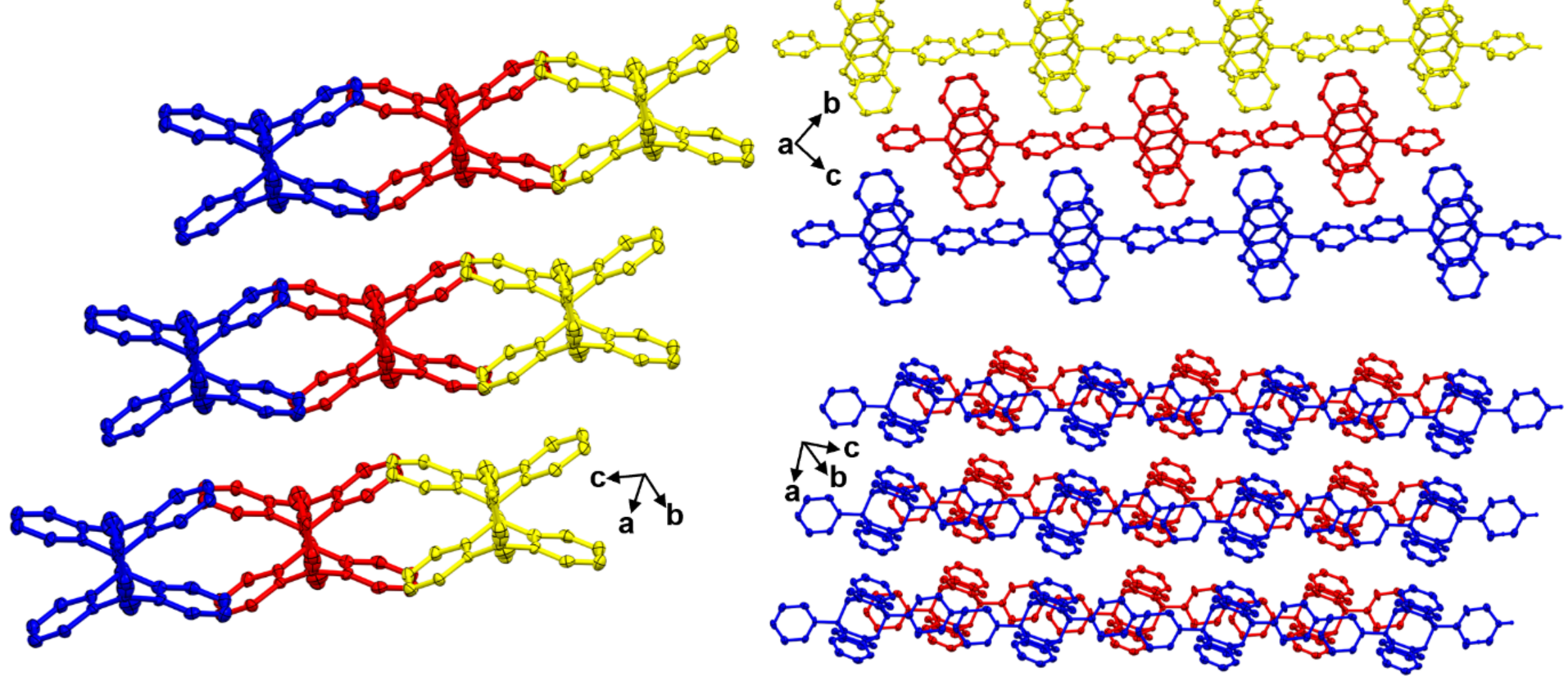

Figure S27. Crystal packing of **1-Dimer** from three different points of view.

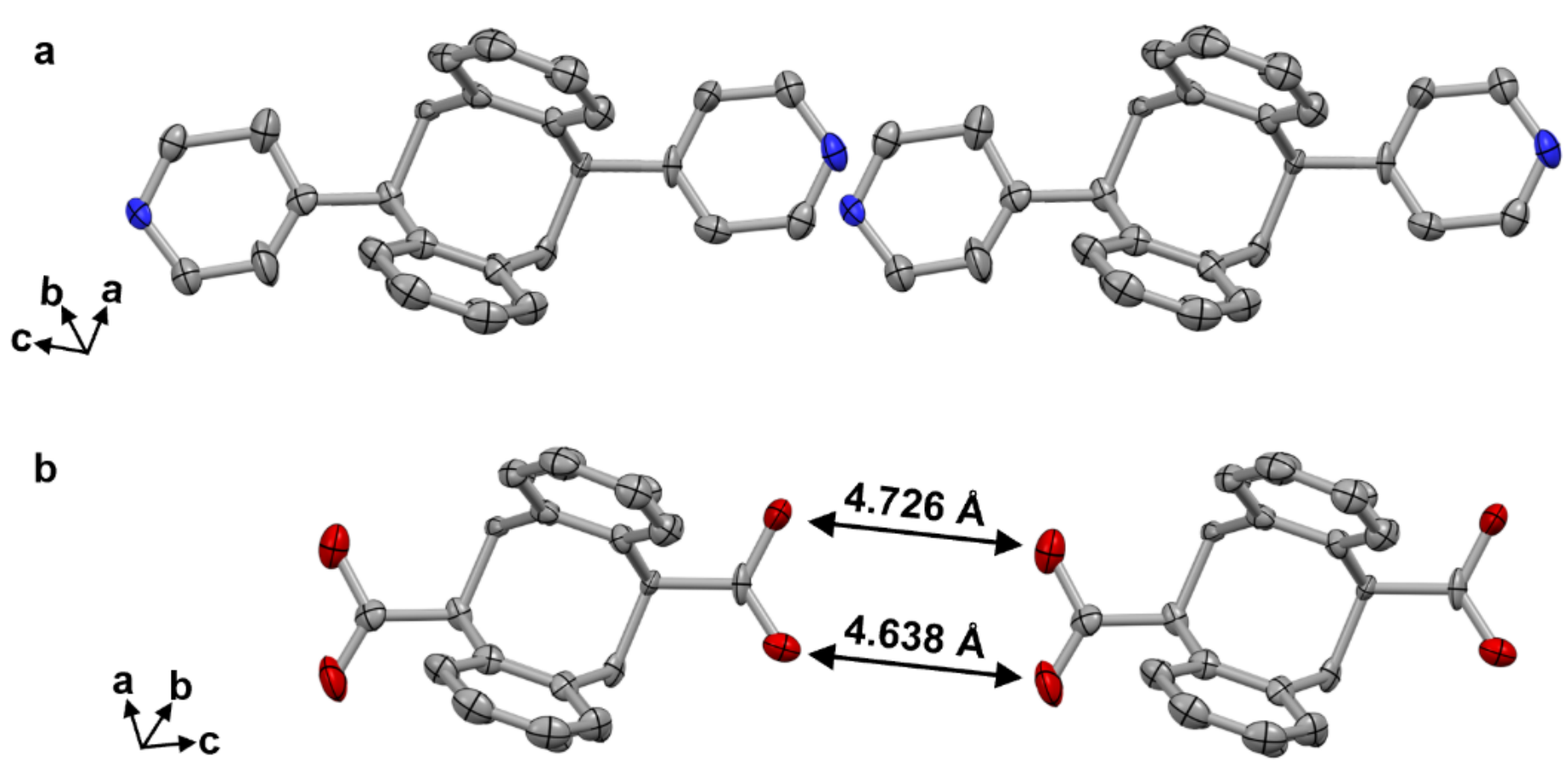

Figure S28. a) Demonstration of steric crowding of a theoretical Py-Py pair in the crystal lattice of **1-Dimer**. b) Demonstration of the long distance between carboxylate groups in a COOH-COOH pair in **1-Dimer**. Color code C: grey; O: red; N: blue.

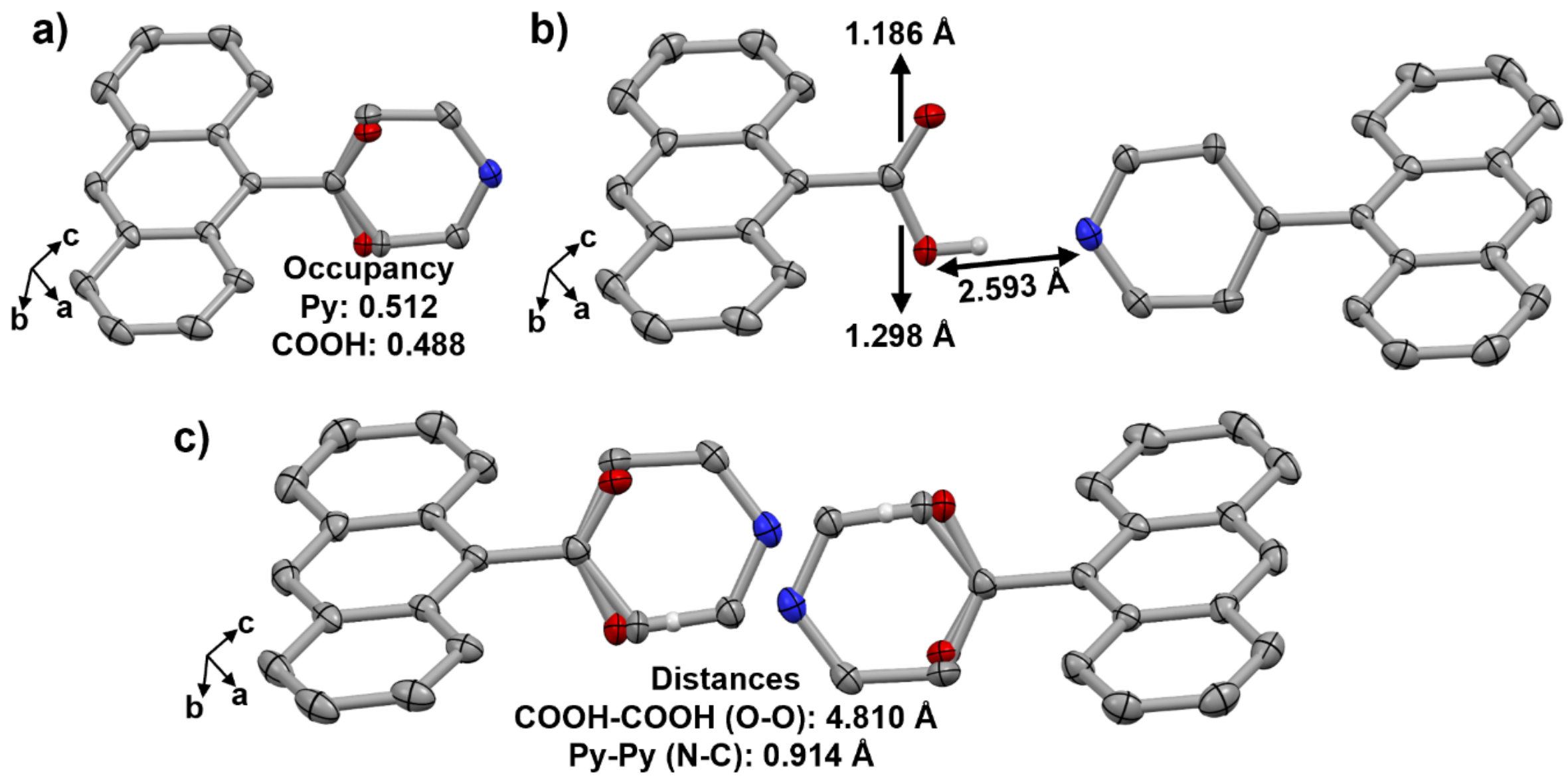

Figure S29. a) Molecular structure of **1** solved in the P-1 space group shown with 50% probability ellipsoids with Py and COOH occupancies. H-atoms removed for clarity. b) H-bonding between 9-functionalized Ans and selected bond distances. c) Distances between homoleptic 9-RAn pairs (i.e. COOH-COOH, Py-Py).

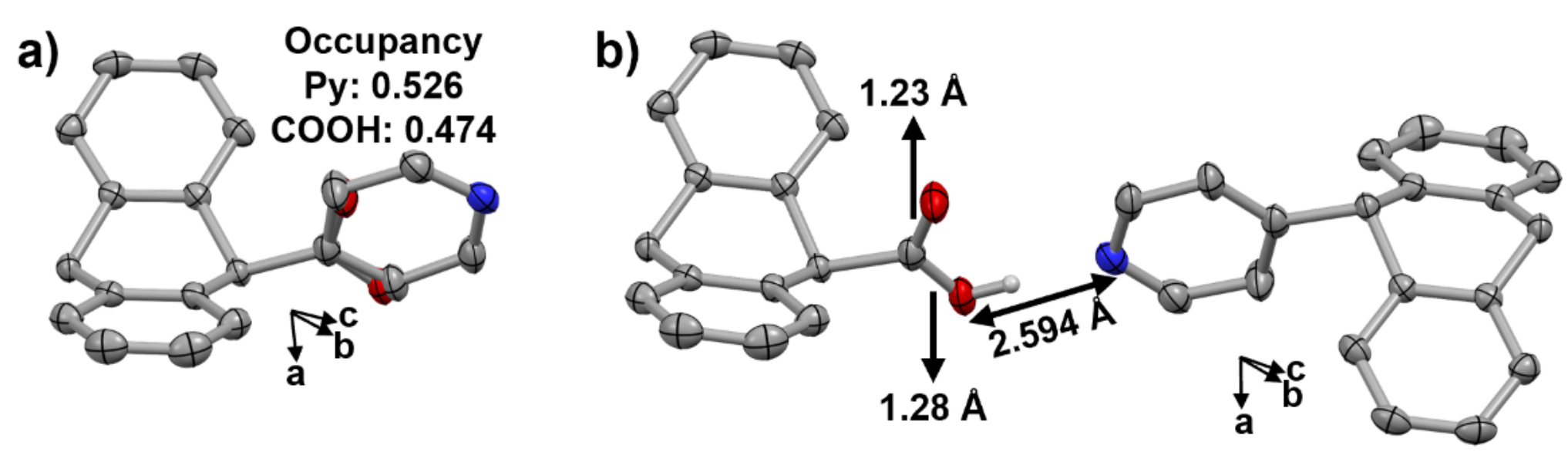

Figure S30. a) Molecular structure of **1-Dimer** solved in the P-1 space group shown with 50% probability ellipsoids with Py and COOH occupancies. H-atoms removed for clarity. b) H-bonding between 9-functionalized Ans and selected bond distances.

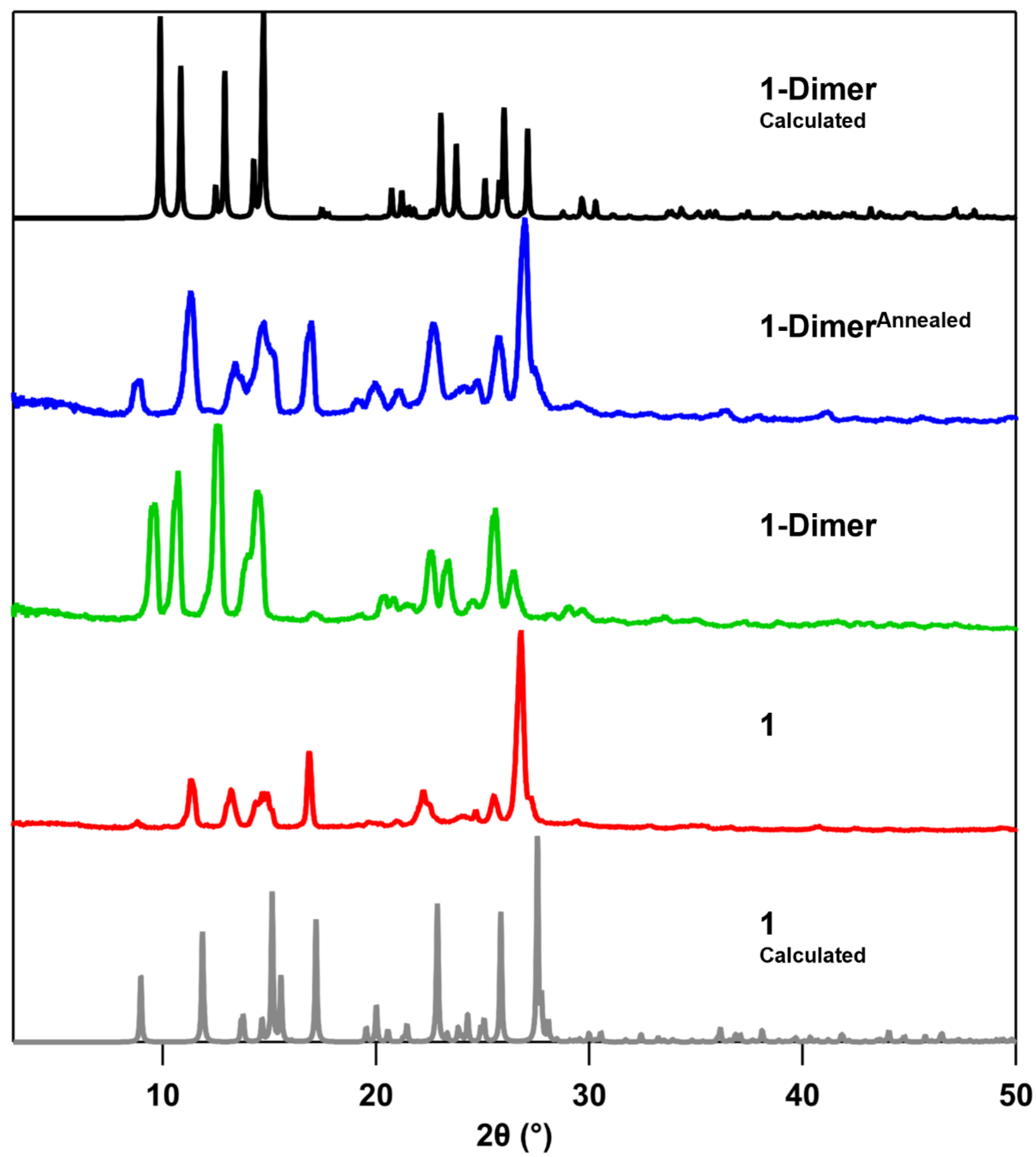

Figure S31. PXRD patterns of bulk synthesis and retrocyclization of **1-Dimer**, with calculated patterns determined from SCXRD.

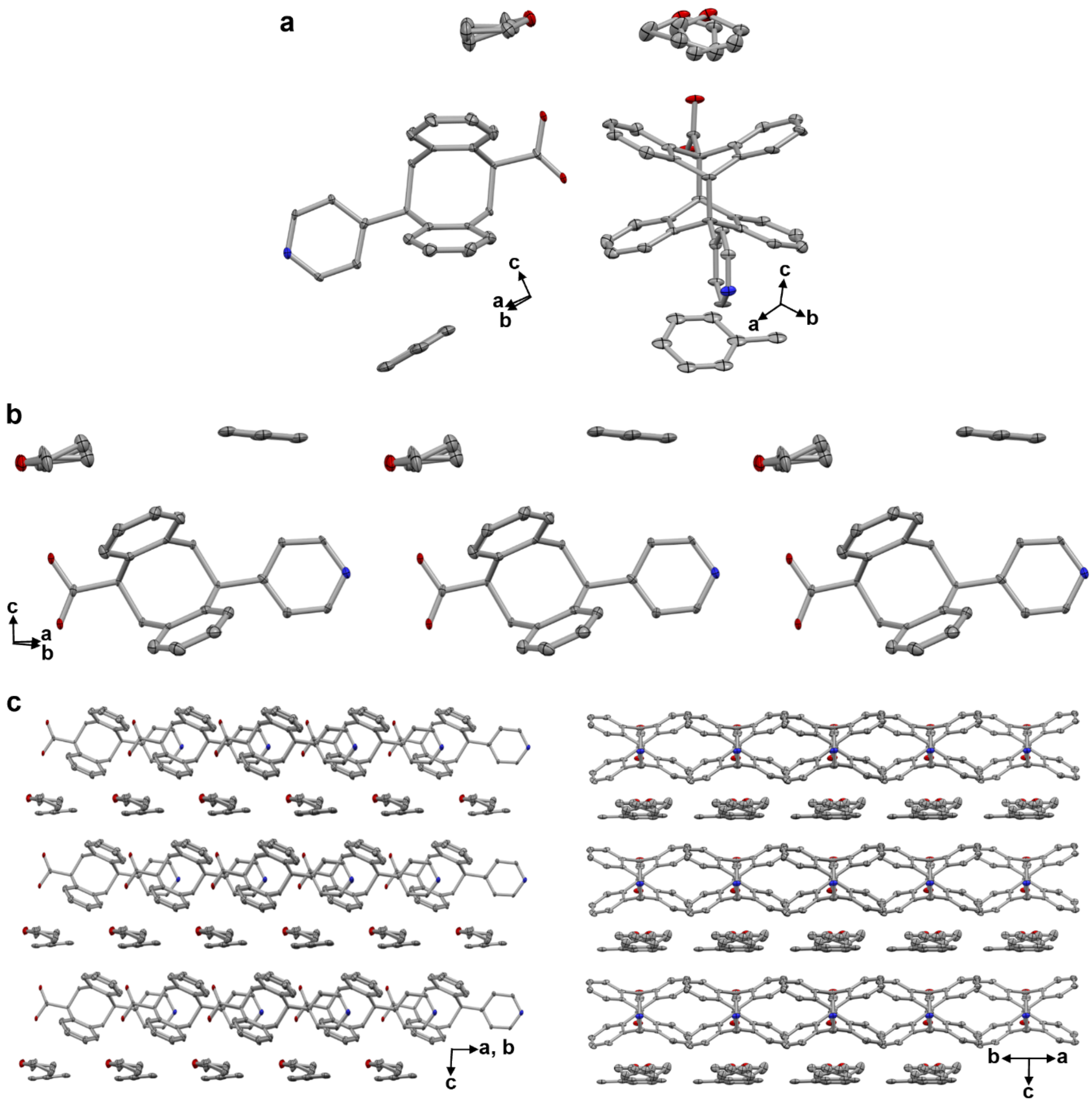

Figure S32. Molecular structure of **2** shown with 50% probability ellipsoids. a) Asymmetric unit from two perspectives. b) 1D chain packing. c) Bulk crystal packing from two perspectives. H-atoms removed for clarity. Color code C: grey; O: red; N: blue.

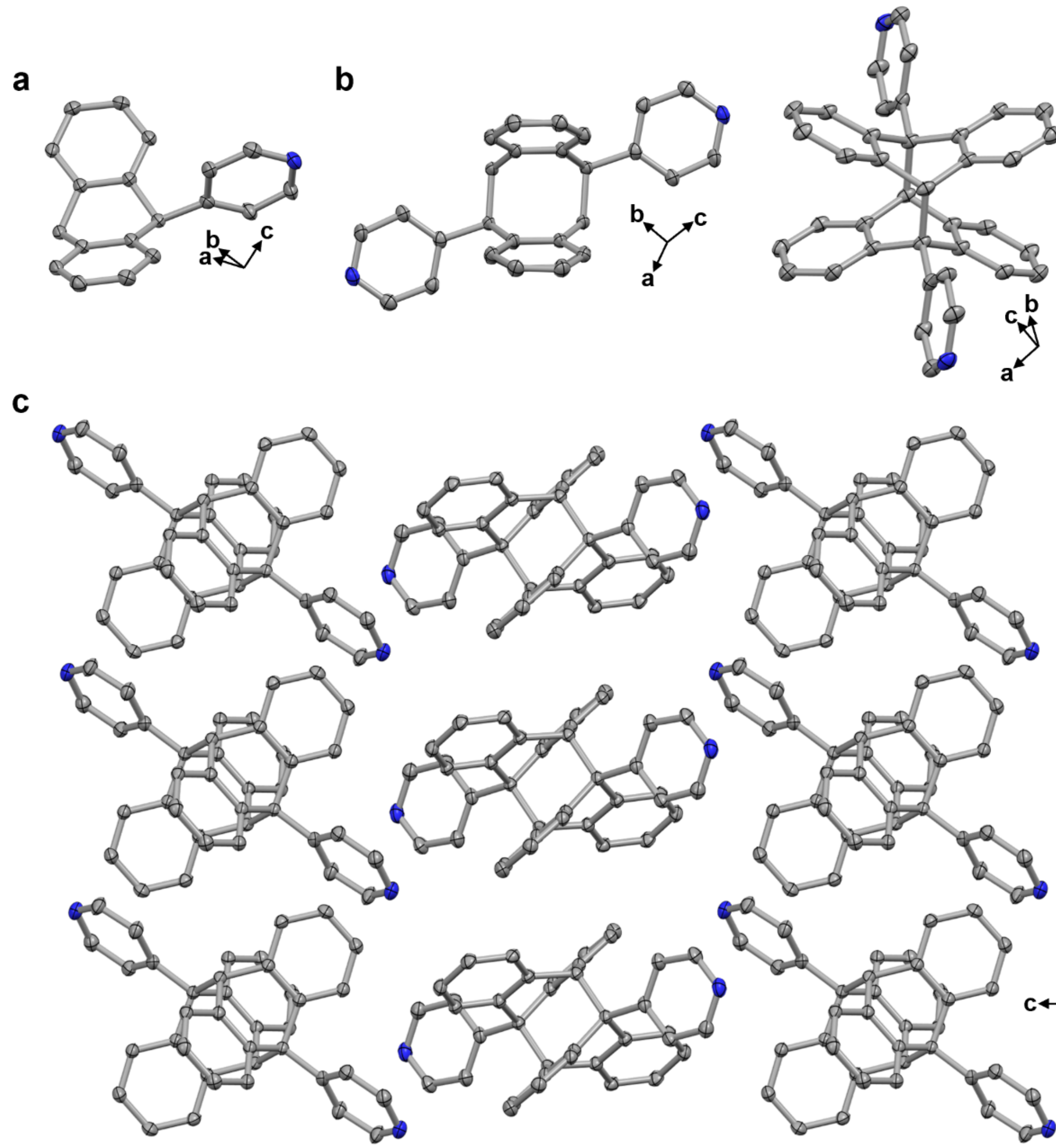

Figure S33. Molecular structure of **3**. a) Asymmetric unit. b) Full molecular structure from two perspectives. c) Bulk crystal packing. H-atoms removed for clarity. Color code C: grey; O: red; N: blue.

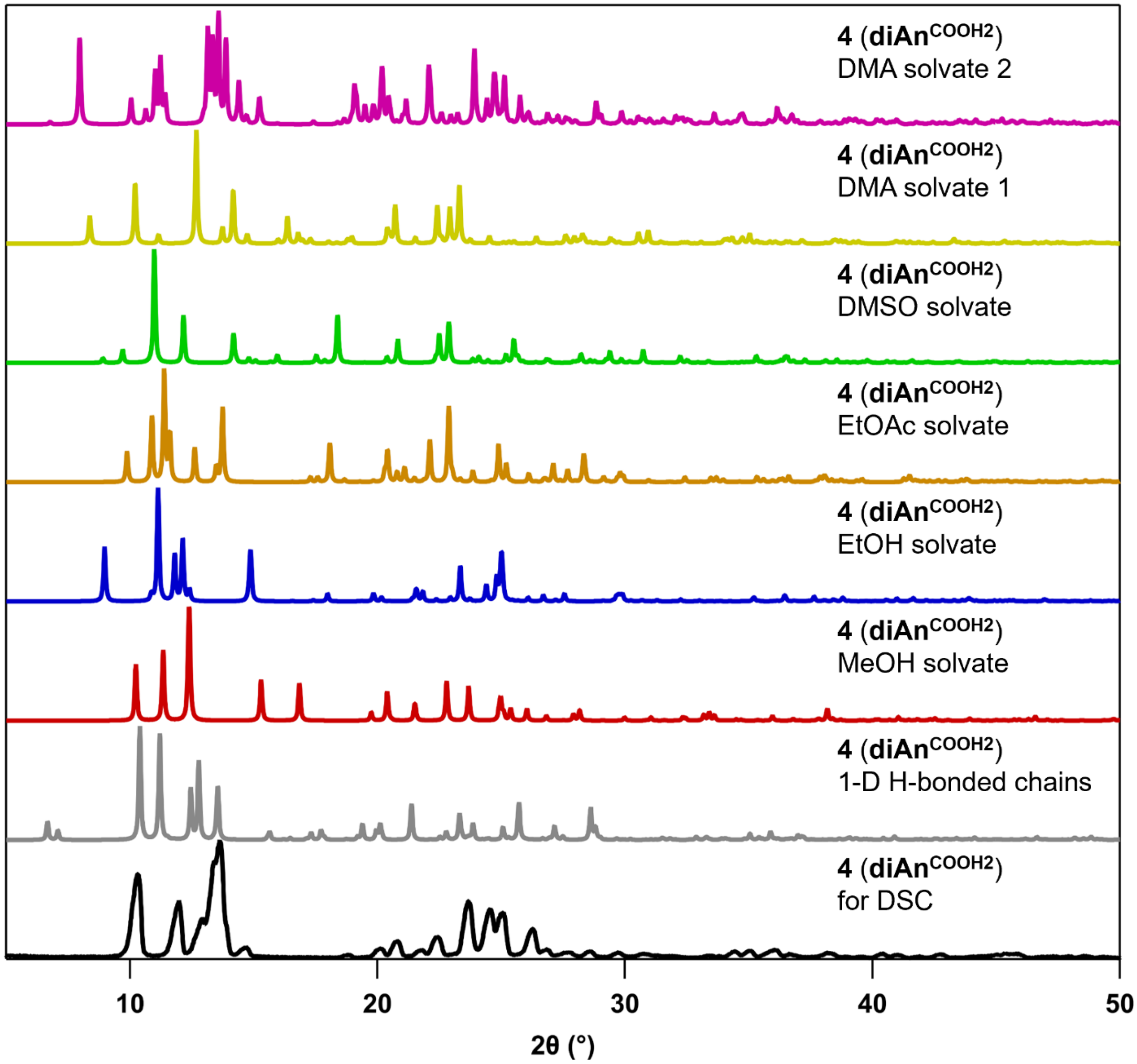

Figure S34. PXRD pattern of **4** used here and calculated patterns from 7 reported SCXRD structures.[10, 11]

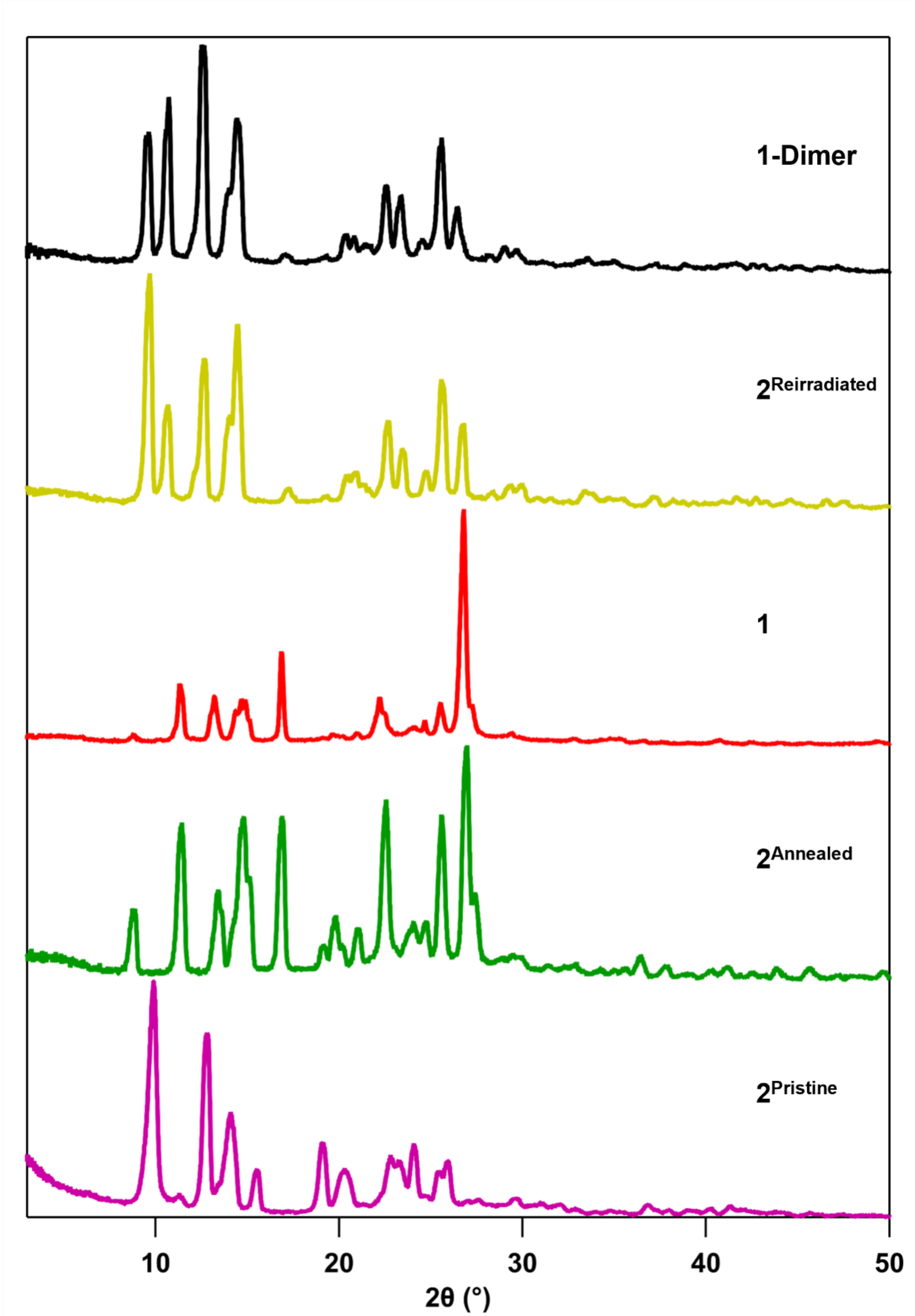

Figure S35. PXRD of **2** before and after C-D cycling for one cycle. Upon recharging, the pattern matches that of the disordered heterodimer, **1-Dimer**.

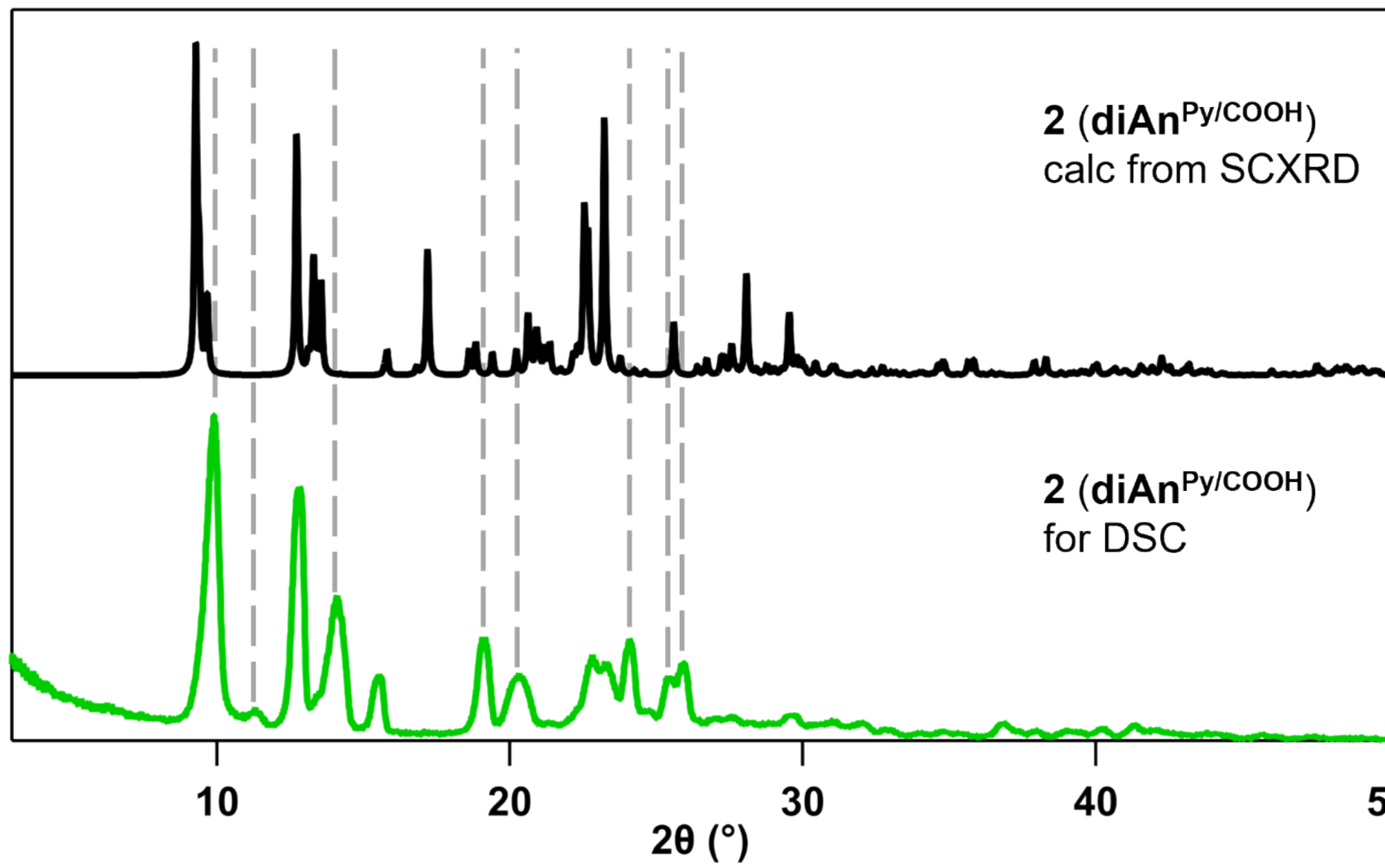

Figure S36. PXRD of **2** as-isolated from column chromatography and used in DSC and cycling experiments (green, bottom) and the PXRD pattern calculated from the single crystal X-ray structure of **2**, which is a THF and toluene solvate.

E. Microcrystal Electron Diffraction (MicroED)

Table S4. Progression of unit cell parameters for crystal 1 of **1-Dimer** upon electron beam dosage.

	Crystal 1					
e-beam dose ($e^-/Å^2$)	a (Å)	b (Å)	c (Å)	α	β	γ
0.662	7.974	8.71	9.305	87.42	78.9	67.1
1.324	8.028	8.498	9.315	86.36	78.14	68.01
0.986	8.067	8.367	9.434	86.61	78.4	68.39
2.648	8.042	8.243	9.49	86.96	79.16	68.88
3.310	7.992	8.195	9.598	87.27	79.45	69.18
3.972	7.945	8.184	9.664	87.69	79.87	69.32
4.634	7.862	8.189	9.804	89.28	80.92	69.71
5.296	7.851	8.170	9.737	88.25	80.49	69.62
5.958	7.834	8.165	9.74	88.12	80.49	69.79
6.620	7.804	8.179	9.817	89.01	80.83	69.93
7.282	7.784	8.189	9.891	89.52	81.03	70.08
7.944	7.796	8.144	9.815	88.6	80.82	70.0
8.606	7.778	8.167	9.834	88.89	80.88	70.0
9.268	7.782	8.171	9.824	88.9	80.89	69.99

Table S5. Progression of unit cell parameters for crystal 2 of **1-Dimer** upon electron beam dosage.

	Crystal 2					
e-beam dose ($e^-/Å^2$)	a (Å)	b (Å)	c (Å)	α	β	γ
0.662	8.029	8.716	9.336	85.47	76.76	66.54
1.324	8.058	8.526	9.393	85.52	77.39	67.33
0.986	8.062	8.329	9.44	88.25	78.25	68.07
2.648	8.027	8.226	9.518	86.91	79.05	68.54
3.310	7.964	8.183	9.618	87.41	79.56	68.86
3.972	7.897	8.154	9.669	87.72	80.07	69.13
4.634	7.884	8.138	9.729	87.91	80.13	69.29
5.296	7.848	8.141	9.759	88.17	80.39	64.43
5.958	7.832	8.138	9.786	88.32	80.42	69.42
6.620	7.814	8.139	9.805	88.39	80.59	69.52
7.282	7.808	8.143	9.814	88.39	80.61	69.49
7.944	7.791	8.134	9.848	88.54	80.63	69.63
8.606	7.794	8.141	9.841	88.58	80.64	69.63
9.268	7.786	8.14	9.843	88.55	80.69	69.64

9.9330	7.783	8.137	9.846	88.66	80.71	69.72
10.592	7.783	8.136	9.852	88.57	80.68	69.68
11.254	7.783	8.138	9.862	88.56	80.62	69.69
11.916	7.779	8.138	9.857	88.57	80.67	69.71
12.578	7.776	8.141	9.851	88.59	80.76	69.69
13.240	7.781	8.146	9.845	88.37	80.73	69.58

Table S6. Progression of unit cell parameters for crystal 3 of **1-Dimer** upon electron beam dosage.

	Crystal 3					
e-beam dose (e^-/Å^2)	a (Å)	b (Å)	c (Å)	α	β	γ
0.662	7.956	8.604	9.198	85.32	77.39	67.29
1.324	8.035	8.356	9.328	85.47	77.6	68.12
0.986	8.159	8.159	9.473	85.84	78.11	68.33
2.648	8.159	8.06	9.593	86.37	78.45	68.87
3.310	8.136	8.027	9.676	87.02	79.03	69.01
3.972	8.1	8.026	9.731	87.38	79.43	69.11
4.634	8.049	8.017	9.775	87.73	79.72	69.31
5.296	8.018	8.004	9.8	87.91	79.97	69.47
5.958	7.987	7.997	9.83	88.13	80.14	69.58
6.620	7.972	7.988	9.847	88.22	80.29	69.67
7.282	7.957	7.981	9.863	88.31	80.38	69.71
7.944	7.945	7.978	9.873	88.39	80.42	69.76
8.606	7.938	7.992	9.886	88.46	80.48	69.74
9.268	7.941	7.985	9.892	88.5	80.59	69.71
9.933	7.9279	7.97	9.894	88.48	80.538	69.85
10.592	7.928	7.989	9.9	88.57	80.62	69.8
11.254	7.923	7.98	9.902	88.53	80.63	69.75
11.916	7.919	7.977	9.907	88.55	80.67	69.78
12.578	7.914	7.976	9.908	88.56	80.64	69.86
13.240	7.918	7.979	9.909	88.56	80.65	69.8

Table S7. Crystallographic data from combined MicroED of 3 crystals of **1-Dimer** post-e-beam irradiation.

	1-Dimer$^{e\text{-}beam}$
CCDC Accession Code	2594106
Chemical formula	$C_{34}H_{23}N_1O_2$
Formula weight	477.55
Space group	P1
a (Å)	7.840(3)
b (Å)	8.103(4)
c (Å)	9.8922(17)
α (°)	88.78(2)
β (°)	80.40(2)
γ (°)	69.90(3)
V (Å^3)	581.4(3)
Z	1
μ (mm^{-1})	0.000
T (K)	102
GOF (S) [all data]	1.208
R1[a] (wR2)[b] [I>2σ(I)]	0.2274 (0.5083)
Reflections	4556
Radiation type (λ)	Electron (0.0251)

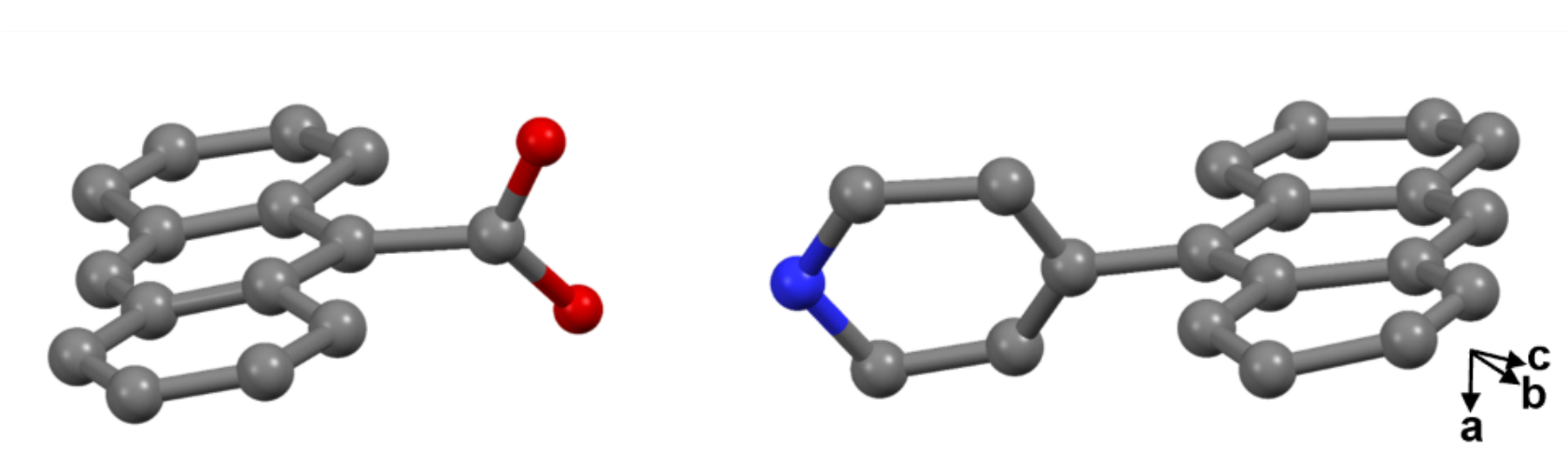

Figure S37. MicroED structure of **1-Dimer** after electron beam exposure shown as a ball-and stick rendering. This connectivity structure reveals reformation of monomer cocrystal **1**.

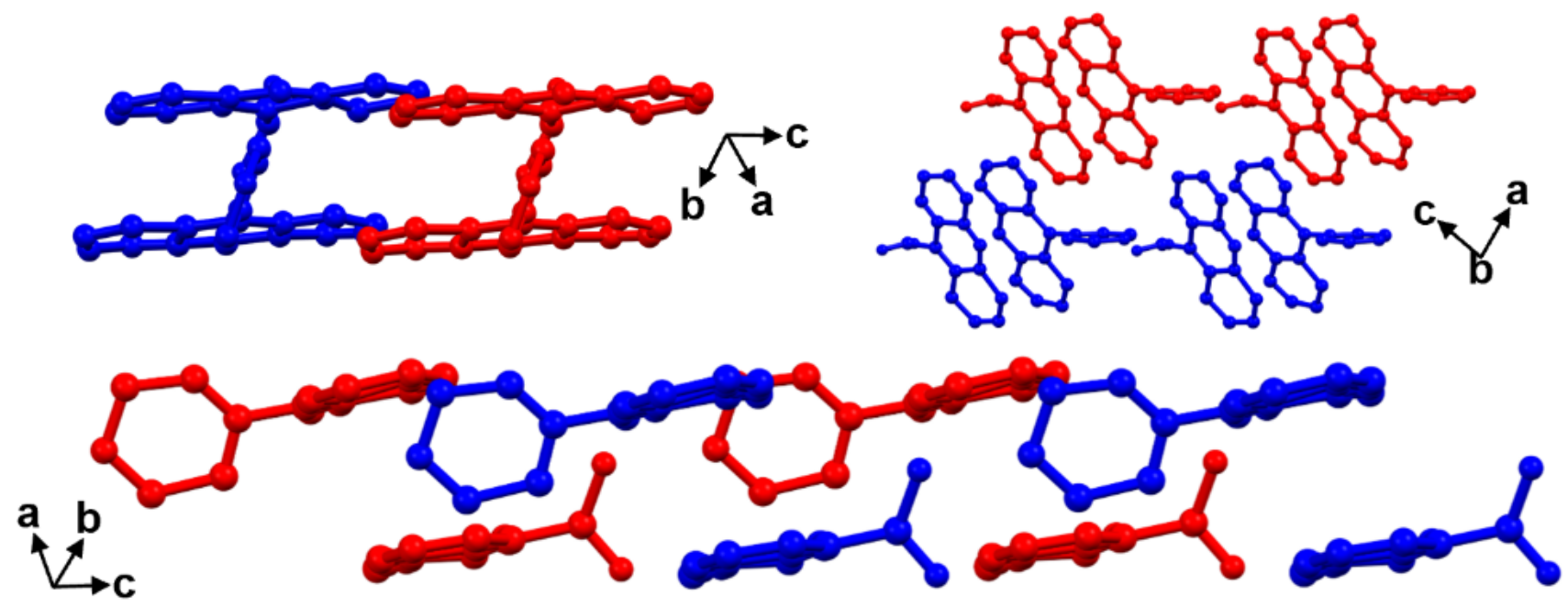

Figure S38. Crystal packing of the MicroED structure of **1** from e-beam irradiation of **1-Dimer** from different points of view.

F. Differential Scanning Calorimetry

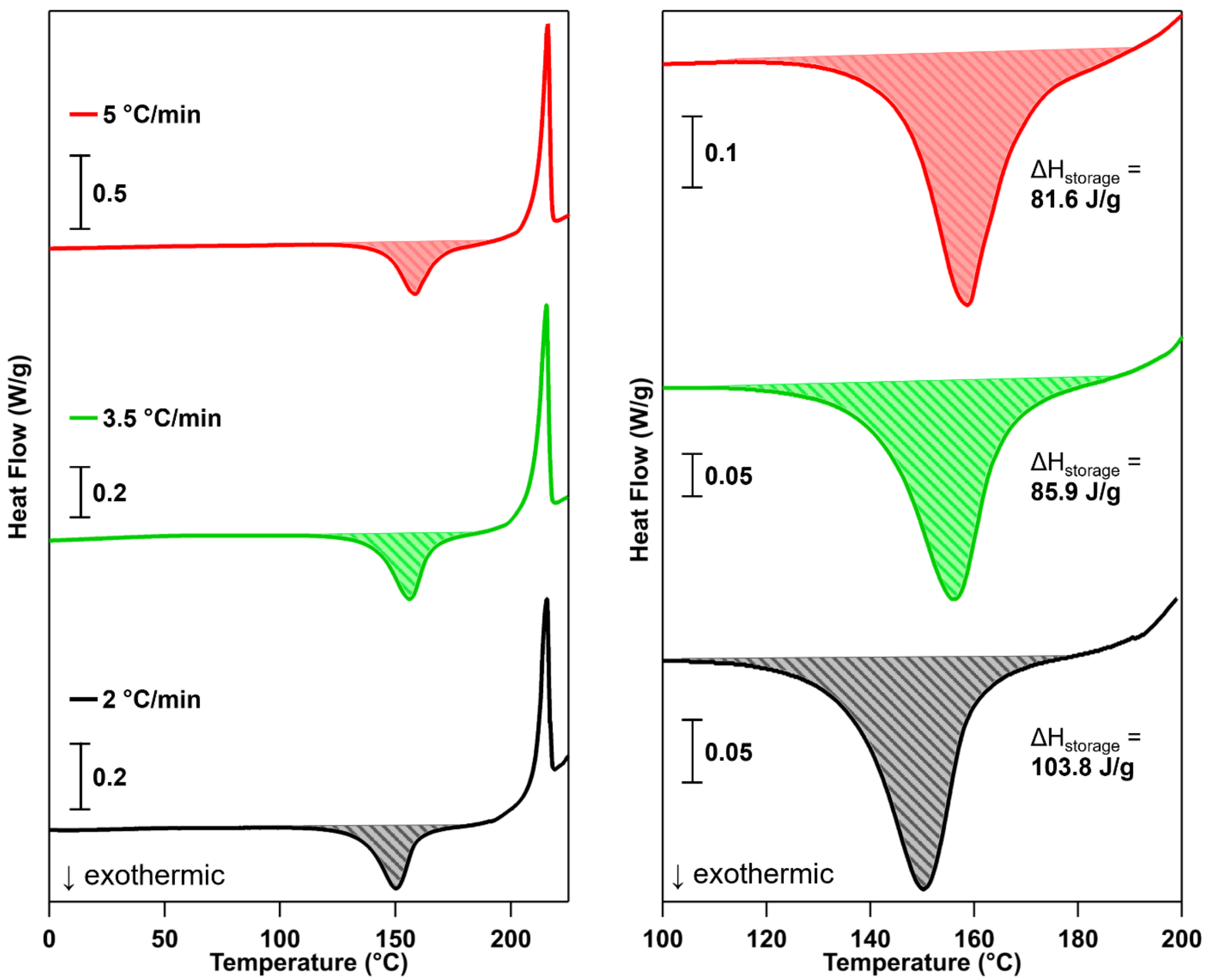

Figure S39. Differential scanning calorimetry traces of **1-Dimer** recorded at 2, 3.5, and 5 °C/min with $\Delta H_{storage}$ values.

G. Diffuse Reflectance Spectroscopy

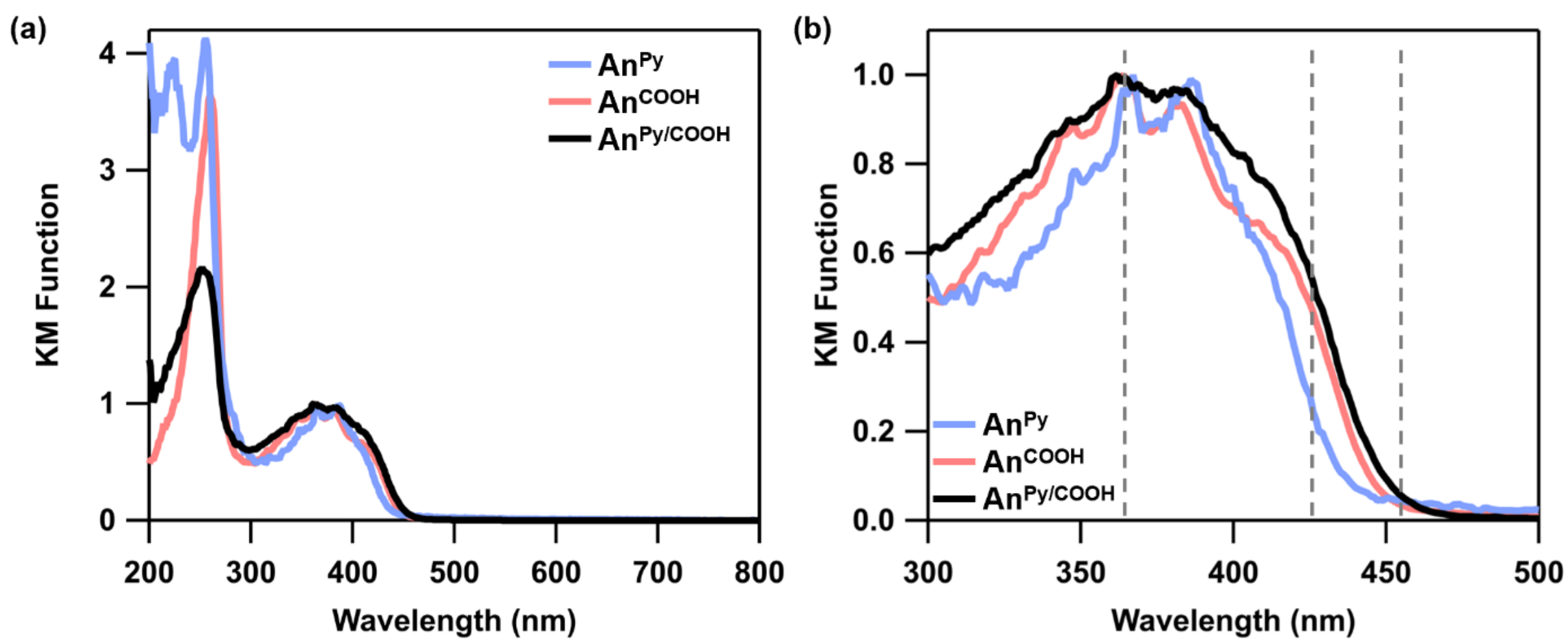

Figure S40. Diffuse reflectance spectra of $\mathbf{An^{Py}}$, $\mathbf{An^{COOH}}$ and $An^{Py/COOH}$ (**1**) at 25 °C (a) between 200-800 nm and (b) between 300-500 nm, highlighting the relative absorptivity of each species at 365, 427, and 456 nm. Spectra are normalized to the most intense band between 300-450 nm.

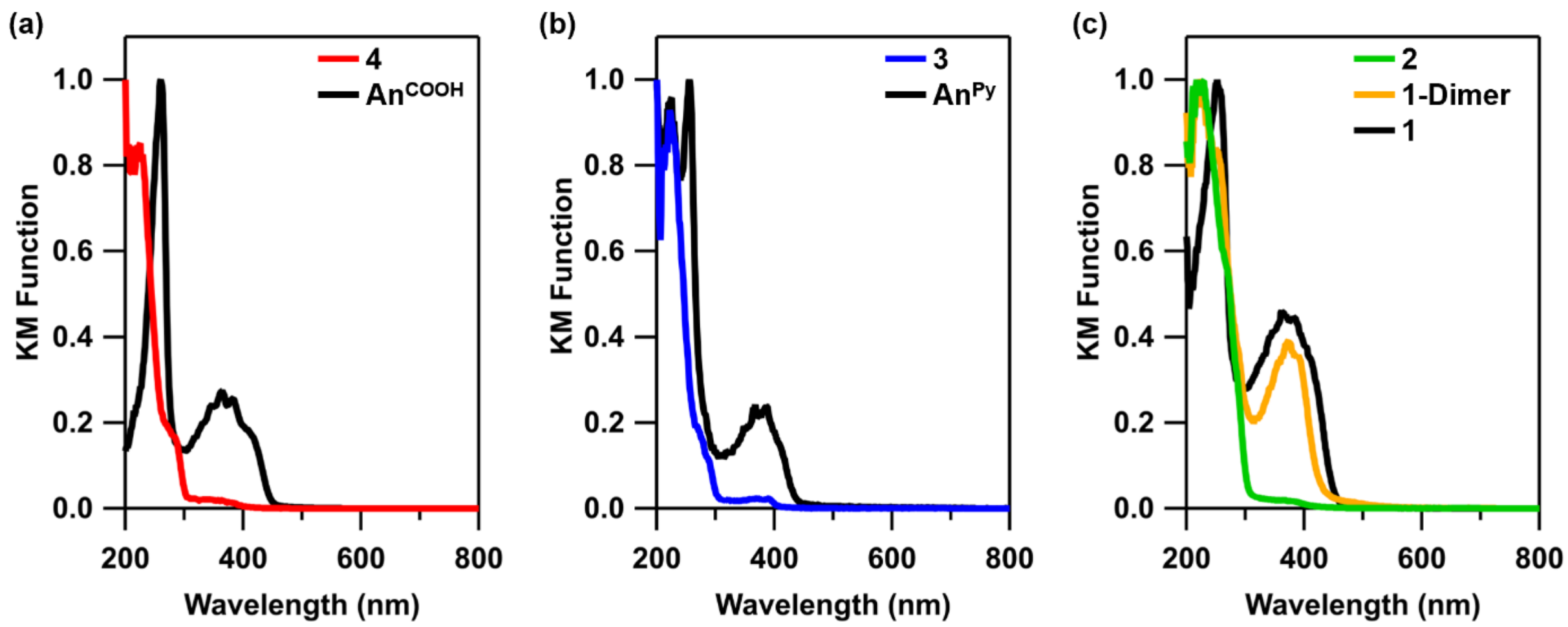

Figure S41. Diffuse reflectance spectra of a) **An^{COOH}**, **4**, b) **An^{Py}**, **3**, c) **1**, **1-Dimer**, and **2** at 25 °C. Spectra are normalized to the most intense band between 200-300 nm.

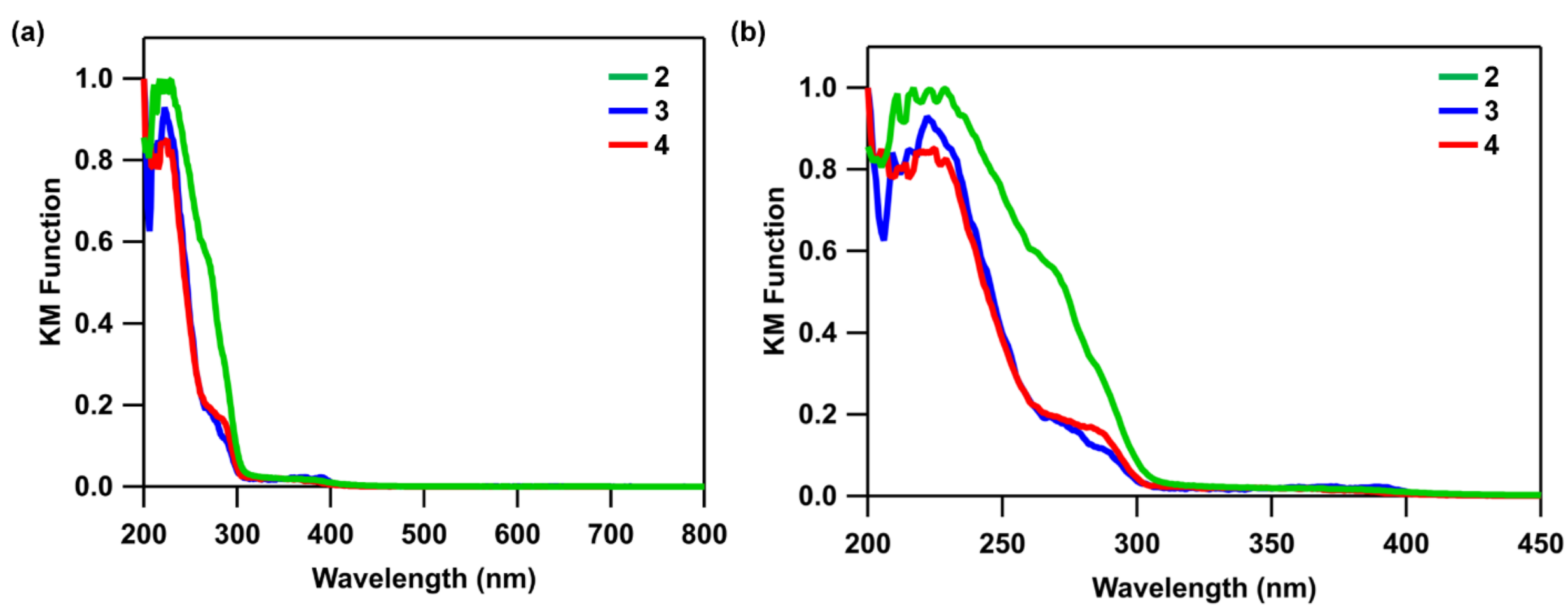

Figure S42. Diffuse reflectance spectra of **2**, **3**, and **4**.

H. Microscopy

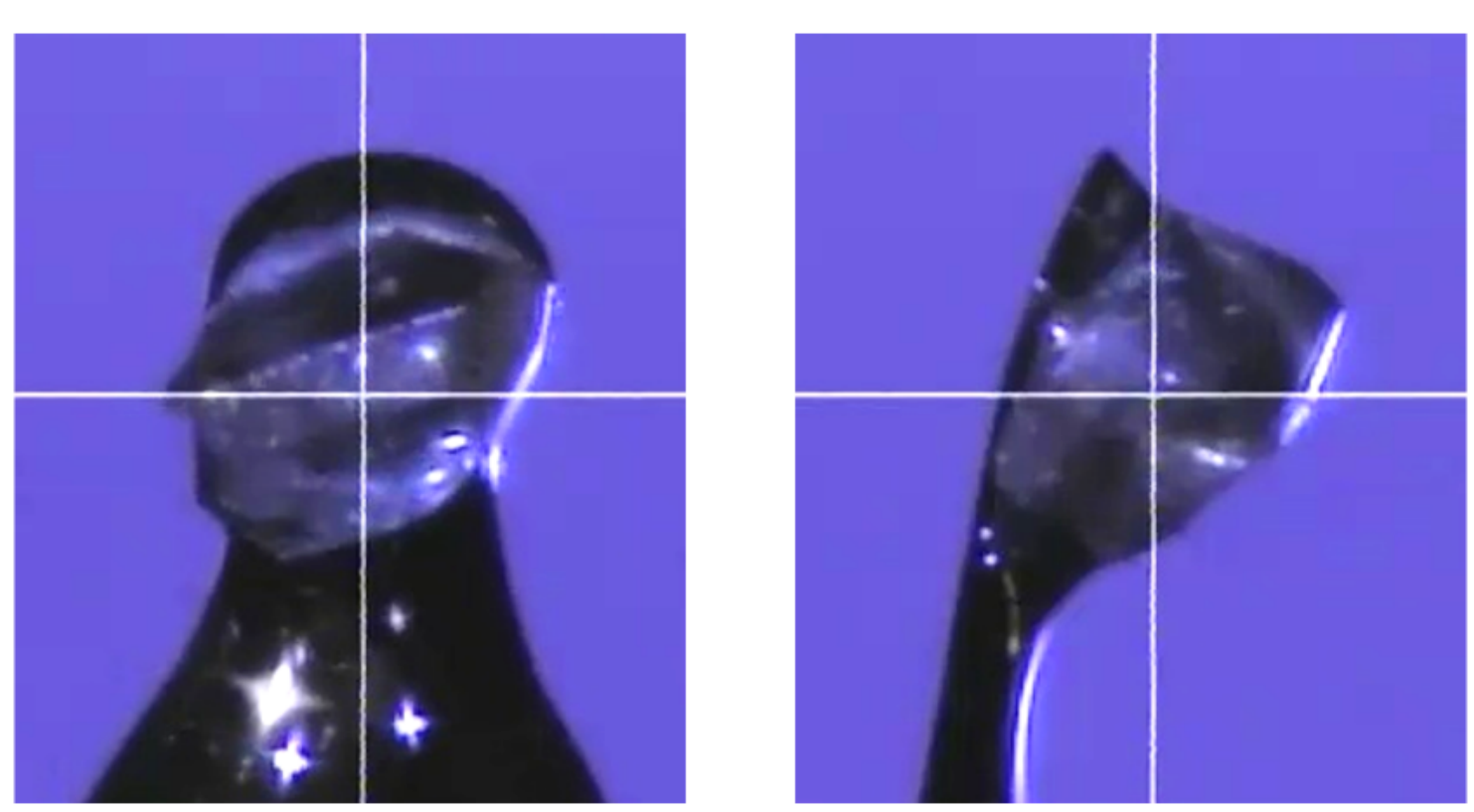

Figure S43. Optical images of the crystal of **1** used for SCXRD.

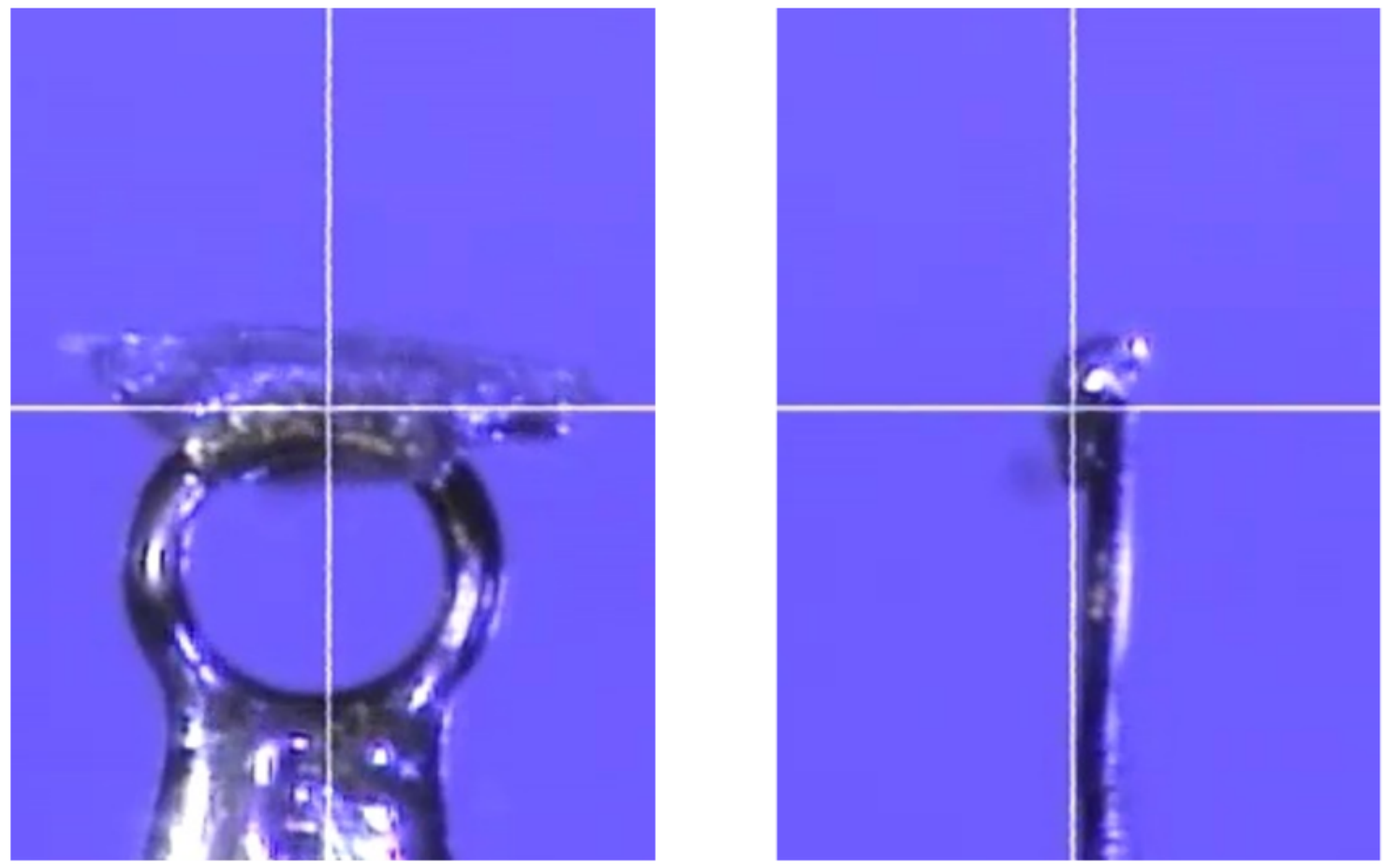

Figure S44. Optical images of the crystal of **1-Dimer** used for SCXRD.

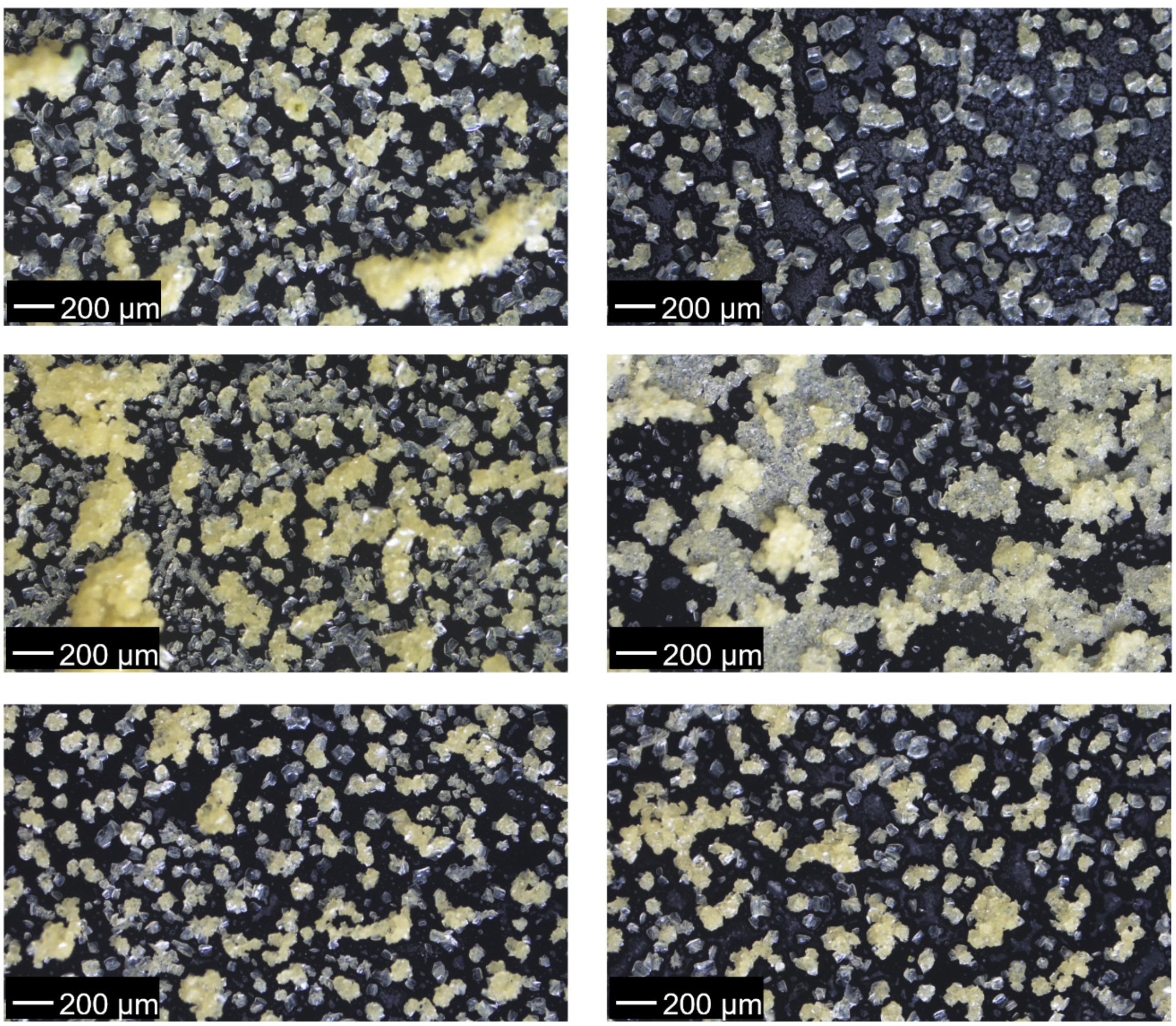

Figure S45. Optical images of **1** grown by evaporation from THF solution on a Pyrex dish.

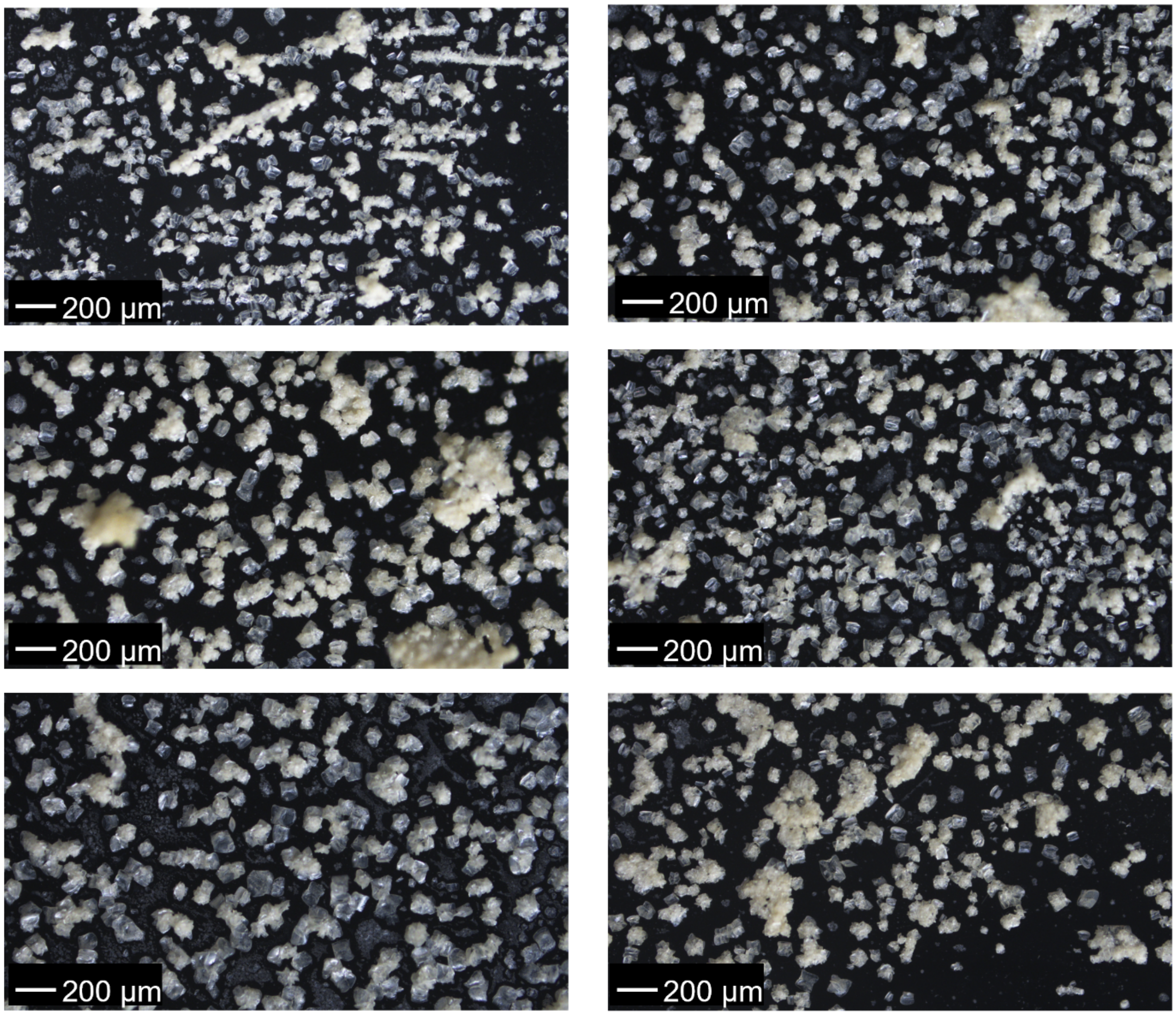

Figure S46. Optical images of **1-Dimer** synthesized by irradiation of dish-cast microcrystals of **1** in Figure S34 with 365 nm light for 5 h under N_2.

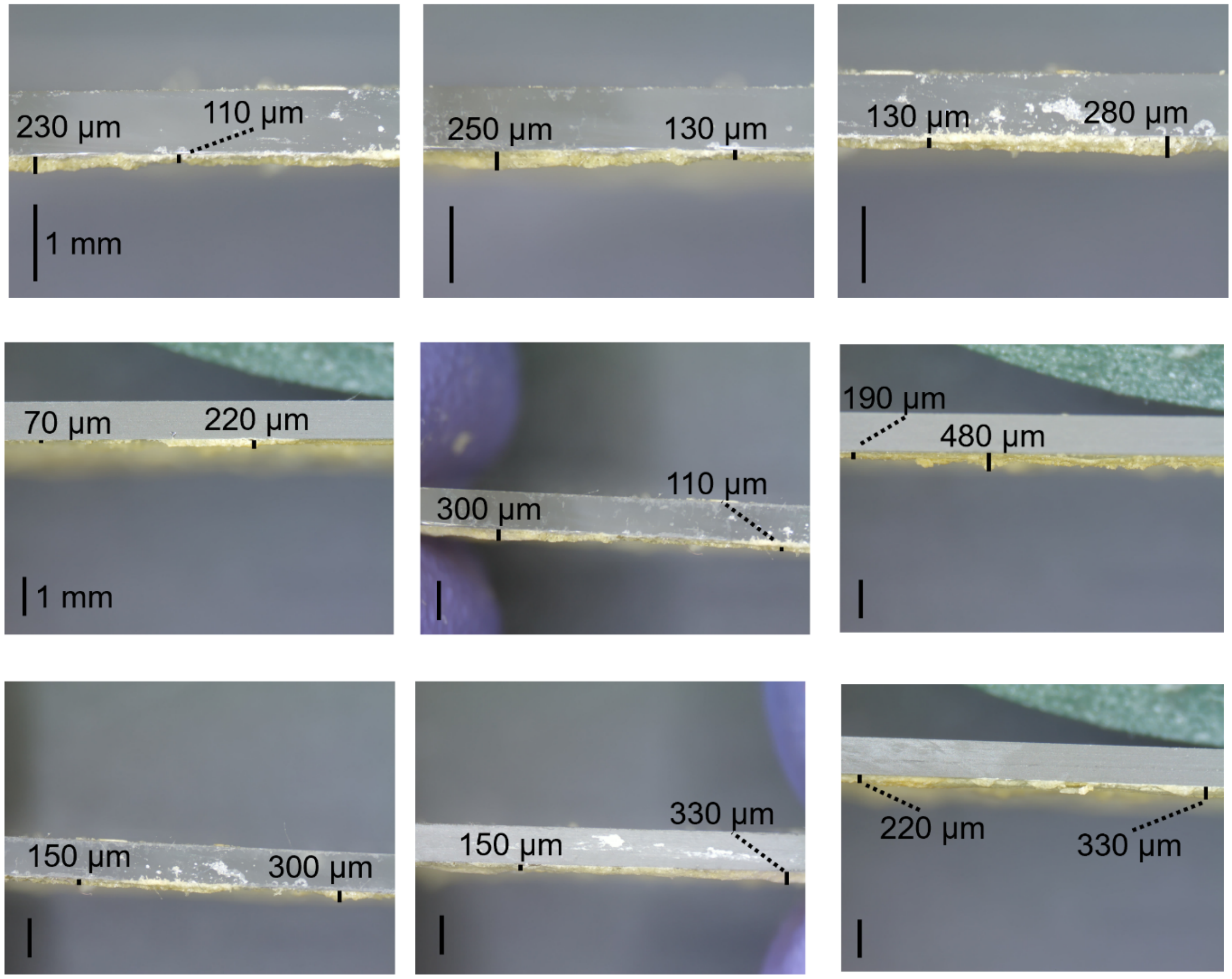

Figure S47. Stereoscope images of **1** cast on a glass slide to measure coating thickness at multiple points. Images note the maximum and minimum thickness of the coating for all regions measured.

I. Retrocyclization Kinetics

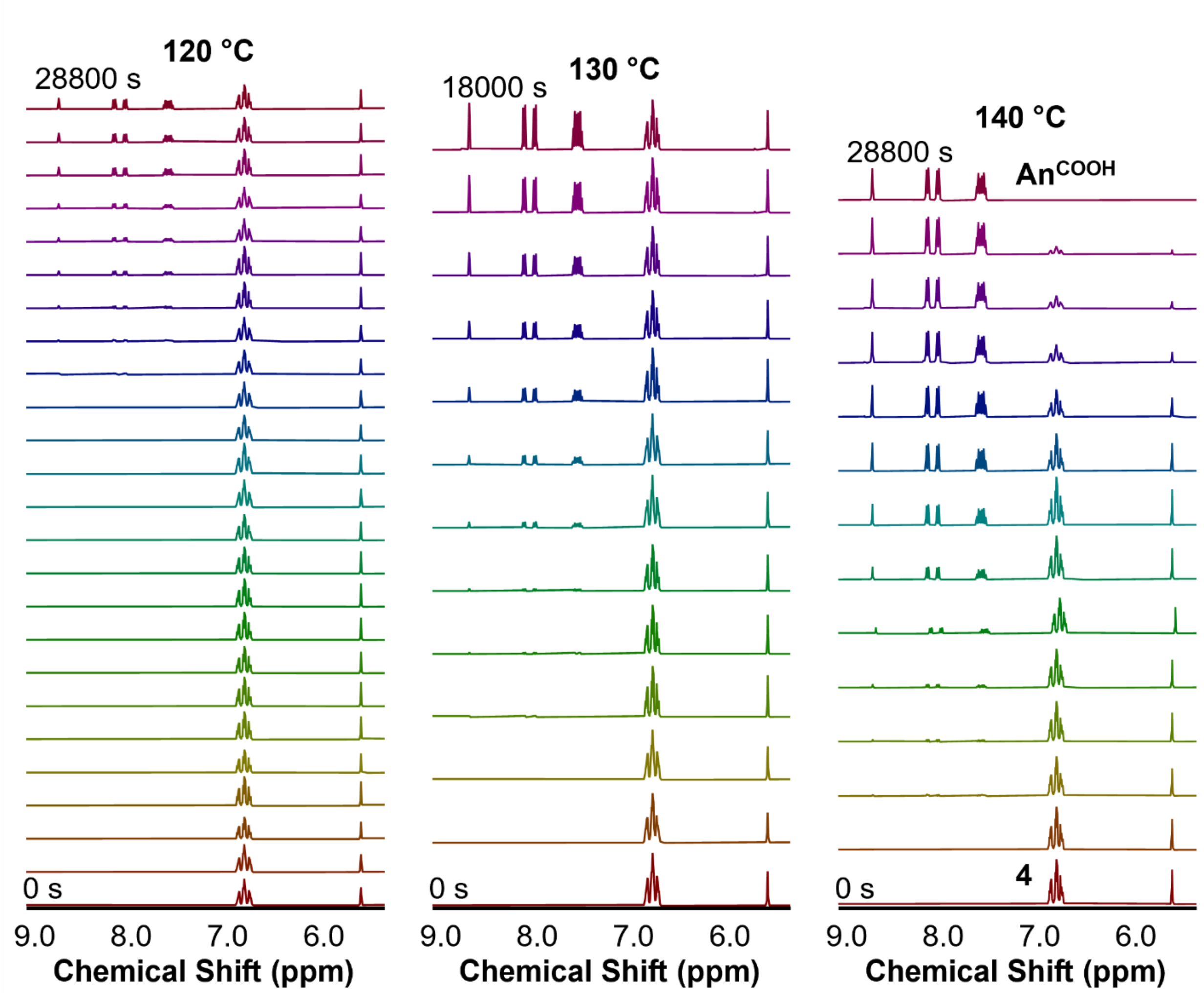

Figure S48. Solution-state retrocyclization kinetics data for **4** in DMSO-d6 at 120, 130, and 140 °C.

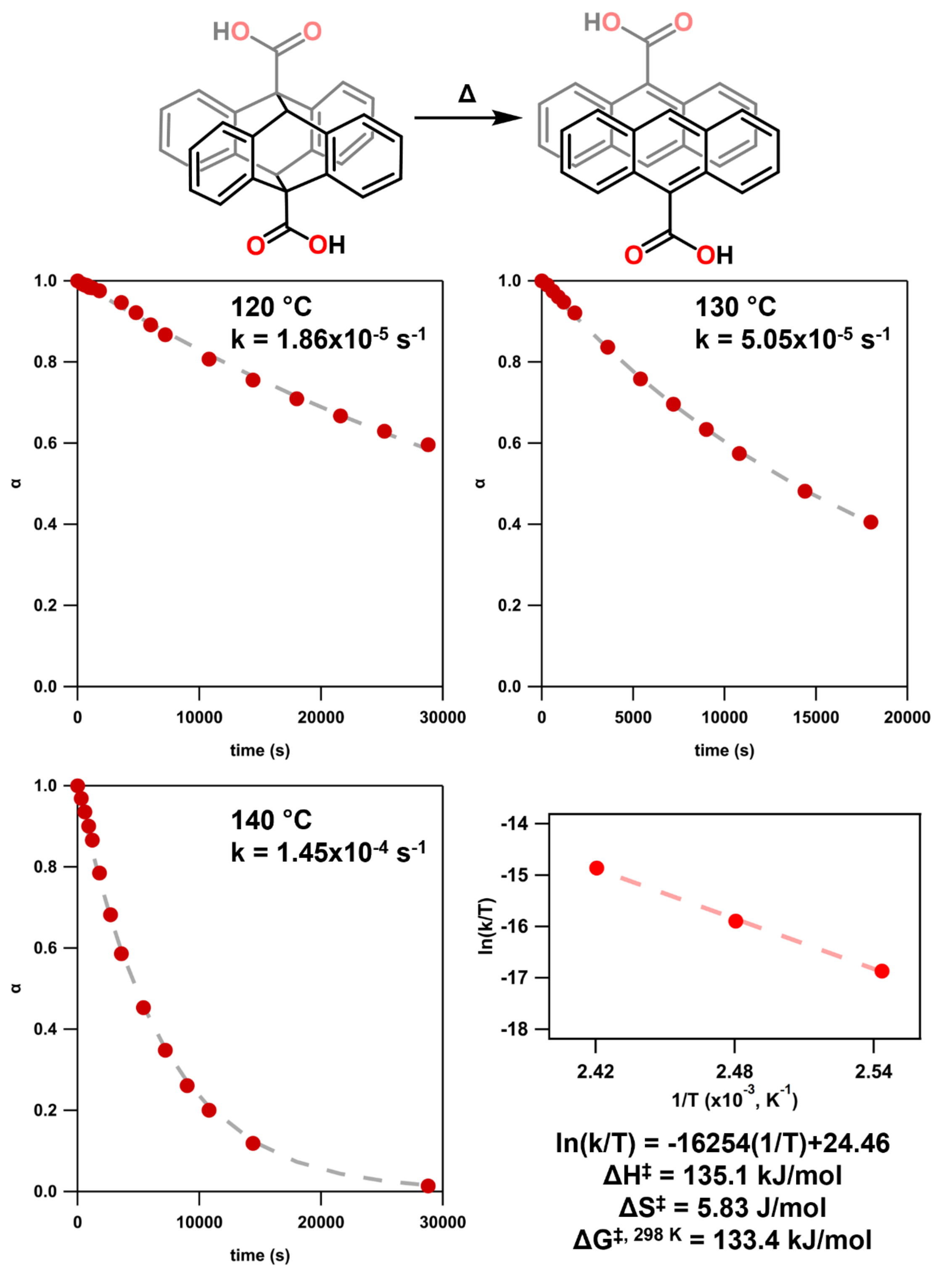

Figure S49. Kinetic traces and Eyring plot for retrocyclization of **4** in DMSO-d6 at 120, 130, and 140 °C.

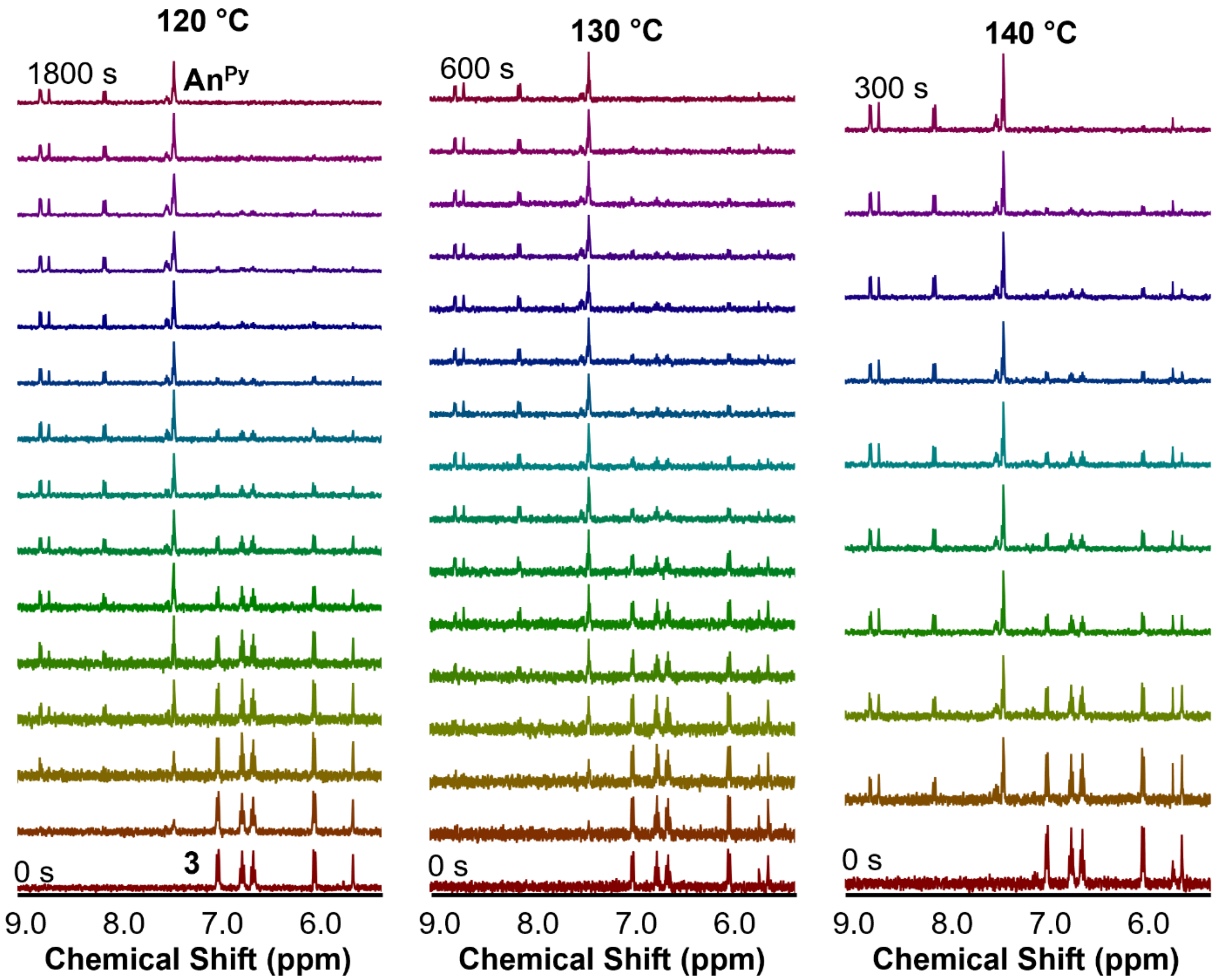

Figure S50. Solution-state retrocyclization kinetics data for **3** in DMSO-d6 at 120, 130, and 140 °C.

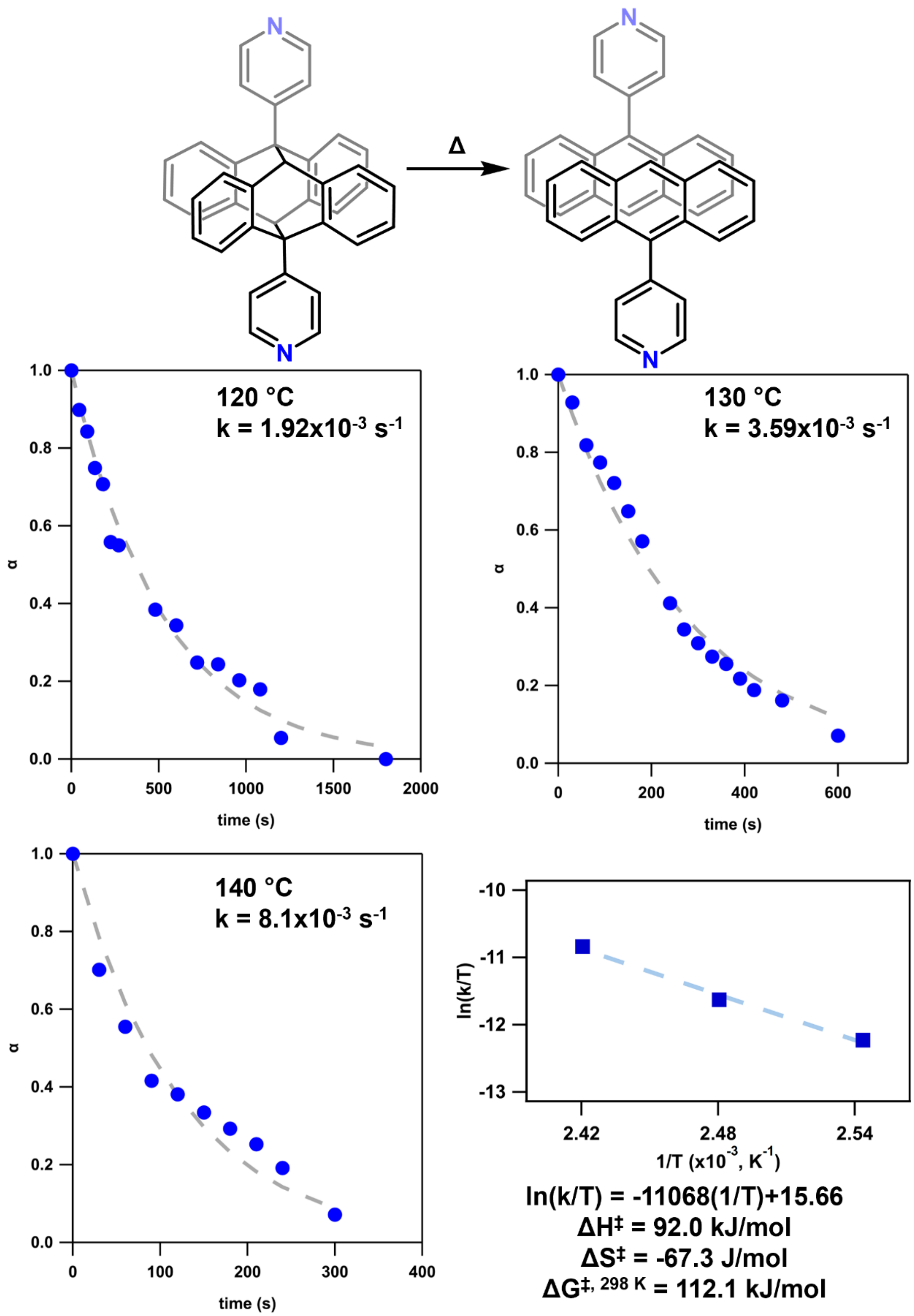

Figure S51. Kinetic traces and Eyring plot for retrocyclization of **3** in DMSO-d6 at 120, 130, and 140 °C.

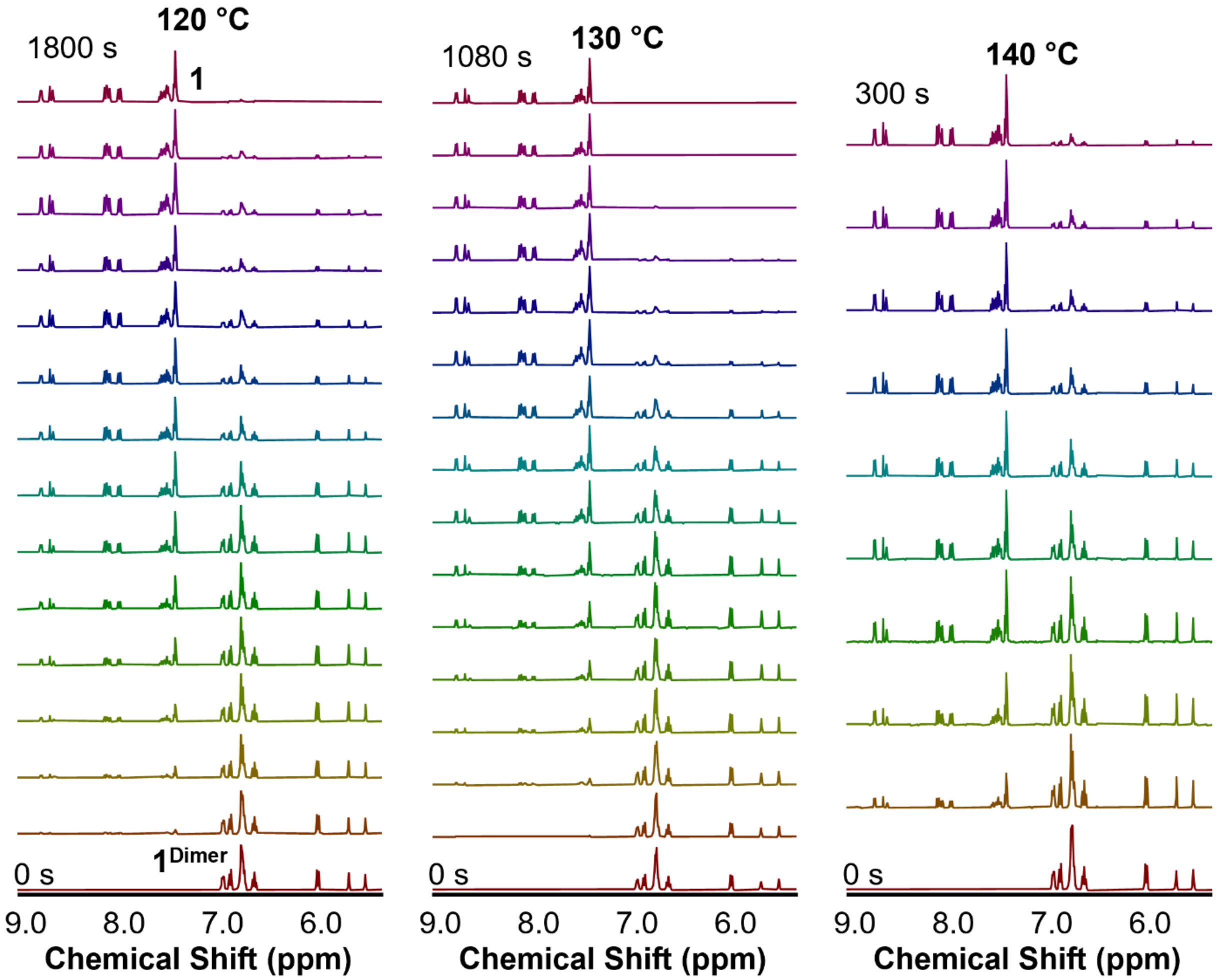

Figure S52. Kinetic traces and Eyring plot for retrocyclization of **2** in DMSO-d6 at 120, 130, and 140 °C.

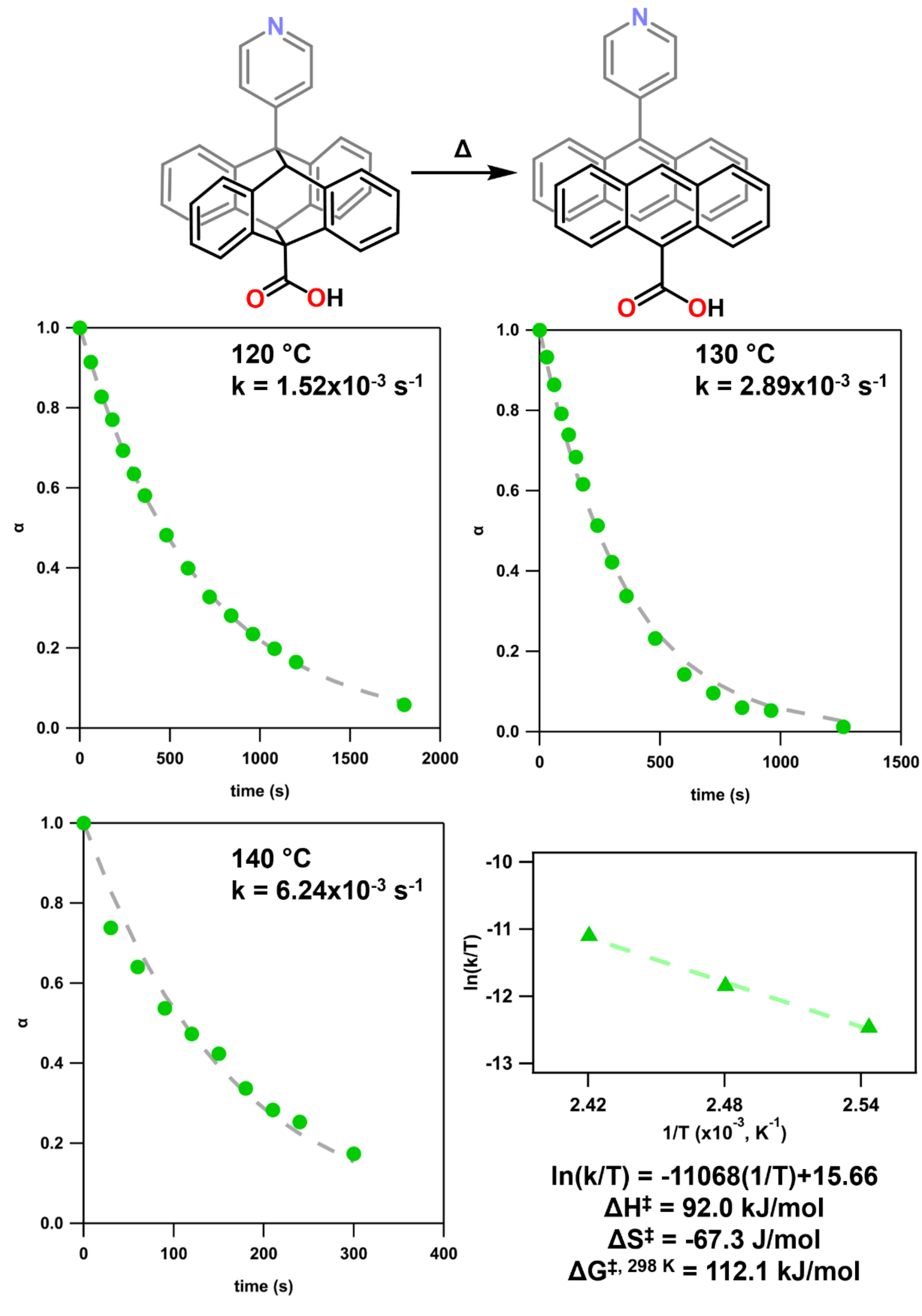

Figure S53. Kinetic traces and Eyring plot for retrocyclization of **2** in DMSO-d6 at 120, 130, and 140 °C.

J. References

1. O. V. Dolomanov, L. J. Bourhis, R. J. Gildea, J. A. K. Howard and H. Puschmann, *J. Appl. Cryst.*, 2009, **42**, 339–341.
2. G. Sheldrick, *Acta Cryst. A*, 2015, **71**, 3–8.
3. G. Sheldrick, *Acta Cryst. C*, 2015, **71**, 3–8.
4. J. Unge, B. L. Nannenga, A. G. Oliver and T. Gonen, *Acta Crystallographica Section C*, 2025, **81**, 376–390.
5. F. Neese, *J. Comput. Chem.*, 2003, **24**, 1740–1747.
6. F. Neese, F. Wennmohs, A. Hansen and U. Becker, *Chem. Phys.*, 2009, **356**, 98–109.
7. B. Helmich-Paris, B. de Souza, F. Neese and R. Izsák, *J. Chem. Phys.*, 2021, **155**.
8. F. Neese, *WIREs Comput. Mol. Sci.*, 2022, **12**, e1606.
9. Y. Li, S. S. Rajasree, G. Y. Lee, J. Yu, J.-H. Tang, R. Ni, G. Li, K. N. Houk, P. Deria and P. J. Stang, *J. Am. Chem. Soc.*, 2021, **143**, 2908–2919.
10. G. Collet, T. Lathion, C. Besnard, C. Piguet and S. Petoud, *J. Am. Chem. Soc.*, 2018, **140**, 10820–10828.
11. T. Salzillo, E. Venuti, C. Femoni, R. G. Della Valle, R. Tarroni and A. Brillante, *Cryst. Growth Des.*, 2017, **17**, 3361–3370.
